# Supplementary material for: Flattened 1D fragments of fullerene C60 that exhibit robustness toward multi-electron reduction
Source: Nat Commun. 2023 May 15;14:2741. doi: 10.1038/s41467-023-38300-3 (PMC10185694; doi:10.1038/s41467-023-38300-3)
Supplement: Supplementary file 1 — Supplementary Information [file 41467_2023_38300_MOESM1_ESM.pdf]

Supplementary Information

Flattened 1D fragments of fullerene C_{60} that exhibit robustness toward multi-electron reduction

Masahiro Hayakawa, Naoyuki Sunayama, Shu I. Takagi, Yu Matsuo, Asuka Tamaki, Shigehiro Yamaguchi, Shu Seki, and Aiko Fukazawa*

Table of Contents

1. C_{60} Fragment and Relevant Molecules

Supplementary Fig. 1: Representative examples of π-conjugated hydrocarbons that are composed of fragment structures of fullerenes S3

2. Reactivity of 5 toward Cross-Coupling Reactions

Supplementary Discussion 1: Attempts for cross-coupling reactions of **5** under the Kumada-Tamao-Corriu and Negishi conditions S4

Supplementary Fig. 2: Reaction of **5** with arylzinc reagents S4

Supplementary Discussion 2: Miyaura-Ishiyama Borylation Reactions S5

Supplementary Table 1: Optimization of reaction conditions for Miyaura-Ishiyama borylation of **5** S6

3. Characterization

Supplementary Data 1: Characterization data of structurally-novel compounds S7

4. X-ray Crystallographic Analyses

Supplementary Methods: X-ray crystallographic analysis S9

Supplementary Figs. 3 and 4: Crystal structures of **3a**, **4a**, **6a**, **7a**, and **8** S11

Supplementary Table 2: Selected C–C bond lengths of oligo(biindenylidene)s **3a**, **3b**, **4a**, and **4b** determined by X-ray crystallographic analyses S12

Supplementary Tables 3–6: Selected bond angles and the sum of them around each carbon atom in five-membered rings of **3a**, **3b**, **4a**, and **4b** S13

5. Electrochemical Properties

Supplementary Figs. 5 and 6: Cyclic voltammograms of **3a**–**c** and **4b** S17

Supplementary Table 7: Electrochemical data for oligo(biindenylidene)s **3a**–**c** and **4a**–**c** S18

Supplementary Discussion 3: Evaluation of the first reductive wave of **4c** S18

6. Photophysical Properties

Supplementary Fig. 7: Photophysical properties of oligo(biindenylidene)s **4a**–**c** compared with that of C_{60} S19

Supplementary Table 8: Summary of photophysical data of **3a**–**c** and **4a**–**c** S19

(To be continued to the next page)

(Table of Contents, continued)

7. Quantum Chemical Calculations

Supplementary Fig. 8: Benchmark results of DFT calculations for oligo(biindenylidenes) **4a′–c′** using various density functionals S20

Supplementary Fig. 9: Comparison of the electronic structures of oligo(biindenylidene)s **4a′**–**c′** with that of C_{60} S20

Supplementary Tables 9 and 10: Selected bond lengths in the optimized geometries of charge-neutral biindenylidene monomers **3a** and **4a′**, and dimers **3a** and **4a′** together with the corresponding experimental data S21

Supplementary Table 11: Selected bond lengths in the optimized geometries of charge-neutral biindenylidene trimers **3c** and **4c′** S22

Supplementary Figs. 10: Plot of selected bond lengths in the optimized geometries of charge-neutral oligo(biindenylidene)s **3a**–**c** and **4a′**–**4c′** S22

Supplementary Fig. 11: Plot of selected bond lengths in the optimized geometries of charge-neutral oligo(biindenylidene)s **4a′** and **4b′**, and the corresponding radical anions [**4a′**]$^{•–}$ and [**4b′**]$^{•–}$ and dianions [**4a′**]$^{2–}$ and [**4b′**]$^{2–}$ S23

Supplementary Fig. 12: Pictorial representation of the Mulliken spin densities of oligo(biindenylidene) radical anions [**4a′**]$^{•–}$ and [**4b′**]$^{•–}$ S23

Supplementary Fig. 13: The NICS(0) values of **4b′** and [**4b′**]$^{2–}$ S24

Supplementary Table 12: Selected data of the TD-DFT vertical excitation of oligo(biindenylidene)s **3** and **4′** S24

Supplementary Discussion 4: Assignments of the electronic absorption of **3c** S24

Supplementary Fig. 14: Comparison of the experimental electronic absorption of **3c** with the calculated TD-DFT vertical excitations S25

Supplementary Discussion 5: Insights into the structural changes of oligo(biindenylidene)s upon reduction. S25

8. Time-Resolved Microwave Conductivity Measurements

Supplementary Fig. 15: Flash-Photolysis TRMC transients recorded for polycrystalline **3a**–**c** S26

Supplementary Fig. 16: Electronic absorption spectra of **3c** S26

9. Appendix

Supplementary Tables 13–35: Cartesian coordinates of the optimized geometries S27

Supplementary Figs. 17–39: NMR spectra of newly synthesized compounds S67

Supplementary Figs. 40–50: High-resolution mass spectra of newly synthesized compounds S79

10. Supplementary References S90

1. C_{60} Fragment and Relevant Molecules

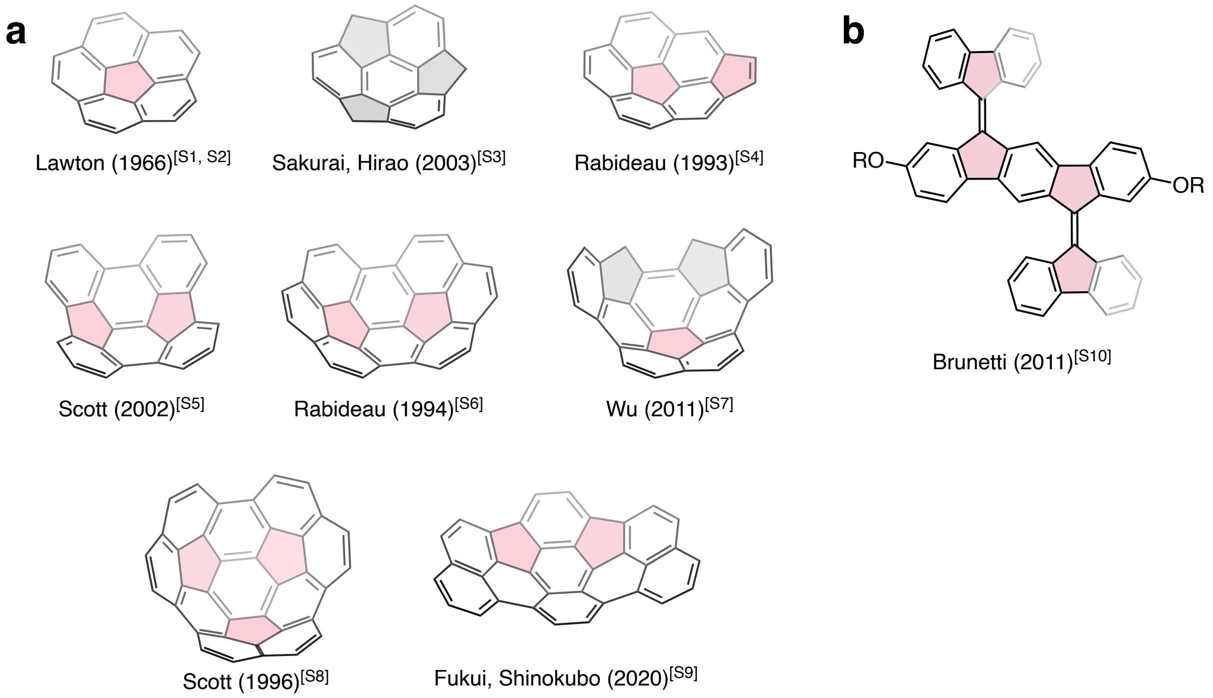

Supplementary Fig. 1: Representative examples of π-conjugated hydrocarbons that are composed of fragment structures of fullerenes. Five-membered ring substructures consisting entirely of sp^2 carbon atoms and those with at least one sp^3 carbon are highlighted in pink and gray, respectively. Each reference number corresponds to a citation number in the manuscript. **a.** Bowl-shaped molecules with fragment structures of C_{60} or C_{70}. **b.** A fragment molecule with twist-flatten geometry.

2. Reactivity of 5 toward Cross-Coupling Reactions

Supplementary Discussion 1: Attempts for Cross-Coupling Reactions of 5 under the Kumada-Tamao-Corriu and Negishi Conditions. We initially examined the reactivity of **5** under the conditions of Kumada-Tamao-Corriu coupling with phenylmagnesium bromide by using Ni or Pd catalysts. However, all the reaction conditions resulted in the formation of a black-colored complex mixture without the formation of desired phenylated **3a** and **6a**. Given that a similar result was obtained even with the addition of phenylmagnesium bromide to **5** in the absence of any catalyst precursors, we assume that the single-electron transfer from Grignard reagent to **5** followed by the decomposition of the resulting radical anion species took place due to the highly electron-accepting character of **5**.

Assuming that undesirable electron transfer reaction from arylmetal reagents to **5** should be suppressed for successful cross-coupling reactions, we next investigated the conditions for a typical Negishi cross-coupling reaction. Initially, we attempted the synthesis of monoarylated 1,1′-biindenylidenes (**6a** and **7a**) as end-capping building blocks by Pd-catalyzed Negishi coupling of dibromide **5** with arylzinc chloride (Supplementary Fig. 2). However, careful separation of the products provided the expected cross-coupling products, such as monophenylated biindenylidene **6a** (15% yield) and diphenylated biindenylidene **3a** (4% yield), and the unexpected oligomers including the monophenylated biindenylidene dimer (**6b**, 1% yield), diphenylated biindenylidene dimer (**3b**, 1% yield), and trimer (**3c**, 0.9% yield). The reaction using trialkylsilyl-substituted phenylzinc chloride gave results identical to those described above, and the corresponding oligo(biindenylidene)s with silyl groups at both termini (**4a**–**c**) were obtained (Supplementary Fig. 2).

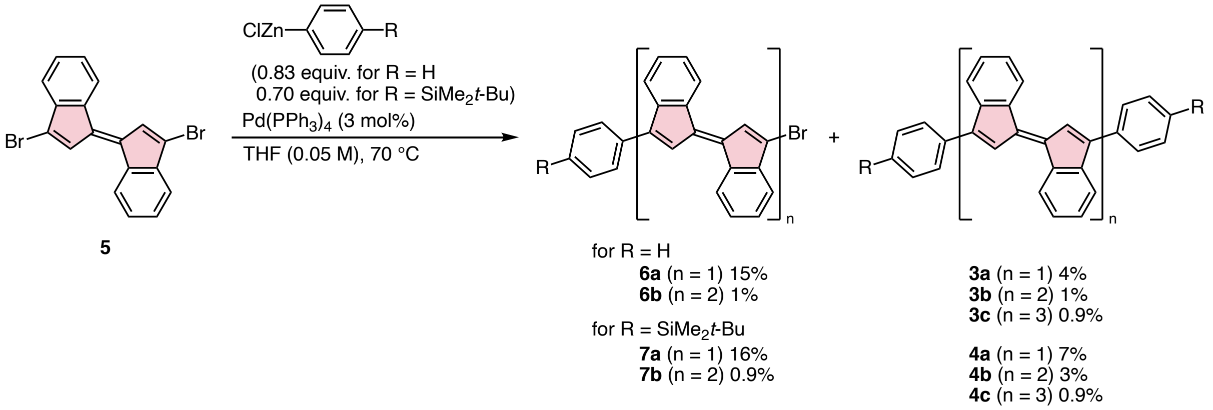

Supplementary Fig. 2: Reaction of 5 with arylzinc reagents.

Supplementary Discussion 2: Miyaura-Ishiyama Borylation Reactions. For the synthesis of oligo(biindenylidenes) by iterative cross-coupling reactions, the biindenylidenes bearing organometallic functional groups such as boronic acid or boronate are essential precursors. However, preliminary investigations indicated that the conventional transformation reactions of aryl and/or vinyl halides by halogen-metal exchange reaction followed by the trapping with electrophiles are not feasible for dibrominated **5** because of the low solubility and the concomitant electron transfer reactions (see also Supplementary Discussion 1). Therefore, among the already known transformations of C(sp^2)–Br bond to the corresponding organoboronates, we focused on Miyaura-Ishiyama borylation,[S11] which does not require strong reducing reagents or low-temperature conditions. In the presence of bis(pinacolato)dibiron (B_2pin_2) as a boron source, several different reaction conditions including a Pd source, ligand, base, and solvent were examined (Supplementary Table 1). The use of $PdCl_2$(dppf), the most commonly used catalyst precursor for Miyaura-Ishiyama borylation, did not give the desired product **8** regardless of the choice of base, solvent, or temperature, albeit complete consumption of **5** (entries 1–6). After the exhaustive screenings of Pd source and ligands, only $Pd(PPh_3)_4$ was found to give the desired product **8** (entries 18 and 19) in contrast to several other catalyst precursors such as $Pd(OAc)_2$ (entries 7–11), $Pd_2(dba)_3$ (entries 12 and 13), $PdCl_2(PPh_3)_2$ (entry 15), and XPhos Pd G3 (entries 16 and 17). Specifically, **8** was obtained in 40% yield when potassium acetate and THF were used as a base and solvent (entries 18 and 19), whereas no target product was observed with potassium phenoxide (entry 21). In addition, while the higher reaction temperature improved the yield of **8** in THF (entries 18 and 19), **8** was not obtained under heating in 1,4-dioxane at 110 °C (entry 20). Control experiments in the absence of B_2pin_2 showed that diboronate **8** is gradually decomposed under reaction conditions. The lack of stability of **8** might be partly responsible for the low yielding of **8** in only 40%. Given these results, we selected the *in-situ* preparation of the monoboronates derived from **6a** and **7a** and successive Suzuki-Miyaura coupling for the synthesis of biindenylidene dimers **3b** and **4b**. This strategy was successful as can be seen in the reasonably high yields of **3b** (70%) and **4b** (55%).

Supplementary Table 1: Optimization of reaction conditions for Miyaura-Ishiyama borylation of **5**[a]

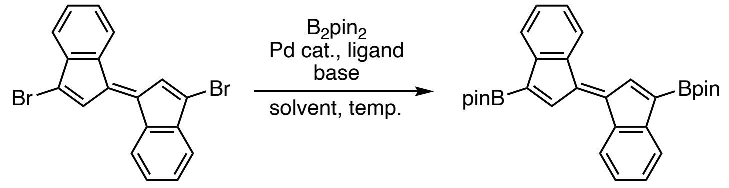

5 **8**

Entry	Pd cat.	Ligand	Base	Solvent	Temp.	Yield
1	$PdCl_2$(dppf)	none	KOAc	DMSO	80 °C	n.d.[c]
2	$PdCl_2$(dppf)	none	KOAc	1,4-dioxane	80 °C	n.d.[c]
3	$PdCl_2$(dppf)	none	KOAc	THF	80 °C	n.d.[c]
4	$PdCl_2$(dppf)	none	KOAc	toluene	110 °C	n.d.[c]
5	$PdCl_2$(dppf)	none	KOPh	DMSO	50 °C	n.d.[c]
6	$PdCl_2$(dppf)	none	KOPh	toluene	110 °C	n.d.[c]
7	$Pd(OAc)_2$	SIPr	KOAc	toluene	110 °C	n.d.[c]
8	$Pd(OAc)_2$	JohnPhos	Et_3N	1,4-dioxane	100 °C	n.d.[c]
9	$Pd(OAc)_2$	DPEPhos	Et_3N	1,4-dioxane	100 °C	n.d.[c]
10	$Pd(OAc)_2$	JohnPhos	KOPh	toluene	50 °C	n.d.[c]
11	$Pd(OAc)_2$	DPEPhos	KOPh	toluene	50 °C	n.d.[c]
12	$Pd(OAc)_2$	XantPhos	KOPh	toluene	50 °C	n.d.[c]
13	$Pd_2(dba)_3$	PCy_3	KOAc	1,4-dioxane	80 °C	n.d.[c]
14	$Pd_2(dba)_3$	PCy_3	KOPh	toluene	50 °C	n.d.[c]
15	$PdCl_2(PPh_3)_2$	PPh_3	KOPh	toluene	80 °C	n.d.[c]
16	XPhos Pd G3	none	KOAc	toluene	110 °C	n.d.[c]
17	XPhos Pd G3	none	KOPh	toluene	50 °C	n.d.[c]
18[b]	$Pd(PPh_3)_4$	none	KOAc	THF	50 °C	16%
19[b]	$Pd(PPh_3)_4$	none	KOAc	THF	75 °C	40%
20[b]	$Pd(PPh_3)_4$	none	KOAc	1,4-dioxane	110 °C	n.d.[c]
21[b]	$Pd(PPh_3)_4$	none	KOPh	THF	75 °C	n.d.[c]

[a]B_2pin_2 (3 equiv.), Pd catalyst (5 mol%), ligand (5 mol%), and base (2 equiv.) were used. [b]B_2pin_2 (2.4 equiv.), Pd catalyst (2 mol%), and base (5 equiv.) were used. [c]Not detected.

3. Characterization

Supplementary Data 1: Characterization Data of Structurally-Novel Compounds

1-(*tert*-Butyldimethylsilyl)-4-(4,4,5,5-tetramethyl-1,3,2-dioxaborolan-2-yl)benzene. Mp: 112.7–113.4 °C. ^{1}H NMR (500 MHz, $CDCl_3$): δ 0.27 (s, 6H), 0.86 (s, 9H), 1.34 (s, 12H), 7.52 (dd, J = 6.4 Hz, 1.8 Hz, 2H), 7.77 (d, J = 7.9 Hz, 2H). ^{13}C{^{1}H} NMR (125 MHz, $CDCl_3$): δ –6.1, 17.0, 25.0, 26.6, 83.8, 129.2, 133.7, 133.9, 141.6. HRMS (ESI): *m*/*z* Calcd. for $C_{18}H_{32}BO_2Si$: 319.2263 ([*M*+H]$^+$). Obsd. 319.2261.

(*E*)-3,3′-Diphenyl-1,1′-biindenylidene (3a). Mp: 210.9–211.0 °C. ^{1}H NMR (500 MHz, $CDCl_3$): δ 7.29–7.34 (m, 4H), 7.44 (tt, J = 7.4 Hz, 1.3 Hz, 2H), 7.52 (dd, J = 8.3 Hz, 7.4 Hz, 4H), 7.55 (s, 2H), 7.59–7.62 (m, 2H), 7.76 (dd, J = 8.3 Hz, 1.3 Hz, 4H), 8.10–8.11 (m, 2H). ^{13}C{^{1}H} NMR (100 MHz, CD_2Cl_2): δ 121.7, 124.8, 125.8, 126.5, 128.2, 128.4, 129.1, 129.2, 135.6, 138.7, 140.4, 142.4, 149.1. HRMS (APCI(+)): *m*/*z* Calcd. for $C_{30}H_{21}$: 381.1638 ([*M*+H]$^+$). Obsd. 381.1637.

3,3‴-Diphenylquaterindene (3b). Mp: 239.5–239.8 °C. ^{1}H NMR (500 MHz, $CDCl_3$): δ 7.32–7.36 (m, 4H), 7.39 (td, J = 7.5 Hz, 1.2 Hz, 2H), 7.43–7.48 (m, 4H), 7.53 (dd, J = 8.4 Hz, 7.5 Hz, 4H), 7.57 (s, 2H), 7.62–7.63 (m, 2H), 7.79 (dd, J = 8.4 Hz, 1.5 Hz, 4H), 7.81 (d, J = 7.4 Hz, 2H), 7.95 (s, 2H), 8.09–8.10 (m, 2H), 8.16 (d, J = 7.6 Hz, 2H). ^{13}C{^{1}H} NMR (125 MHz, $CDCl_3$): δ 121.7, 121.8, 124.7, 125.5, 126.3, 126.5, 126.7, 128.0, 128.3, 128.4, 128.9, 129.0, 135.5, 138.4, 138.5, 140.5, 141.3, 141.8, 142.3, 142.4, 149.5. One signal in the aromatic region was not observed due to overlap with the other signals. HRMS (MALDI(+)): *m*/*z* Calcd. for $C_{48}H_{30}$: 606.2342 ([*M*]$^+$), Obsd. 606.2346.

3,3‴‴-Diphenylsexiindene (3c). Mp: >300 °C. ^{1}H NMR (600 MHz, TCE-d_2, 80 °C): δ 7.38–7.41 (m, 4H), 7.44–7.48 (m, 4H), 7.51 (t, J = 7.4 Hz, 6H), 7.58 (dd, J = 7.5 Hz, 7.3 Hz, 4H), 7.62 (s, 2H), 7.66–7.67 (m, 4H), 7.83 (dd, J = 7.3 Hz, 1.1 Hz, 4H), 7.86–7.88 (m, 4H), 8.02 (s, 2H), 8.03 (s, 2H), 8.14–8.15 (m, 2H), 8.19–8.21 (m, 4H). ^{13}C{^{1}H} NMR (150 MHz, TCE-d_2, 80 °C): δ 121.5, 121.6, 121.8, 124.4, 125.2, 125.3, 126.1, 126.31, 126.32, 126.5, 126.7, 127.7, 128.1, 128.2, 128.4, 128.69, 128.71, 135.1, 138.05, 138.10, 138.2, 140.1, 141.25, 141.31, 141.5, 142.02, 142.08, 142.10, 142.2, 149.5. One signal in the aromatic region was not observed due to overlap with the other signals. HRMS (MALDI(+)): *m*/*z* Calcd. for $C_{66}H_{40}$: 832.3125 ([*M*]$^+$). Obsd. 832.3125.

(*E*)-3,3′-Bis[4-(*tert*-butyldimethylsilyl)phenyl]-1,1′-biindenylidene (4a). Mp: 260.7–261.3 °C. ^{1}H NMR (500 MHz, $CDCl_3$): δ 0.34 (s, 12H), 0.94 (s, 18H), 7.28–7.33 (m, 4H), 7.56 (s, 2H), 7.63–7.65 (m, 2H), 7.66 (d, J = 8.0 Hz, 4H), 7.74 (d, J = 8.0 Hz, 4H), 8.09–8.11 (m, 2H). ^{13}C{^{1}H} NMR (125 MHz, $CDCl_3$): δ –6.0, 17.2, 26.7, 121.5, 124.8, 125.5, 126.2, 126.8, 128.0, 135.0, 135.8, 138.6, 138.9, 140.3, 142.2, 148.8. HRMS (APCI(+)): *m*/*z* Calcd. for $C_{42}H_{49}Si_2$: 609.3367 ([*M*+H]$^+$). Obsd. 609.3368.

3,3‴-Bis[4-(*tert*-butyldimethylsilyl)phenyl]quaterindene (4b). Mp: >300 °C. ^{1}H NMR (500 MHz, $CDCl_3$): δ 0.34 (s, 12H), 0.95 (s, 18H), 7.33–7.35 (m, 4H), 7.38 (td, J = 7.6 Hz, 1.2 Hz, 2H), 7.44 (td, J = 7.6 Hz, 1.1 Hz, 2H), 7.59 (s, 2H), 7.65–7.66 (m, 2H), 7.67 (d, J = 8.1 Hz, 4H), 7.76 (d, J = 8.1 Hz, 4H), 7.81 (d, J = 7.6 Hz, 2H), 7.95 (s, 2H), 8.08–8.10 (m, 2H), 8.15 (d, J = 7.6 Hz, 2H). ^{13}C{^{1}H} NMR (125 MHz, $CDCl_3$): δ –6.0, 17.2, 26.7, 121.7, 121.8, 124.8, 125.5, 126.3, 126.5, 126.6, 126.8, 128.3,

128.4, 135.1, 135.7, 138.4, 138.6, 139.2, 140.5, 141.4, 141.8, 142.2, 142.4, 149.5. One signal in the aromatic region was not observed due to overlap with the other signals. HRMS (APCI(+)): *m*/*z* Calcd. For $C_{60}H_{59}Si_2$: 835.4150 ([*M*+H]$^+$), Obsd. 835.4150.

3,3′′′′′-Bis[4-(*tert*-butyldimethylsilyl)phenyl]sexiindene (4c). Mp: >300 °C. ^{1}H NMR (600 MHz, TCE-*d*$_2$, 80 °C): δ 0.40 (s, 12H), 1.01 (s, 18H), 7.38–7.41 (m, 4H), 7.43–7.47 (m, 4H), 7.51 (t, *J* = 7.4 Hz, 4H), 7.64 (s, 2H), 7.70–7.72 (m, 2H), 7.73 (d, *J* = 8.0 Hz, 4H), 7.81 (d, *J* = 8.0 Hz, 4H), 7.86–7.88 (m, 4H), 8.02 (s, 2H), 8.03 (s, 2H), 8.13–8.15 (m, 2H), 8.20 (t, *J* = 8.1 Hz, 2H), 8.21 (t, *J* = 8.0 Hz, 2H). ^{13}C{^{1}H} NMR (125 MHz, TCE-*d*$_2$, 80 °C): δ –6.2, 16.7, 26.5, 121.6, 121.8, 124.4, 125.16, 125.23, 125.3, 126.0, 126.3, 126.47, 126.52, 126.7, 128.1, 128.2, 128.4, 134.7, 135.2, 138.06, 138.11, 138.3, 139.1, 140.1, 141.2, 141.4, 141.5, 142.01, 142.05, 142.10, 142.2, 149.5. Two signals attributable to the carbon atoms in the aromatic region were not observed due to overlap with the other signals. HRMS (MALDI(+)): *m*/*z* Calcd. for $C_{78}H_{68}Si_2$: 1060.48541 ([*M*]$^+$), Obsd. 1060.48413.

(*E*)-3-Bromo-3′-phenyl-1,1′-biindenylidene (6a). Mp: 171.1–171.8 °C. ^{1}H NMR (500 MHz, $CDCl_3$): δ 7.29–7.34 (m, 3H), 7.36–7.41 (m, 2H), 7.42 (s, 1H), 7.45 (tt, *J* = 7.5 Hz, 1.4 Hz, 1H), 7.51 (dd, *J* = 8.2 Hz, 7.5 Hz, 2H), 7.58 (dd, *J* = 7.1 Hz, 1.8 Hz, 1H), 7.64 (s, 1H), 7.74 (dd, *J* = 8.2 Hz, 1.4 Hz, 2H), 7.99 (d, *J* = 7.5 Hz, 2H). ^{13}C{^{1}H} NMR (125 MHz, $CDCl_3$): δ 121.0, 121.6, 124.4, 124.7, 125.4, 126.5, 127.1, 127.4, 127.9, 128.38, 128.47, 128.50, 128.95, 128.97, 135.3, 136.1, 138.11, 138.12, 140.2, 141.2, 142.3, 149.9. HRMS (APCI(+)): *m*/*z* Calcd. for $C_{24}H_{16}{}^{79}Br$: 383.0430 ([*M*+H]$^+$). Obsd. 383.0427.

3-Bromo-3′′′-phenylquaterindene (6b). Mp: >300 °C. ^{1}H NMR (500 MHz, CD_2Cl_2): δ 7.35–7.43 (m, 7H), 7.44–7.49 (m, 3H), 7.55 (dd, *J* = 8.4 Hz, 7.4 Hz, 2H), 7.59 (s, 1H), 7.63–7.65 (m, 1H), 7.71 (s, 1H), 7.80–7.84 (m, 4H), 7.88 (s, 1H), 7.99 (s, 1H), 8.05 (d, *J* = 7.3 Hz, 1H), 8.08 (d, *J* = 7.7 Hz, 1H), 8.12 (m, 1H), 8.19 (d, *J* = 7.4 Hz, 1H). ^{13}C{^{1}H} NMR spectrum could not be obtained due to its poor solubility. HRMS (MALDI(+)): *m*/*z* Calcd. for $C_{42}H_{25}{}^{79}Br$: 608.113414 ([*M*]$^+$), Obsd. 608.113480.

(*E*)-3-Bromo-3′-[4-(*tert*-butyldimethylsilyl)phenyl]-1,1′-biindenylidene (7a). Mp: 185.9–186.3 °C. ^{1}H NMR (500 MHz, $CDCl_3$): δ 0.34 (s 6H), 0.94 (s, 9H), 7.29–7.34 (m, 3H), 7.35–7.41 (m, 2H), 7.44 (s, 1H), 7.61 (dd, *J* = 6.3 Hz, 1.8 Hz, 1H), 7.64 (s, 1H), 7.65 (d, *J* = 8.0 Hz, 2H), 7.71 (d, *J* = 8.0 Hz, 2H), 7.99 (d, *J* = 7.3 Hz, 2H). ^{13}C{^{1}H} NMR (125 MHz, $CDCl_3$): δ –6.0, 17.2, 26.7, 121.0, 121.7, 124.5, 124.7, 125.4, 126.5, 126.7, 127.1, 127.4, 128.35, 128.46, 128.48, 135.0, 135.6, 136.1, 138.1, 138.2, 139.2, 140.3, 141.2, 142.3, 150.0. HRMS (APCI(+)): *m*/*z* Calcd. for $C_{30}H_{30}Si^{79}Br$: 497.1295 ([*M*+H]$^+$). Obsd. 497.1292.

3-Bromo-3′′′-[4-(*tert*-butyldimethylsilyl)phenyl]quaterindene (7b). Mp: >300 °C. ^{1}H NMR (500 MHz, CD_2Cl_2): δ 0.35 (s, 6H), 0.95 (s, 9H), 7.33–7.47 (m, 9H), 7.59 (s, 1H), 7.65–7.67 (m, 1H), 7.69 (d, *J* = 8.1 Hz, 2H), 7.69 (s, 1H), 7.77–7.82 (m, 2H), 7.78 (d, *J* = 8.1 Hz, 2H), 7.86 (s, 1H), 7.97 (s, 1H), 8.03 (d, *J* = 7.4 Hz, 1H), 8.06 (d, *J* = 7.6 Hz, 1H), 8.09–8.11 (m, 1H), 8.17 (d, *J* = 7.5 Hz, 1H). ^{13}C{^{1}H} NMR (125 MHz, CD_2Cl_2): δ –6.0, 17.3, 26.7, 121.3, 122.0, 122.1, 122.3, 125.0, 125.6, 125.8, 126.3, 126.85, 127.00, 127.02, 127.2, 127.7, 127.8, 128.67, 128.71, 129.0, 129.16, 129.21, 135.4, 135.8, 136.2, 138.1, 138.5, 138.7, 139.2, 139.7, 140.5, 140.8, 141.5, 141.8, 141.9, 142.3, 142.4, 142.6, 143.1, 150.0.

Three signals in the aromatic region were not observed due to overlap with the other signals . HRMS (MALDI(+)): *m*/*z* Calcd. for $C_{48}H_{40}{}^{79}BrSi$: 723.2077 ([*M*+H]$^+$), Obsd. 723.2077.

(*E*)-3,3′-Bis(4,4,5,5-tetramethyl-1,3,2-dioxaborolan-2-yl)-1,1′-biindenylidene (8). Mp: 273.5–274.3 °C. ^{1}H NMR (500 MHz, CD_2Cl_2): δ 1.38 (s, 24H), 7.21 (td, J = 7.5 Hz, 1.3 Hz, 2H), 7.27 (td, J = 7.5 Hz, 1.1 Hz, 2H), 7.72 (d, J = 6.8 Hz, 2H), 7.91 (s, 2H), 8.00 (d, J = 7.8 Hz, 2H). ^{13}C{^{1}H} NMR (125 MHz, $CDCl_3$): δ 25.1, 83.8, 123.6, 125.7, 125.8, 128.8, 137.3, 139.2, 143.1, 146.3. The signal of the carbon atoms bound to the boron atom was not observed due to the quadrupolar relaxation. HRMS (APCI(+)): *m*/*z* Calcd. for $C_{30}H_{35}B_2O_4$: 481.2716 ([*M*+H]$^+$), Obsd. 481.2722.

4. X-ray Crystallographic analysis

Supplementary Methods: X-ray Crystallographic Analysis

X-ray Crystallographic Analysis of 3a. Red platelet single crystals were grown by slow diffusion of *i*-PrOH into a solution of **3a** in CH_2Cl_2. Intensity data were collected at 133 K on a Rigaku XtaLAB AFC10 diffractometer equipped with FR-X generator, Varimax optics, and PILATUS 200K photon counting detector with Mo*K*α radiation (λ = 0.71073 Å). A total of 33678 reflections were measured with the maximum 2θ angle of 55.0°, of which 8609 were independent reflections (R_{int} = 0.0220). The crystal data are as follows: $C_{30}H_{20}$; FW = 380.46, crystal size = 0.20 × 0.10 × 0.01 mm^3, monoclinic, $P2_1$ (#4), a = 9.7527(3) Å, b = 16.7010(5) Å, c = 12.2167(3) Å, β = 101.232(3)°, V = 1951.74(10) Å^3, Z = 4, D_c = 1.295 g cm^{-3}, μ = 0.073 mm^{-1}, R_1 = 0.0331 ($I > 2\sigma(I)$), wR_2 = 0.0898 (all data), GOF = 1.045. CCDC 2184333 contains the supplementary crystallographic data for this compound. This data can be obtained free of charge from the Cambridge Crystallographic Data Centre (CCDC) at www.ccdc.cam.ac.uk/data_request.cif.

X-ray Crystallographic Analysis of 3b. Black platelet single crystals were grown by slow diffusion of *n*-hexane into a solution of **3b** in CH_2Cl_2. Intensity data were collected at 100 K on synchrotron radiation (λ = 0.4137 Å) at the BL02B1 beamline in SPring-8 (JASRI). A total of 16301 reflections were measured with the maximum 2θ angle of 31.2°, of which 3339 were independent reflections (R_{int} = 0.0877). The crystal data are as follows: $C_{48}H_{30}$; FW = 606.72, crystal size = 0.02 × 0.01 × 0.01 mm^3, triclinic, P-1 (#2), a = 3.8411(3) Å, b = 11.5144(9) Å, c = 17.6125(13) Å, α = 107.216(7)°, β = 90.470(6)°, γ = 97.393(6)°, V = 737.00(10) Å^3, Z = 1, D_c = 1.367 g cm^{-3}, μ = 0.036 mm^{-1}, R_1 = 0.0632 ($I > 2\sigma(I)$), wR_2 = 0.1397 (all data), GOF = 1.108. CCDC 2184328 contains the supplementary crystallographic data for this compound. This data can be obtained free of charge from the Cambridge Crystallographic Data Centre (CCDC) at www.ccdc.cam.ac.uk/data_request.cif.

X-ray Crystallographic Analysis of 4a. Orange platelet single crystals were grown by slow diffusion of ethyl acetate into a solution of **4a** in CH_2Cl_2. Intensity data were collected at 100 K on synchrotron radiation (λ = 0.4125 Å) at the BL02B1 beamline in SPring-8 (JASRI). A total of 92206 reflections were measured with the maximum 2θ angle of 32.8°, of which 9430 were independent reflections (R_{int} = 0.0947). The crystal data are as follows: $C_{42}H_{48}Si_2$; FW = 608.98, crystal size = 0.02

× 0.01 × 0.01 mm^3, monoclinic, $P2_1/n$ (#14), a = 12.55810(10) Å, b = 7.25340(10) Å, c = 38.5704(4) Å, β = 92.9500(10)° V = 3508.68(7) Å^3, Z = 4, D_c = 1.153 g cm^{-3}, μ = 0.043 mm^{-1}, R_1 = 0.0561 ($I > 2\sigma(I)$), wR_2 = 0.1410 (all data), GOF = 1.079. CCDC 2184330 contains the supplementary crystallographic data for this compound. This data can be obtained free of charge from the Cambridge Crystallographic Data Centre (CCDC) at www.ccdc.cam.ac.uk/data_request.cif.

X-ray Crystallographic Analysis of 4b. Yellow platelet single crystals were grown by slow diffusion of *i*-PrOH into a solution of **4b** in CH_2Cl_2. Intensity data were collected at 100 K on synchrotron radiation (λ = 0.4125 Å) at the BL02B1 beamline in SPring-8 (JASRI). A total of 56114 reflections were measured with the maximum 2θ angle of 31.1°, of which 5732 were independent reflections (R_{int} = 0.1759). The crystal data are as follows: $C_{60}H_{58}Si_2 \cdot CH_2Cl_2$; FW = 920.17, crystal size = 0.01 × 0.01 × 0.01 mm^3, orthorhombic, *Ccc*2 (#37), a = 23.5244(14) Å, b = 29.1379(16) Å, c = 7.2938(4) Å, V = 4999.5(5) Å^3, Z = 4, D_c = 1.222 g cm^{-3}, μ = 0.061 mm^{-1}, R_1 = 0.0653 ($I > 2\sigma(I)$), wR_2 = 0.1656 (all data), GOF = 1.004. CCDC 2184329 contains the supplementary crystallographic data for this compound. This data can be obtained free of charge from the Cambridge Crystallographic Data Centre (CCDC) at www.ccdc.cam.ac.uk/data_request.cif.

X-ray Crystallographic Analysis of 6a. Red block single crystals were grown by slow diffusion of *i*-PrOH into a solution of **6a** in CH_2Cl_2. Intensity data were collected at 100 K on synchrotron radiation (λ = 0.4127 Å) at the BL02B1 beamline in SPring-8 (JASRI). A total of 19869 reflections were measured with the maximum 2θ angle of 32.8°, of which 4475 were independent reflections (R_{int} = 0.0262). The crystal data are as follows: $C_{24}H_{15}Br$; FW = 383.27, crystal size = 0.10 × 0.10 × 0.05 mm^3, monoclinic, *Pn* (#7), a = 5.64839(4) Å, b = 14.07873(10) Å, c = 10.53828(8) Å, β = 90.7564(7)°, V = 837.954(11) Å^3, Z = 2, D_c = 1.519 g cm^{-3}, μ = 0.583 mm^{-1}, R_1 = 0.0232 ($I > 2\sigma(I)$), wR_2 = 0.0607 (all data), GOF = 1.070. CCDC 2184334 contains the supplementary crystallographic data for this compound. This data can be obtained free of charge from the Cambridge Crystallographic Data Centre (CCDC) at www.ccdc.cam.ac.uk/data_request.cif.

X-ray Crystallographic Analysis of 7a. Orange platelet single crystals were grown by CH_3CN vapor diffusion into a solution of **7a** in $CHCl_3$. Intensity data were collected at 100 K on synchrotron radiation (λ = 0.4131 Å) at the BL02B1 beamline in SPring-8 (JASRI). A total of 55765 reflections were measured with the maximum 2θ angle of 31.1°, of which 11440 were independent reflections (R_{int} = 0.0868). This structure contains a level-B alert regarding the Hirshfeld Test Diff for C5···C6 in the checkCif report. This alert is due to the slightly larger anisotropic temperature factor only for C6 and does not affect the validity of the structure. The crystal data are as follows: $C_{30}H_{29}SiBr$; FW = 497.53, crystal size = 0.02 × 0.02 × 0.01 mm^3, triclinic, *P*-1 (#2), a = 7.4640(4) Å, b = 12.5418(6) Å, c = 27.3333(12) Å, α = 101.774(4)°, β = 92.883(4)°, γ = 90.983(2)°, V = 2500.7(2) Å^3, Z = 4, D_c = 1.321 g cm^{-3}, μ = 0.409 mm^{-1}, R_1 = 0.0710 ($I > 2\sigma(I)$), wR_2 = 0.1634 (all data), GOF = 1.129. CCDC 2184332 contains the supplementary crystallographic data for this compound. This data can be obtained free of charge from the Cambridge Crystallographic Data Centre (CCDC) at www.ccdc.cam.ac.uk/data_request.cif.

X-ray Crystallographic Analysis of 8. Red platelet single crystals were grown by vapor diffusion of MeOH into a solution of **8** in CH_2Cl_2. Intensity data were collected at 100 K on synchrotron radiation (λ = 0.4125 Å) at the BL02B1 beamline in SPring-8 (JASRI). A total of 58341 reflections were measured with the maximum 2θ angle of 31.1°, of which 5966 were independent reflections (R_{int} = 0.0938). Four methyl groups are disordered and therefore solved using appropriate disordered models. Thus, two sets of disordered methyl groups, *i.e.* (C27, H23, H24, H25, C28, H26, H27, H28, C29, H32, H33, H34, C30, H29, H30, H31) and (C31, H41, H42, H43, C32, H44, H45, H46, C33, H38, H39, H40, C34, H35, H36, H37) were placed, and their occupancies were refined to be 0.79 and 0.21, respectively. The crystal data are as follows: $C_{30}H_{34}B_2O_4$; FW = 480.19, crystal size = 0.01 × 0.01 × 0.01 mm^3, monoclinic, $P2_1/c$ (#14), a = 17.2812(4) Å, b = 11.8256(3) Å, c = 13.0913(4) Å, β = 103.004(3)°, V = 2606.73(12) Å^3, Z = 4, D_c = 1.224 g cm^{-3}, μ = 0.034 mm^{-1}, R_1 = 0.0710 ($I > 2\sigma(I)$), wR_2 = 0.1641 (all data), GOF = 1.150. CCDC 2184331 contains the supplementary crystallographic data for this compound. This data can be obtained free of charge from the Cambridge Crystallographic Data Centre (CCDC) at www.ccdc.cam.ac.uk/data_request.cif.

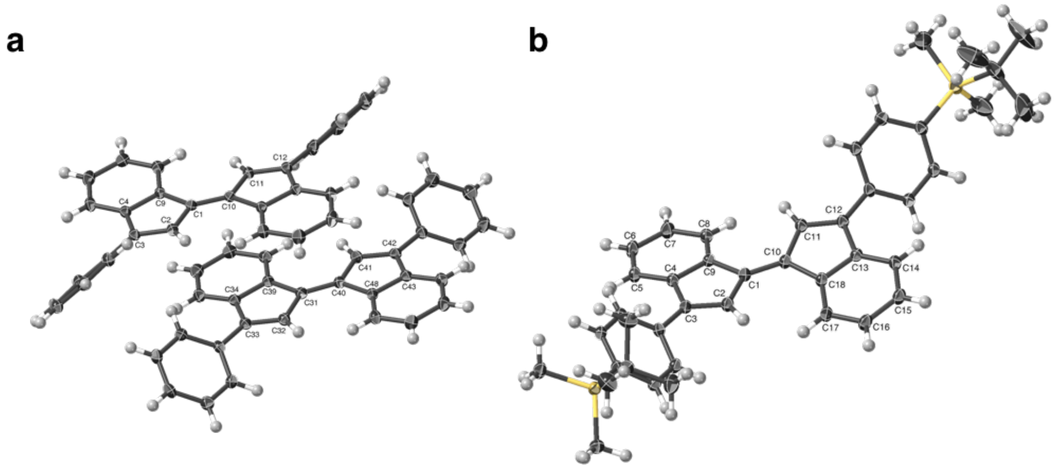

Supplementary Fig. 3: Crystal structures of biindenylidene monomers 3a and 4a. Thermal ellipsoid plots are drawn at 50% probability. Gray: carbon, white: hydrogen, yellow: silicon. **a.** Crystal structure of **3a**. The crystal lattice of **3a** consists of two crystallographically independent molecules. **b.** Crystal structure of **4a**. CCDC 2184333 and 2184330 contain the supplementary crystallographic data for **3a** and **4b**, respectively.

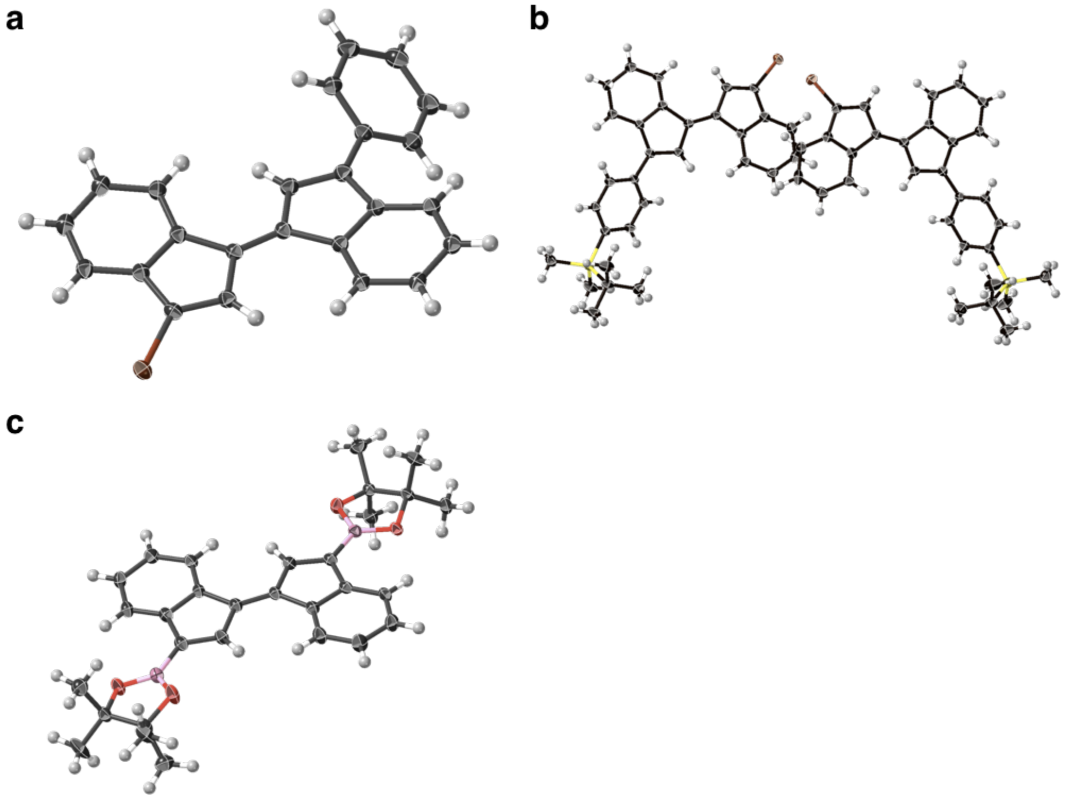

Supplementary Fig. 4: Crystal structures of biindenylidenes 6a, 7a, and 8. Thermal ellipsoid plots are drawn at 50% probability. Gray: carbon, white: hydrogen, red: oxygen, pink: boron, yellow: silicon, brown: bromine. **a.** Crystal structure of **6a**. **b.** Crystal structure of **7a**. The crystal lattice of **7a** consists of two crystallographically independent molecules. **c.** Crystal structure of **8**. CCDC 2184334, 2184332, and 2184331 contain the supplementary crystallographic data for **6a**, **7a**, and **8**, respectively.

Supplementary Table 2: Selected C–C Bond Lengths [Å] of Oligo(biindenylidene)s **3a**, **3b**, **4a**, and **4b** Determined by X-ray Crystallographic Analyses

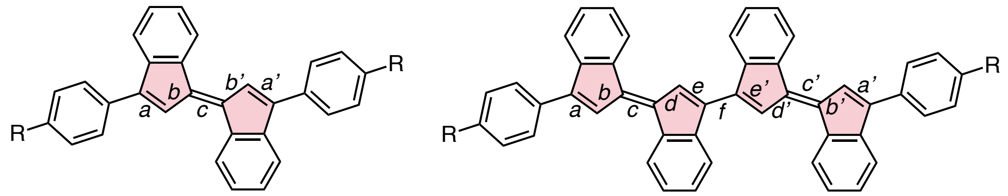

3a (R = H)
4a (R = SiMe$_2$*t*-Bu)

3b (R = H)
4b (R = SiMe$_2$*t*-Bu)

Bond	3a[a]		4a	3b	4b
	unit A	unit B			
a	C2–C3 1.355(2)<br>C11–C12 1.353(2)	C32–C33 1.356(2)<br>C41–C42 1.357(2)	C2–C3 1.362(2)<br>C11–C12 1.366(2)	1.361(3)	1.364(7)
b	C1–C2 1.457(2)<br>C10–C11 1.454(2)	C31–C32 1.456(2)<br>C40–C41 1.452(2)	C1–C2 1.450(2)<br>C10–C11 1.455(2)	1.455(2)	1.448(6)
c	C1–C10 1.363(2)	C31–C40 1.370(2)	C1–C10 1.373(2)	1.381(3)	1.381(7)
d	—	—	—	1.446(2)	1.452(7)
e	—	—	—	1.375(3)	1.364(7)
f	—	—	—	1.449(3)	1.457(10)

[a]Compound **3a** consists of two crystallographically independent units A and B in the crystal lattice.

Supplementary Table 3: Selected Bond Angles and the Sum of Them around Each Carbon Atom in Five-Membered Rings of **3a**[a]

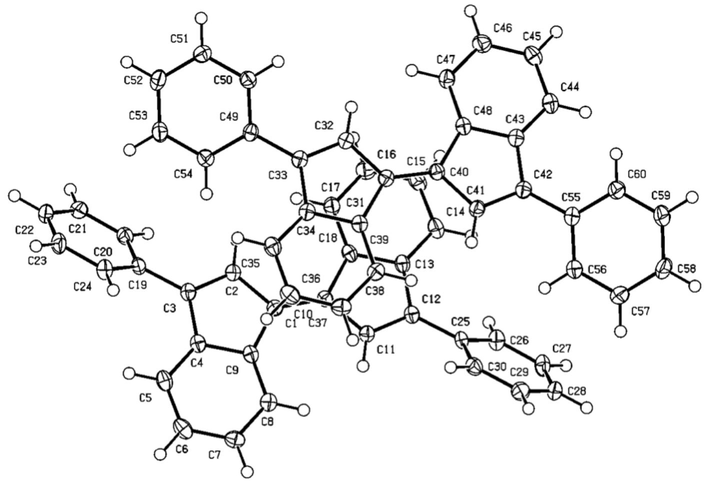

Atom	Angle 1 / deg	Angle 2 / deg	Angle 3 / deg	Sum / deg	$\theta_{\sigma\pi}$[b]
C1	C2–C1–C9 105.01(14)	C10–C1–C9 128.70(15)	C10–C1–C2 126.26(15)	359.97(25)	90.57
C3	C2–C3–C4 108.23(14)	C4–C3–C19 126.02(15)	C2–C3–C19 125.76(16)	360.01(26)	–[c]
C4	C9–C4–C3 108.31(14)	C5–C4–C3 130.33(16)	C5–C4–C9 121.08(16)	359.72(27)	91.74
C9	C4–C9–C1 107.22(15)	C8–C9–C4 118.84(15)	C8–C9–C1 133.83(15)	359.89(26)	91.08
C10	C11–C10–C18 105.28(13)	C1–C10–C11 125.55(15)	C1–C10–C18 129.15(15)	359.98(25)	90.46
C12	C11–C12–C13 108.39(15)	C11–C12–C25 125.18(16)	C13–C12–C25 126.41(15)	359.98(27)	90.47
C13	C18–C13–C12 108.07(14)	C14–C13–C18 121.26(16)	C14–C13–C12 130.55(15)	359.88(26)	91.14
C18	C13–C18–C10 107.23(15)	C17–C18–C13 118.88(16)	C17–C18–C10 133.84(15)	359.95(27)	90.73
			Average	359.92(9)	90.88(47)
C31	C32–C31–C39 104.60(14)	C40–C31–C39 128.92(15)	C40–C31–C32 126.47(15)	359.99(25)	90.33
C33	C32–C33–C34 107.93(14)	C49–C33–C34 126.11(15)	C32–C33–C49 125.96(16)	360.00(26)	90.00
C34	C39–C34–C33 108.06(14)	C35–C34–C33 130.83(16)	C35–C34–C39 121.09(16)	359.98(27)	90.46
C39	C31–C39–C34 107.78(15)	C34–C39–C38 118.81(16)	C31–C39–C38 133.41(15)	360.00(27)	90.00
C40	C41–C40–C48 104.92(14)	C31–C40–C41 126.16(15)	C31–C40–C48 128.91(15)	359.99(25)	90.33
C42	C41–C42–C43 107.90(15)	C41–C42–C55 125.01(17)	C43–C42–C45 127.03(15)	359.94(27)	90.81
C43	C42–C43–C48 108.11(14)	C44–C43–C48 120.95(17)	C42–C43–C44 130.93(16)	359.99(27)	90.33
C48	C43–C48–C40 107.47(15)	C43–C48–C47 118.92(16)	C40–C48–C47 133.60(15)	359.99(27)	90.33
			Average	359.99(9)	90.32(26)

[a] Data from X-ray crystal structure. Carbon atoms with C–H bonds are excluded because of the difficulty in identifying the geometry of carbon atoms without the assumption of the position of hydrogen atoms using AFIX program. [b] A σ–π interorbital angle estimated by use of the π orbital axis vector (POAV) analysis as shown in references S12 and S13. [c] The $\theta_{\sigma\pi}$ value could not be calculated because the sum of bond angles was slightly over 360°.

Supplementary Table 4: Selected Bond Angles and the Sum of Them around Each Carbon Atom in Five-Membered Rings of **3b**[a]

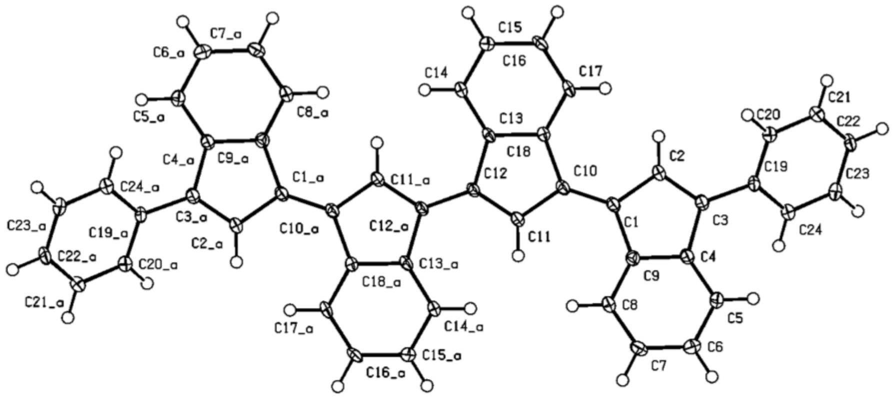

Atom	Angle 1 / deg	Angle 2 / deg	Angle 3 / deg	Sum / deg	$\theta_{\sigma\pi}$ [b]
C1	C2–C1–C9 105.35(15)	C10–C1–C9 128.36(16)	C10–C1–C2 126.28(17)	359.99(28)	90.33
C3	C2–C3–C4 108.36(15)	C4–C3–C19 126.36(16)	C2–C3–C19 125.22(17)	359.94(28)	90.81
C4	C9–C4–C3 107.59(16)	C5–C4–C3 131.27(17)	C5–C4–C9 120.97(17)	359.83(29)	91.35
C9	C4–C9–C1 107.64(15)	C8–C9–C4 119.44(17)	C8–C9–C1 132.59(17)	359.67(28)	91.88
C10	C11–C10–C18 105.39(15)	C1–C10–C11 126.21(17)	C1–C10–C18 128.34(16)	359.94(28)	90.80
C12	C11–C12–C13 106.91(15)	C11–C12–C12* 126.45(21)	C12*–C12–C13 126.63(20)	359.99(33)	90.33
C13	C18–C13–C12 107.62(15)	C14–C13–C18 119.43(16)	C14–C13–C12 132.92(16)	359.97(27)	90.57
C18	C13–C18–C10 108.12(15)	C17–C18–C13 120.51(16)	C17–C18–C10 131.11(17)	359.74(28)	91.67
			Average	359.88(10)	90.74(60)

[a] Data from X-ray crystal structure. Carbon atoms with C–H bonds are excluded because of the difficulty in identifying the geometry of carbon atoms without the assumption of the position of hydrogen atoms using AFIX program. [b] A σ–π interorbital angle estimated by use of the π orbital axis vector (POAV) analysis as shown in references S12 and S13.

Supplementary Table 5: Selected Bond Angles and the Sum of Them around Each Carbon Atom in Five-Membered Rings of **4a**[a]

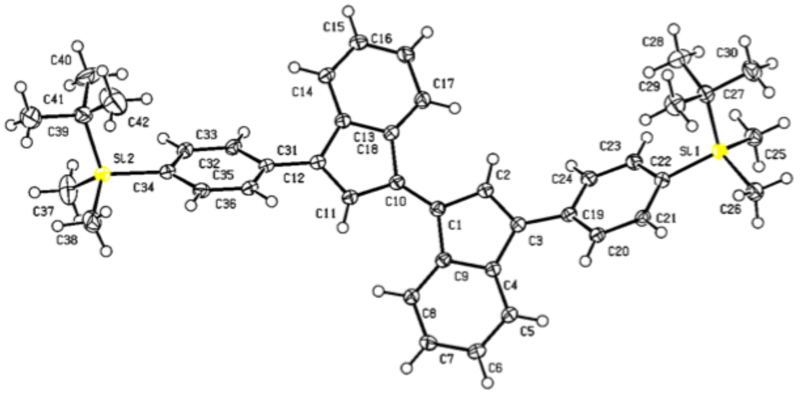

Atom	Angle 1 / deg	Angle 2 / deg	Angle 3 / deg	Sum / deg	$\theta_{\sigma\pi}$ [b]
C1	C2–C1–C9 105.37(13)	C9–C1–C10 128.84(15)	C2–C1–C10 125.63(15)	359.84(25)	91.31
C3	C2–C3–C4 108.30(14)	C4–C3–C19 126.53(14)	C2–C3–C19 122.92(15)	359.75(25)	91.64
C4	C9–C4–C3 107.81(14)	C3–C4–C5 131.29(15)	C5–C4–C9 120.72(15)	359.82(25)	91.39
C9	C4–C9–C1 107.40(14)	C4–C9–C8 120.01(15)	C1–C9–C8 132.32(15)	359.73(25)	91.70
C10	C11–C10–C18 105.66(13)	C1–C10–C11 126.45(15)	C1–C10–C18 127.87(15)	359.98(25)	90.46
C12	C11–C12–C13 108.22(14)	C11–C12–C31 125.62(15)	C13–C12–C31 126.14(14)	359.98(25)	90.47
C13	C12–C13–C18 108.22(14)	C14–C13–C18 121.03(15)	C12–C13–C14 130.66(15)	359.91(25)	90.98
C18	C10–C18–C18 107.23(14)	C13–C18–C17 119.37(15)	C10–C18–C17 133.39(15)	359.99(25)	90.33
			Average	359.88(9)	91.03(56)

[a] Data from X-ray crystal structure. Carbon atoms with C–H bonds are excluded because of the difficulty in identifying the geometry of carbon atoms without the assumption of the position of hydrogen atoms using AFIX program. [b] A σ–π interorbital angle estimated by use of the π orbital axis vector (POAV) analysis as shown in references S12 and S13.

Supplementary Table 6: Selected Bond Angles and the Sum of Them around Each Carbon Atom in Five-Membered Rings of **4b**[a]

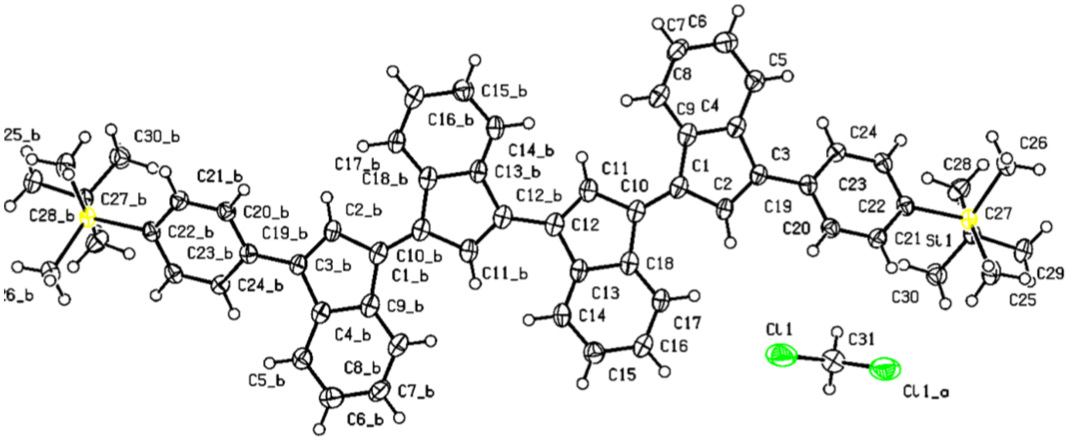

Atom	Angle 1 / deg	Angle 2 / deg	Angle 3 / deg	Sum / deg	$\theta_{\sigma\pi}$ [b]
C1	C2–C1–C9 105.66(42)	C9–C1–C10 128.25(45)	C2–C1–C10 126.09(47)	360.00(77)	90.00
C3	C2–C3–C4 108.11(42)	C4–C3–C19 127.17(44)	C2–C3–C19 124.72(44)	360.00(75)	90.00
C4	C9–C4–C3 107.23(43)	C5–C4–C3 132.30(45)	C5–C4–C9 120.26(45)	359.79(77)	91.50
C9	C4–C9–C1 107.83(41)	C8–C9–C4 118.95(50)	C1–C9–C8 133.02(49)	359.80(81)	91.46
C10	C11–C10–C18 105.76(42)	C1–C10–C11 126.16(47)	C1–C10–C18 128.08(43)	360.00(76)	90.00
C12	C11–C12–C13 108.51(42)	C11–C12–C12* 126.05(58)	C12*–C12–C13 125.42(58)	359.98(92)	90.47
C13	C12–C13–C18 107.69(45)	C14–C13–C18 120.04(47)	C12–C13–C14 132.12(46)	359.85(80)	91.27
C18	C10–C18–C13 107.14(42)	C13–C18–C17 119.33(48)	C10–C18–C17 133.20(45)	359.67(78)	91.87
			Average	359.89(28)	90.82(79)

[a] Data from X-ray crystal structure. Carbon atoms with C–H bonds are excluded because of the difficulty in identifying the geometry of carbon atoms without the assumption of the position of hydrogen atoms using AFIX program. [b] A σ–π interorbital angle estimated by use of the π orbital axis vector (POAV) analysis as shown in references S12 and S13.

5. Electrochemical Properties

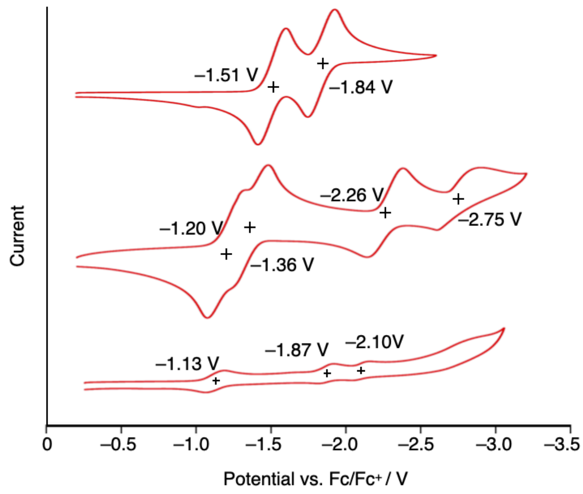

Supplementary Fig. 5: Electrochemical properties of oligo(biindenylidene)s 3a–c. Cyclic voltammograms were recorded at a scan rate of 0.1 V s^{-1} in tetrahydrofuran using [*n*-Bu_4N][PF_6] (0.1 M) as the supporting electrolyte. All potentials are referenced against the ferrocene/ferrocenium (Fc/Fc^+) couple.

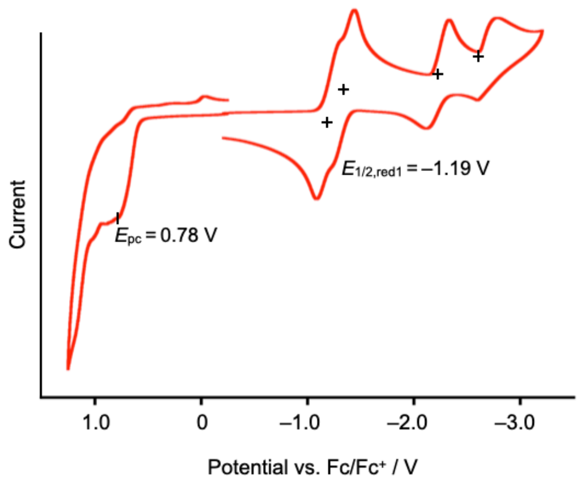

Supplementary Fig. 6: Electrochemical properties of oligo(biindenylidene)s 4b. Cyclic voltammograms were recorded at a scan rate of 0.1 V s^{-1} in THF and CH_2Cl_2 using [*n*-Bu_4N][PF_6] (0.1 M) as the supporting electrolyte. All potentials are referenced against the Fc/Fc^+ couple.

Supplementary Table 7: Electrochemical Data for Oligo(biindenylidene)s **3a–c** and **4a–c**, and C_{60} in THF

Cmpd	$E_{1/2,\text{red1}}$ / V[a,b] (E_{pc1} / V[a,c])	$E_{1/2,\text{red2}}$ / V[a,b] (E_{pc2} / V[a,c])	$E_{1/2,\text{red3}}$ / V[a,b] (E_{pc3} / V[a,c])	$E_{1/2,\text{red4}}$ / V[a,b] (E_{pc4} / V[a,c])	$E_{1/2,\text{red5}}$ / V[a,b] (E_{pc5} / V[a,c])	LUMO / eV[d]
**3a**	–1.51 (–1.60)	–1.84 (–1.93)	n.d.[e]	n.d.[e]	n.d.[e]	–3.68
**3b**	–1.20 (–1.32)	–1.36 (–1.48)	–2.26 (–2.38)	–2.75 (–2.90)	n.d.[e]	–3.97
**3c**	–1.13 (–1.19)	–1.87 (–1.92)	–2.10 (–2.16)	–2.59[f] (–2.71)	n.d.[e]	–4.07
**4a**	–1.48 (–1.57)	–1.78 (–1.87)	n.d.[e]	n.d.[e]	n.d.[e]	–3.70
**4b**	–1.20 (–1.31)	–1.33 (–1.44)	–2.22 (–2.33)	–2.60 (–2.79)	n.d.[e]	–3.97
**4c**	–1.09 (–1.15)	–1.82 (–1.87)	–2.03 (–2.07)	–2.61 (–2.67)	–2.74 (–2.90)	–4.09
C_{60}	–0.89 (–0.94)	–1.47 (–1.50)	–2.03 (–2.07)	–2.50 (–2.55)	n.d.[e]	–4.28

[a] The redox potential determined by cyclic voltammetry under the following conditions: sample (1 mM) and $[Bu_4N][PF_6]$ (0.1 M) in THF; scan rate 0.1 V s^{-1}. The potential was calibrated relative to the ferrocene/ferrocenium (Fc/Fc^+) couple. [b] Half-wave redox potential. [c] Peak cathodic potential. [d] Energy level based on the Fermi scale estimated using the values of the onset potentials of the cyclic voltammograms using the following equation, $E_{\text{LUMO}}/\text{ eV} = -(E_{\text{onset,red}} + 5.1)$, described in ref. S14. [e] Not detected. [f] The value of half-peak potential $E_{\text{pc/2}}$, i.e., the potential at half the maximum current in the cyclic voltammogram as a way to approximately estimate $E_{1/2}$. This $E_{\text{pc/2}}$ value is shown because of the uncertainty of the $E_{1/2}$ value due to the masking by the high baseline currents caused by the reduction of solvents. The $E_{\text{pc/2}}$ value is calculated based on the method described in ref. S15.

Supplementary Discussion 3: Evaluation of the first reductive wave of 4c. In the cyclic voltammogram of **4c**, the i_p value for the first redox wave of 7.23 × 10^{-6} A was found to be substantially larger than that for the second redox wave of 3.83 × 10^{-6} A (Fig. 4a in the manuscript). Therefore, we tried to characterize the number of electrons transferred in the first redox process. According to the Randles–Sevcik equation (eq. 1), the peak current i_p (A) in the cyclic voltammogram correlates with the number of electrons transferred in the redox process n, where υ, A, D_o, and C^0 represent the scan rate (V s^{-1}), the electrode surface area (cm^2), the diffusion coefficient of the reduced analyte (cm^2 s^{-1}), and the concentration of the analyte (mol cm^{-3}), respectively.

$$i_p = 0.446nFAC^0\left(\frac{nF\upsilon D_o}{RT}\right)^{1/2} \qquad (1)$$

The scan rate υ of 0.1 V s^{-1} is constant over the measurements. Electrode surface area A and the analyte concentration used in these measurements were 7.07 × 10^{-2} cm^2 and 1.0 × 10^{-6} mol cm^{-3}, respectively. Based on the hypothesis that the second and subsequent waves are one-electron redox processes (n = 1),

the D_o value for a single-electron process (n = 1) at T = 298.15 K was calculated to be 4.07 × 10^{-7} cm^2 s^{-1} using the i_p value of 3.83 × 10^{-6} A for the second redox wave.

Assuming that the analyte is freely diffusing without adsorption on the electrode and that the redox reactions take place only in the small area close to the electrodes, the values of A, D_o, and C^0 can be regarded to be constant over the whole reversible redox process. By using eq. 1, the i_p value for the two-electron redox process (n = 2) is estimated to be 5.49 × 10^{-6} A, which is comparable to the experimentally observed value for the first redox wave of 7.23 × 10^{-6} A. Therefore, we concluded that this first redox wave is characterized as two-electron process.

6. Photophysical Properties

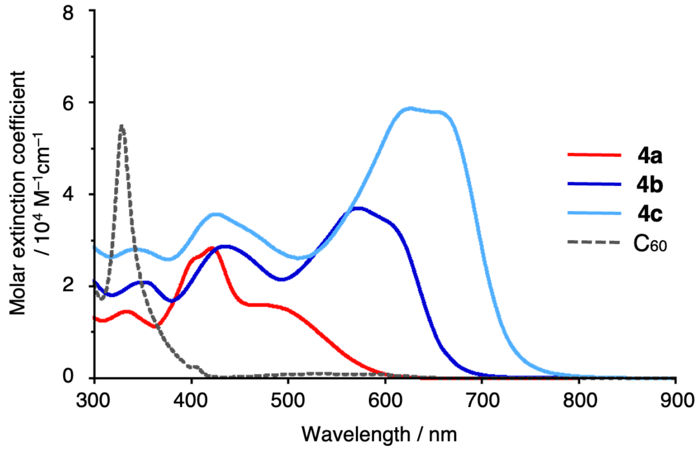

Supplementary Fig. 7: Photophysical properties of oligo(biindenylidene)s 4a–c compared with that of C_{60}. UV/Vis/NIR electronic absorption spectra of **4a** (red solid line), **4b** (blue solid line), **4c** (light-blue solid line), and C_{60} (black broken line) in CH_2Cl_2.

Supplementary Table 8: Summary of Photophysical Data of **3a**–**c** and **4a**–**c**[a]

Cmpd	λ_{abs} / nm[b]	E_{abs} / eV[c]	ε / M^{-1} cm^{-1} [d]
**3a**	482	2.57	1.32 × 10^4
**3b**	607	2.04	2.93 × 10^4
**3c**	653	1.90	5.34 × 10^4
**4a**	492	2.52	1.54 × 10^4
**4b**	612	2.03	3.23 × 10^4
**4c**	660	1.88	5.76 × 10^4

[a]In CH_2Cl_2. [b]Absorption maximum wavelength of the longest wavelength absorption band. [c]Absorption energy at an absorption maximum. [d]Molar extinction coefficient at the absorption maximum wavelength of the longest wavelength absorption band.

7. Quantum Chemical Calculations

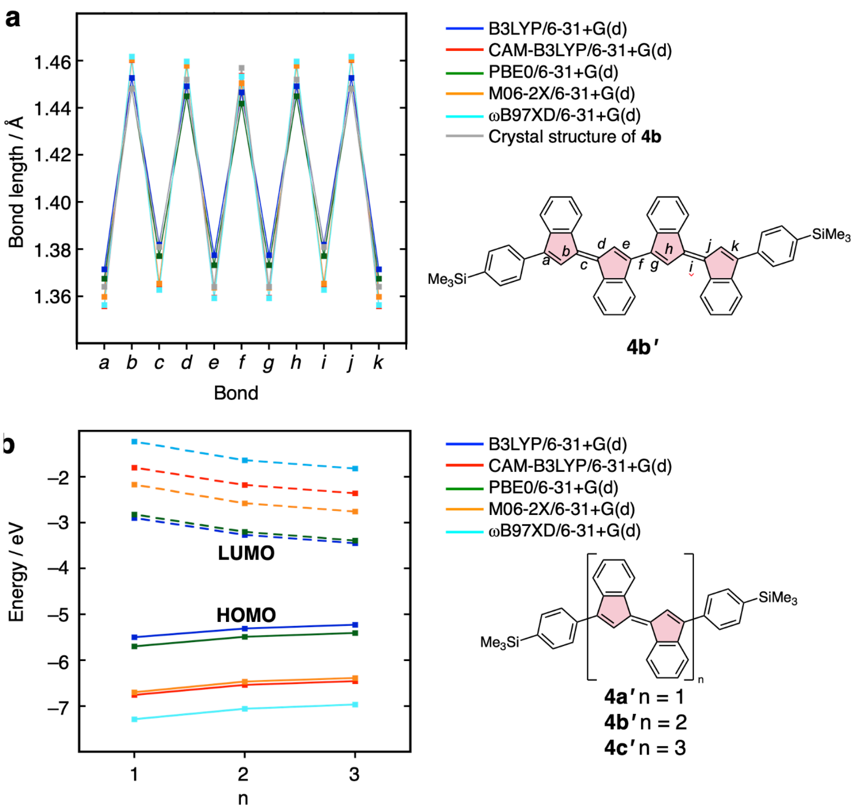

Supplementary Fig. 8: Benchmark results of DFT calculations for oligo(biindenylidenes) 4a′–c′ using various density functionals. a. Selected C–C bond lengths. **b.** Orbital energy levels of Kohn-Sham HOMOs and LUMOs.

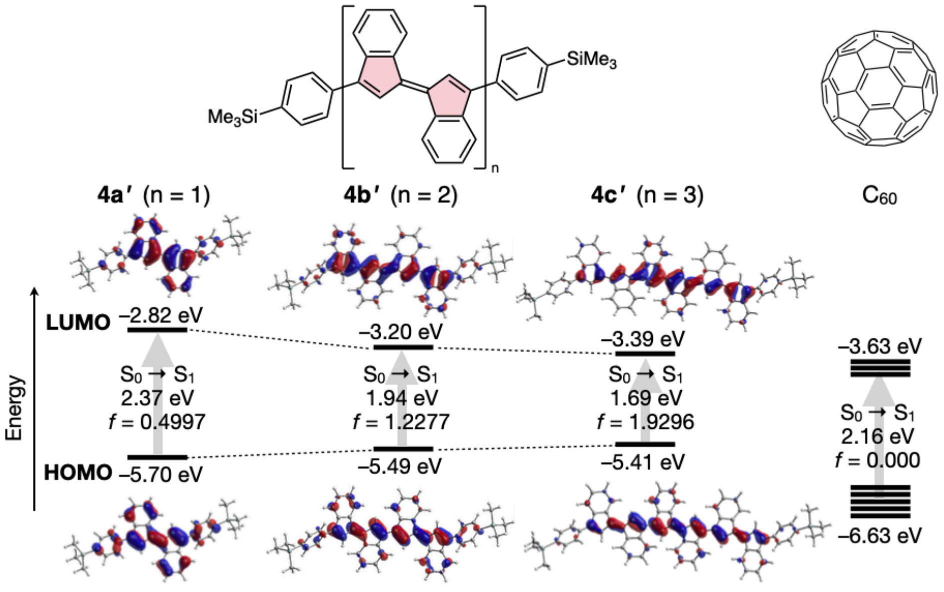

Supplementary Fig. 9: Comparison of the electronic structures of oligo(biindenylidene)s 4a′–c′ with that of C_{60}. Energy diagrams of the Kohn-sham frontier molecular orbitals of **4a′**, **4b′**, **4c′**, and C_{60}, and the corresponding pictorial representations and the TD-DFT vertical excitations for the lowest-energy transitions calculated at the PBE0/6-31+G(d) level of theory.

Supplementary Table 9: Selected Bond Lengths [Å] in the Optimized Geometries of Charge-Neutral Biindenylidene Monomers **3a** and **4a′** together with the Corresponding Experimental Data

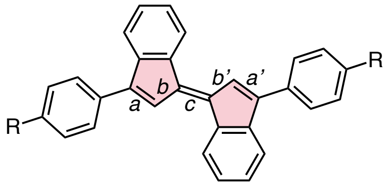

3a (R = H)
4a (R = SiMe$_2$*t*-Bu)
4a' (R = SiMe$_3$)

Bond	3a			4a′	4a
	Calc.[a]	Exp.[b,c]		Calc.[a]	Exp.[b]
		unit A	unit B		
a	C11–C12 1.36434	C2–C3 1.355(2)	C32–C33 1.356(2)	C15–C18 1.36636	C2–C3 1.362(2)
	C25–C26 1.36565	C11–C12 1.353(2)	C41–C42 1.357(2)	C3–C21 1.36636	C11–C12 1.366(2)
b	C12–C14 1.45085	C1–C2 1.457(2)	C31–C32 1.456(2)	C14–C18 1.45004	C1–C2 1.450(2)
	C26–C28 1.45081	C10–C11 1.454(2)	C40–C41 1.452(2)	C6–C21 1.45004	C10–C11 1.455(2)
c	C14–C28 1.37465	C1–C10 1.363(2)	C31–C40 1.370(2)	C6–C14 1.37459	C1–C10 1.373(2)

[a]Computational results by geometry optimization calculated at the PBE0/6-31+G(d) level of theory. [b]Experimental values determined by X-ray crystallographic analyses. [c]Compound **3a** consists of two crystallographically independent units A and B in the crystal lattice.

Supplementary Table 10: Selected Bond Lengths [Å] in the Optimized Geometries of Charge-Neutral Biindenylidene Dimers **3b** and **4b′** together with the Corresponding Experimental Data

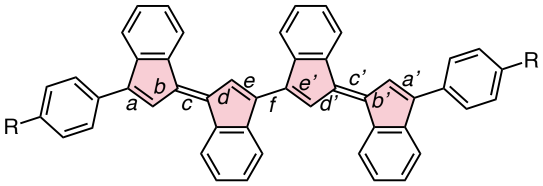

3b (R = H)
4b (R = SiMe$_2$*t*-Bu)
4b' (R = SiMe$_3$)

Bond	3b		4b′	4b
	Calc.[a]	Exp.[b]	Calc.[a]	Exp.[b]
a	C11–C12 1.36696	1.361(3)	C11–C12 1.36746	1.364(7)
	C53–C54 1.36696		C44–C46 1.36746	
b	C12–C14 1.44850	1.455(2)	C12–C14 1.44814	1.448(6)
	C54–C56 1.44850		C43–C44 1.44814	
c	C14–C28 1.37687	1.381(3)	C14–C15 1.37703	1.381(7)
	C42–C56 1.37687		C42–C43 1.37703	
d	C26–C28 1.44518	1.446(2)	C15–C16 1.44488	1.452(7)
	C40–C42 1.44518		C40–C42 1.44488	
e	C25–C26 1.37292	1.375(3)	C16–C18 1.37320	1.364(7)
	C39–C40 1.37292		C39–C40 1.37320	
f	C25–C39 1.44211	1.449(3)	C18–C39 1.44185	1.457(10)

[a]Computational results by geometry optimization calculated at the PBE0/6-31+G(d) level of theory. [b]Experimental values determined by X-ray crystallographic analyses.

Supplementary Table 11: Selected Bond Lengths [Å] in the Optimized Geometries of Charge-Neutral

Biindenylidene Trimers **3c** and **4c′**[a]

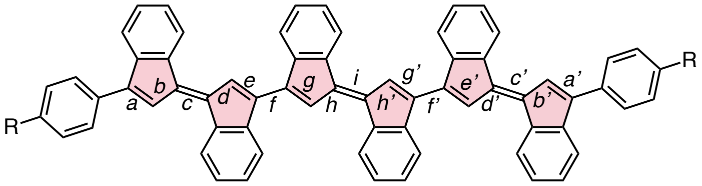

3b (R = H)
4b' (R = $SiMe_3$)

Bond	3c	4c′
a	C39–C40 1.36554	C44–C46 1.36768
	C81–C82 1.36546	C67–C68 1.36743
b	C40–C42 1.44850	C43–C44 1.44770
	C82–C84 1.44875	C68–C70 1.44802
c	C42–C56 1.37751	C42–C43 1.37741
	C70–C84 1.37725	C70–C71 1.37710
d	C54–C56 1.44484	C40–C42 1.44422
	C68–C70 1.44534	C71–C72 1.44492
e	C53–C54 1.37331	C39–C40 1.37392
	C67–C68 1.37285	C72–C74 1.37325
f	C11–C53 1.44096	C18–C39 1.44067
	C25–C67 1.44260	C11–C74 1.44250
g	C11–C12 1.37408	C16–C18 1.37440
	C25–C26 1.37277	C11–C12 1.37296
h	C12–C14 1.44297	C15–C16 1.44266
	C26–C28 1.44272	C12–C14 1.44247
i	C14–C28 1.38021	C14–C15 1.38033

[a]Computational results by geometry optimization calculated at the PBE0/6-31+G(d) level of theory.

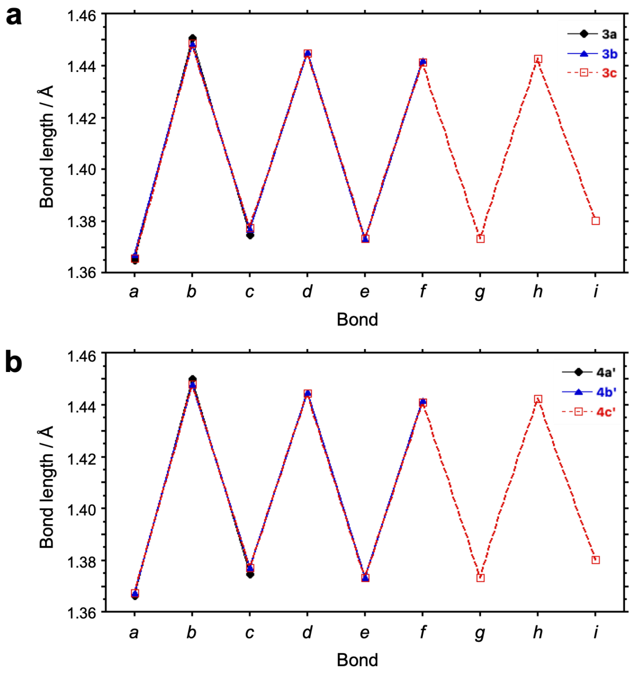

Supplementary Fig. 10: Plot of selected bond lengths in the optimized geometries of charge-neutral oligo(biindenylidene)s 3a–c and 4a′–4c′. a. Bond lengths of **3a–c**. **b.** Bond lengths of **4a′–c′**. The calculations were conducted at the PBE0/6-31+G(d) level of theory. The bond numbers *a*–*i* correspond to those illustrated in Supplementary Tables 9–11.

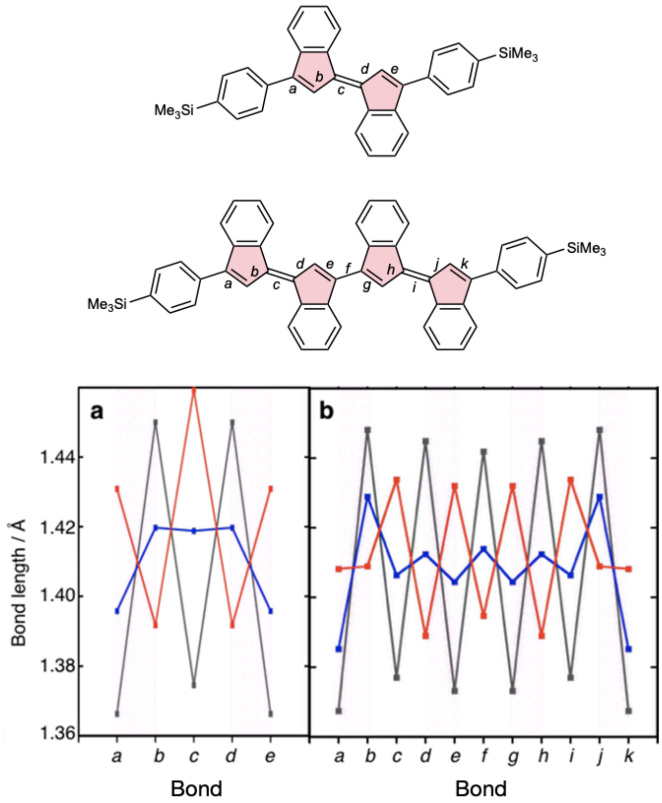

Supplementary Fig. 11: Plot of selected bond lengths in the optimized geometries of charge-neutral oligo(biindenylidene)s 4a′ and 4b′, and the corresponding radical anions and dianions. The charge-neutral oligo(biindenylidene)s **4a′** and **4b′** (gray), radical anions [**4a′**]$^{\bullet-}$ and [**4b′**]$^{\bullet-}$ (blue), and dianions [**4a′**]$^{2-}$ and [**4b′**]$^{2-}$ (red) calculated at the (U)PBE0/6-31+G(d) level of theory. **a.** Plot for biindenylidene monomers **4a′**, [**4a′**]$^{\bullet-}$, and [**4a′**]$^{2-}$. **b.** Plot for biindenylidene dimers **4b′**, [**4b′**]$^{\bullet-}$, and [**4b′**]$^{2-}$.

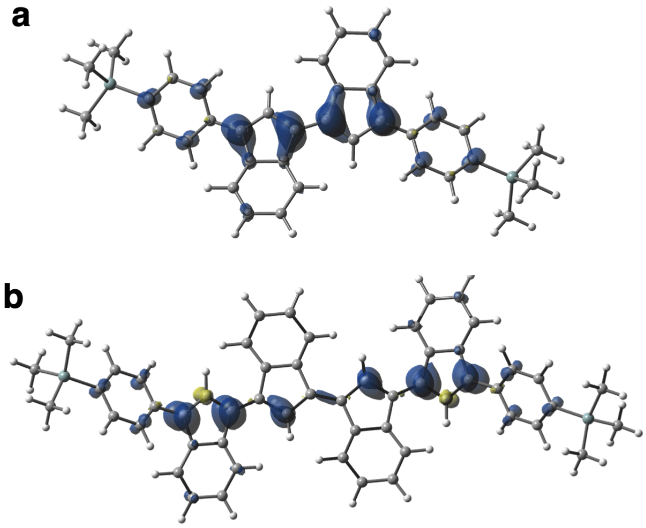

Supplementary Fig. 12: Pictorial representation of the Mulliken spin densities of oligo(biindenylidene) radical anions [4a′]$^{\bullet-}$ and [4b′]$^{\bullet-}$. a. Spin density distribution of [**4a′**]$^{\bullet-}$. **b.** Spin density distribution of [**4b′**]$^{\bullet-}$. The calculations were conducted at the UPBE0/6-31+G(d) level of theory (isovalue = 0.003).

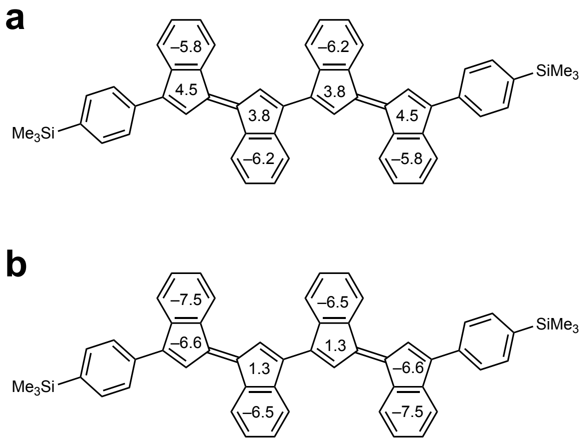

Supplementary Fig. 13: The NICS(0) values of 4b′ and [4b′]$^{2–}$. a. The NICS(0) values for charge-neutral biindenylidene dimer **4b′**. **b.** The NICS(0) values for dianionic biindenylidene dimer [**4b′**]$^{2–}$. The calculations were performed at the PBE0/6-31+G(d)level of theory.

Supplementary Table 12: Selected Data of the TD-DFT Vertical Excitation of Oligo(biindenylidene)s **3** and **4′**[a]

cmpd	state	transition energy / eV (wavelength / nm)	oscillator strength *f*	main CI coefficient
**3a**	$S_0 \rightarrow S_1$	2.4143 (513)	0.3650	0.63280 (HOMO→LUMO)<br>–0.31437 (HOMO–2→LUMO)
**3b**	$S_0 \rightarrow S_1$	1.9648 (631)	1.0981	0.69173 (HOMO→LUMO)<br>–0.13457 (HOMO–4→LUMO)
**3c**	$S_0 \rightarrow S_1$	1.7175 (722)	1.7669	0.69926 (HOMO→LUMO)
**4a′**	$S_0 \rightarrow S_1$	2.3732 (522)	0.4997	0.64407 (HOMO→LUMO)<br>0.29142 (HOMO–2→LUMO)
**4b′**	$S_0 \rightarrow S_1$	1.9390 (639)	1.2277	0.69272 (HOMO→LUMO)<br>–0.12815 (HOMO–4→LUMO)
**4c′**	$S_0 \rightarrow S_1$	1.6929 (732)	1.9296	0.69931 (HOMO→LUMO)

[a]Calculated at the PBE0/6-31+G(d) level of theory.

Supplementary Discussion 4: Assignments of the Electronic Absorption of 3c. The TD-DFT vertical excitations of **3c** calculated at the TD-(U)PBE0/6-31+G(d) level were shown together with the electronic absorption spectrum in Supplementary Fig. 14. Accordingly, except for the $S_0 \rightarrow S_1$ (722 nm, f = 1.7669) and $S_0 \rightarrow S_7$ transitions (479 nm, f = 0.12500), the $S_0 \rightarrow S_n$ transitions up to n = 10 are found to be forbidden transitions with oscillator strength f less than 0.1. Therefore, the broad and intense first absorption band consisting of two peaks at 620 nm and 653 nm is considered to be a superimposition of the $S_0 \rightarrow S_1$ transitions of the various conformers of **3c**.

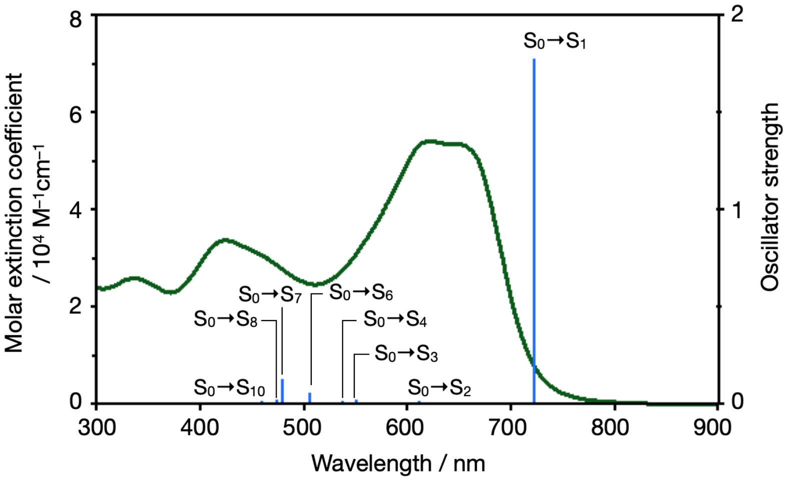

Supplementary Fig. 14: Comparison of the experimental electronic absorption of 3c with the calculated TD-DFT vertical excitations. UV/Vis/NIR electronic absorption spectrum of **3c** in CH_2Cl_2 (green line) and the corresponding TD-DFT vertical excitations (blue line) calculated at the TD-PBE0/6-31+G(d) level of theory.

Supplementary Discussion 5: Insights into the Structural Changes of Oligo(biindenylidene)s Upon Reduction. The selected C–C bond lengths of the optimized geometries of charge-neutral biindenylidene monomers and dimers **4a′** and **4b′**, and the corresponding radical anions [**4a′**]$^{\bullet-}$ and [**4b′**]$^{\bullet-}$, and dianions [**4a′**]$^{2-}$ and [**4b′**]$^{2-}$ were summarized in Supplementary Fig. 11. Those for **4b** and [**4b′**]$^{2-}$ were also shown in Fig. 4b.

The optimized structures of the radical anions [**4a′**]$^{\bullet-}$ and [**4b′**]$^{\bullet-}$ adopted highly coplanar geometries with significantly smaller bond length alternation (BLA) along the main chain (Supplementary Fig. 11), indicative of the delocalization of spin and charge densities along the main chain. The spatial distribution of the Mulliken spin densities of [**4a′**]$^{\bullet-}$ and [**4b′**]$^{\bullet-}$ supports the delocalization of spin densities (Supplementary Fig. 12). Given that these optimized structures of these radical anions [**4a′**]$^{\bullet-}$ and [**4b′**]$^{\bullet-}$ differ significantly from those of the charge-neutral species **4a′** and **4b′**, the potential difference between the one- and two-electron reduction of oligo(biindenylidene)s should be reasonably large. Indeed, the energy split between the first and second redox waves of the monomer **4a** was 0.31 V in cyclic voltammetry (Fig. 4a). On the other hand, for dimer **4b**, the splitting between the first and second redox waves is as small as 0.15 V (Fig. 4a), which is inconsistent with the above working hypothesis based on the optimized geometry of [**4b′**]$^{\bullet-}$. This discrepancy may be attributable to the presence of a conformer of **4b** in solution with varied dihedral angles around the central C–C bond (bond *f*). The broad first absorption band observed in the electronic absorption spectrum (Fig. 5a and Supplementary Fig. 7) strongly suggests that **4b** is a mixture of various conformers in solution at an ambient temperature. The reduction of the mixture of several conformers of **4b** might produce several kinetically favorable conformers of [**4b′**]$^{\bullet-}$ with distinct structures from the optimized geometry. Similarly, we encountered similar issues in the DFT calculations for radical anionic trimer [**4c′**]$^{\bullet-}$ and dianionic trimer [**4c′**]$^{2-}$ most likely due to many possible conformers. Given the difficulty in obtaining computational results that provide a reasonable explanation for experimental results at this stage, we have refrained from the

discussion about radical anions of oligo(biindenylidene)s (**3**$^{\bullet-}$ and **4**$^{\bullet-}$) and the reduced species of trimers (**3c** and **4c**) in this manuscript based on the computational results. More in-depth investigations by both experiments and theoretical calculations will be reported elsewhere.

8. Time-Resolved Microwave Conductivity Measurements

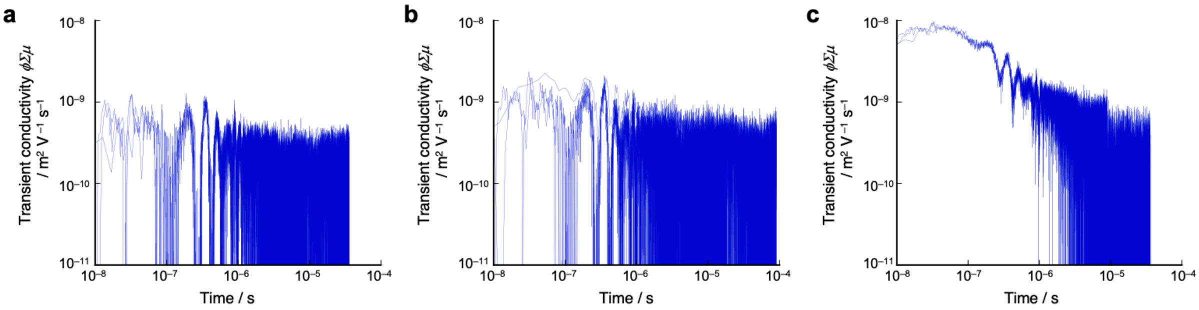

Supplementary Fig. 15: **Flash-Photolysis TRMC transients recorded for polycrystalline 3a–c**. **a**, **b**, and **c** shows the flash-photolysis TRMC transients for **3a**, **3b**, and **3c**, respectively, under excitation at 355 nm, 4.6×10^{15} photons cm^{-2}. End-of-pulse conductivity is almost consistent for **3a** and **3b**, jumping up for **3c**. Crystallites of corresponding compounds were sealed with Cytop® overcoating under vacuo, and all the transients were recorded at dry N_2 atomosphere.

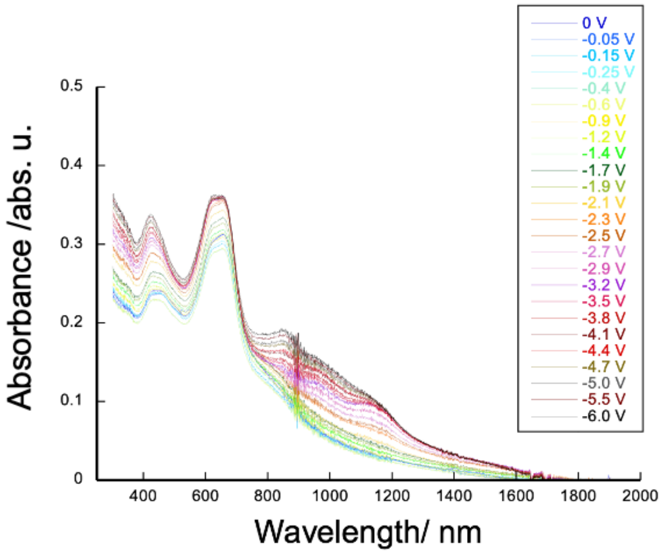

Supplementary Fig. 16: Electronic absorption spectra of 3c. The spectra in CH_2Cl_2 at 80 μmol/dm^3 conc. in its neutral and upon electrochemical reduction. The spectra were recorded under electrochemical reduction with 0.10 mol dm^{-3} [n-Bu_4N][BF_4] at 298 K.

9. Appendix

9.1 Cartesian Coordinates of the Optimized Geometries

Supplementary Table 13: Cartesian Coordinates (Å) of the Optimized Geometry for **3a** Calculated at the PBE0/6-31+G(d) Level of Theory

H	4.42396236	2.64840890	0.10263268
C	3.35295675	2.69744025	-0.07338303
C	0.57416067	2.84529172	-0.50884062
C	2.58073307	1.54286846	-0.10568761
C	2.72920306	3.93402699	-0.26558944
C	1.35752106	4.00441577	-0.49673503
C	1.17622288	1.61256527	-0.28032057
H	3.32215755	4.84488221	-0.24435807
H	0.88611807	4.96876205	-0.66766445
H	-0.48744041	2.93007239	-0.71295966
C	2.95315444	0.12798133	0.04409005
C	1.81535784	-0.61883078	-0.05134146
H	1.78167110	-1.69440662	0.05362971
C	0.65329032	0.23071451	-0.23258848
H	-4.43342484	-2.61072364	-0.41077813
C	-3.35025530	-2.65600946	-0.48527924
C	-0.55423934	-2.76123825	-0.81036885
C	-2.57404479	-1.51879825	-0.29897239
C	-2.71806255	-3.86081349	-0.80746878
C	-1.33577887	-3.91056631	-0.97146409
C	-1.16576061	-1.56659307	-0.44476968
H	-3.31303076	-4.75894556	-0.95269531
H	-0.85678566	-4.84697404	-1.24558653
H	0.51118704	-2.81849419	-1.00477588
C	-2.95936339	-0.12361825	-0.04196877
C	-1.82870230	0.64114666	-0.08368651
H	-1.80090934	1.69502835	0.15723339
C	-0.65501922	-0.18821929	-0.28233247
C	-4.31246403	0.36235113	0.23683261
C	-6.88368678	1.34649298	0.81118325
C	-5.19272801	-0.36167388	1.05658860
C	-4.74905338	1.58777573	-0.29094205
C	-6.02040405	2.07475328	-0.00682575
C	-6.46318756	0.12854476	1.34332418
H	-4.86326100	-1.29759072	1.49994709
H	-4.08718718	2.14599479	-0.94871648
H	-6.34218711	3.02217151	-0.43216847
H	-7.12598878	-0.44092658	1.99025427
H	-7.87838236	1.72543242	1.03151111
C	4.30374070	-0.39403391	0.27463551
C	6.87613325	-1.45427931	0.68546833
C	4.51488166	-1.43593950	1.19108382
C	5.40870574	0.10690973	-0.43134119
C	6.68051240	-0.42148772	-0.23002840
C	5.78724051	-1.96012410	1.39452884
H	3.67197047	-1.81735211	1.76233649
H	5.26185939	0.89024708	-1.17010976
H	7.52175420	-0.02831270	-0.79562493
H	5.93068480	-2.76135673	2.11532617
H	7.87084773	-1.86223496	0.84576211

Supplementary Table 14: Cartesian Coordinates (Å) of the Optimized Geometry for **3b** Calculated at the PBE0/6-31+G(d) Level of Theory

H	-7.72737428	-2.59231363	1.12163461
C	-6.71715570	-2.64895283	0.72535015
C	-4.14890282	-2.80880883	-0.42115273
C	-5.97948129	-1.49698754	0.48304047
C	-6.15196530	-3.89101446	0.41864726
C	-4.88298496	-3.96880320	-0.15009615
C	-4.68210454	-1.57037757	-0.08138794
H	-6.71670056	-4.80041414	0.60762681
H	-4.46170712	-4.93838262	-0.40268423
H	-3.18753169	-2.89634386	-0.91600868
C	-6.33420288	-0.07736309	0.62686698
C	-5.30637678	0.66774035	0.11995243
H	-5.25305290	1.74698356	0.17280001
C	-4.21559876	-0.18644478	-0.30280290
H	0.54069517	2.65159027	-2.11646101
C	-0.52335229	2.68250109	-1.89900360
C	-3.30783383	2.73057991	-1.47770446
C	-1.18044281	1.56619837	-1.39766457
C	-1.26126039	3.84036143	-2.16178422
C	-2.63898531	3.86207283	-1.95534572
C	-2.57745783	1.58682417	-1.17178862
H	-0.75880561	4.72215875	-2.55123712
H	-3.20590257	4.76077481	-2.18417874
H	-4.38806107	2.75564290	-1.38172720
C	-0.69012351	0.20887634	-1.10706784
C	-1.77389419	-0.56471139	-0.77254941
H	-1.70844764	-1.58307884	-0.41559767
C	-2.97847031	0.23300409	-0.73793765
H	-0.54070104	-2.65162913	-2.11644791
C	0.52335177	-2.68253910	-1.89901659
C	3.30784583	-2.73061137	-1.47780591
C	1.18045356	-1.56623810	-1.39768769
C	1.26125477	-3.84039504	-2.16182778
C	2.63898634	-3.86210263	-1.95543533
C	2.57747515	-1.58686226	-1.17184783
H	0.75879101	-4.72218996	-2.55127454
H	3.20589890	-4.76080032	-2.18429690
H	4.38807559	-2.75567082	-1.38186962
C	0.69014121	-0.20891556	-1.10708278
C	1.77392130	0.56467702	-0.77260762
H	1.70848419	1.58304995	-0.41566875
C	2.97849192	-0.23304552	-0.73798269
H	7.72734930	2.59235312	1.12158490
C	6.71713623	2.64896996	0.72528302
C	4.14889821	2.80876855	-0.42126274
C	5.97947801	1.49699019	0.48299245
C	6.15193683	3.89101712	0.41853807
C	4.88296355	3.96877800	-0.15022564
C	4.68210940	1.57035282	-0.08145714
H	6.71665960	4.80042774	0.60750228
H	4.46167923	4.93834669	-0.40284406
H	3.18753194	2.89627680	-0.91613356
C	6.33420987	0.07737311	0.62686150
C	5.30639667	-0.66775276	0.11995240

H	5.25307743	-1.74699290	0.17284169
C	4.21561767	0.18641214	-0.30284509
C	7.55892002	-0.45175449	1.22933599
C	9.87725809	-1.52561222	2.40469199
C	8.11950637	0.13178516	2.37681960
C	8.18627287	-1.58261104	0.68248885
C	9.33258944	-2.11373442	1.26346707
C	9.26416386	-0.40337006	2.95977406
H	7.63165045	0.98829784	2.83431420
H	7.77796569	-2.02869225	-0.22115433
H	9.80790692	-2.98492702	0.81949398
H	9.67602066	0.05506739	3.85546839
H	10.77461157	-1.93895977	2.85793215
C	-7.55892187	0.45179254	1.22929950
C	-9.87727587	1.52570684	2.40457172
C	-8.18625991	1.58263011	0.68239642
C	-8.11953018	-0.13169868	2.37679701
C	-9.26419571	0.40348462	2.95970985
C	-9.33258478	2.11378126	1.26333303
H	-7.77793436	2.02867376	-0.22125698
H	-7.63168512	-0.98819430	2.83433497
H	-9.67606987	-0.05491472	3.85541571
H	-9.80789089	2.98495809	0.81931681
H	-10.77463549	1.93907621	2.85777977

Supplementary Table 15: Cartesian Coordinates (Å) of the Optimized Geometry for **3c** Calculated at the PBE0/6-31+G(d) Level of Theory

H	4.04492317	-2.23254457	2.54175508
C	2.97270413	-2.22974783	2.36627930
C	0.17422906	-2.16726182	2.05528080
C	2.35543031	-1.15125028	1.74642408
C	2.18592694	-3.29940858	2.80421378
C	0.80174972	-3.26547216	2.65273647
C	0.95074685	-1.11802140	1.57352098
H	2.65664199	-4.15142091	3.28808992
H	0.19681077	-4.09069041	3.01929345
H	-0.90914668	-2.14168073	2.00932093
C	2.90485766	0.12781239	1.26876093
C	1.84971039	0.91615962	0.87726927
H	1.95770848	1.87569268	0.39210621
C	0.60616711	0.19161823	0.98125942
H	-4.01715125	2.98307630	-1.22439633
C	-3.01463504	3.04604885	-0.81139066
C	-0.39746804	3.22062053	0.21134081
C	-2.36540507	1.91314223	-0.33852036
C	-2.35145325	4.27605315	-0.76484988
C	-1.06278221	4.36347660	-0.24408990
C	-1.03307293	1.98554179	0.13604986
H	-2.84940959	5.17021394	-1.13104609
H	-0.56343968	5.32749354	-0.19309835
H	0.59829644	3.32348159	0.62810967
C	-2.80757054	0.51120438	-0.25000483
C	-1.76787333	-0.22068574	0.26755915
H	-1.79224228	-1.28774030	0.43178230
C	-0.62384246	0.61991394	0.52451123
H	11.01065621	-3.00582628	-1.12349334

C	10.04610172	-2.95204431	-0.62645267
C	7.53340865	-2.83458547	0.64052947
C	9.42840894	-1.72956665	-0.39555858
C	9.40271325	-4.12377115	-0.21477274
C	8.16764816	-4.06327406	0.42541872
C	8.14477908	-1.66523706	0.20179551
H	9.87600558	-5.08681252	-0.38836268
H	7.68580757	-4.97810581	0.76037222
H	6.58126891	-2.81891795	1.15936457
C	9.86065335	-0.35661842	-0.69798662
C	8.87638525	0.49470783	-0.28428240
H	8.90669669	1.56834792	-0.40847611
C	7.75867093	-0.24041282	0.27110638
H	3.24965177	3.07936459	1.89328189
C	4.30285592	3.02079311	1.63290250
C	7.06461994	2.85176240	1.10766204
C	4.87704912	1.81376963	1.25519546
C	5.11196077	4.15752024	1.71716219
C	6.47849555	4.07161056	1.46024628
C	6.26165554	1.72305644	0.97569379
H	4.67500751	5.10916976	2.00887950
H	7.10291147	4.95642319	1.55293149
H	8.13993700	2.80294043	0.97491251
C	4.30281123	0.46153310	1.16523573
C	5.32823894	-0.41139244	0.89599241
H	5.18968828	-1.46099688	0.67924825
C	6.57000559	0.30258869	0.70672653
H	-2.47274829	-2.24623908	-1.49144464
C	-3.55163909	-2.36768455	-1.52732576
C	-6.34429265	-2.64399449	-1.76821652
C	-4.40039989	-1.35182140	-1.10578250
C	-4.10709388	-3.54246733	-2.04275887
C	-5.48828617	-3.67642815	-2.16473736
C	-5.80551147	-1.48892984	-1.21007082
H	-3.45384558	-4.34617021	-2.37278937
H	-5.90834326	-4.58391357	-2.59069331
H	-7.41091612	-2.75098224	-1.93320340
C	-4.10909394	0.00405563	-0.61045466
C	-5.30403575	0.66729362	-0.48026586
H	-5.40954438	1.64782082	-0.03874328
C	-6.41166845	-0.21199463	-0.77863336
H	-11.66869024	2.05639281	0.33026969
C	-10.61723224	2.22910148	0.11825535
C	-7.88917155	2.69459357	-0.40449308
C	-9.76005104	1.17052193	-0.15423838
C	-10.10501444	3.53053872	0.12360369
C	-8.75925071	3.76060715	-0.15053140
C	-8.37781334	1.39299384	-0.37479432
H	-10.76555994	4.36780726	0.33399745
H	-8.37565642	4.77743945	-0.16514757
H	-6.85097733	2.90736149	-0.63457769
C	-10.00940465	-0.27620732	-0.24214048
C	-8.82203801	-0.89145270	-0.51803523
H	-8.69595740	-1.96160191	-0.60762916
C	-7.74620817	0.07564656	-0.59673042
C	11.11522815	0.03596686	-1.34556524
C	13.52008770	0.85059746	-2.55465029
C	12.33388044	-0.57012410	-1.00163068

C	11.12693339	1.05775060	-2.30814506
C	12.31624354	1.46003279	-2.90666291
C	13.52378528	-0.16291996	-1.59766998
H	12.35207413	-1.33865799	-0.23360413
H	10.18733436	1.51937521	-2.60191474
H	12.30243672	2.24699013	-3.65667471
H	14.45917073	-0.63579503	-1.30862852
H	14.44942201	1.16355412	-3.02360715
C	-11.29743615	-0.94834312	-0.04978013
C	-13.74793349	-2.28892255	0.27812775
C	-12.47992923	-0.42926172	-0.60020230
C	-11.36889154	-2.15227646	0.66860212
C	-12.58095827	-2.81485470	0.83137868
C	-13.69113906	-1.09602744	-0.44076643
H	-12.44118187	0.48298548	-1.18941682
H	-10.46514051	-2.55344512	1.12088032
H	-12.61623285	-3.74141603	1.39907268
H	-14.59392619	-0.68449975	-0.88544421
H	-14.69561855	-2.80551313	0.40682342

Supplementary Table 16: Cartesian Coordinates (Å) of the Optimized Geometry for **4a′** Calculated at the PBE0/6-31+G(d) Level of Theory

C	0.86472938	-1.77370554	-0.80053726
C	4.29214959	-0.41509796	-0.10133650
C	2.88038865	-0.66168612	-0.39782558
C	2.81684911	-3.23744493	-0.76410255
H	3.88899496	-3.38613305	-0.66858742
C	0.60644485	-0.32337895	-0.68843080
C	0.61567682	-4.12512920	-1.25352159
H	-0.01997273	-4.96934682	-1.50792820
C	2.25615542	-1.97358951	-0.62587828
C	1.98223663	-4.31841891	-1.06444439
H	2.40772186	-5.31303742	-1.17146772
C	0.05215490	-2.84951609	-1.14156775
H	-1.00404129	-2.71783006	-1.35100035
C	-0.60645521	0.32342829	-0.68839540
C	-2.88039437	0.66171342	-0.39772787
C	-2.25616437	1.97363426	-0.62568687
C	-0.86474153	1.77376360	-0.80038564
C	-1.90424360	-0.29040968	-0.48450835
H	-2.05759001	-1.34018378	-0.27266860
C	-4.29215090	0.41510372	-0.10123643
C	1.90423690	0.29044369	-0.48452143
H	2.05758670	1.34020184	-0.27260511
C	-2.81686034	3.23750058	-0.76380073
H	-3.88900450	3.38618111	-0.66825359
C	7.02196148	0.12110500	0.51010102
C	-0.05217299	2.84960062	-1.14134677
H	1.00401953	2.71793078	-1.35080813
C	-0.61569683	4.12522257	-1.25319088
H	0.01994826	4.96946002	-1.50754287
C	-1.98225314	4.31849786	-1.06407327
H	-2.40774011	5.31312484	-1.17101080
C	-7.02195423	-0.12114106	0.51020119
C	-6.35779482	0.99529207	1.03853453
H	-6.88608046	1.67385358	1.70565188

C	-6.27819407	-0.96458691	-0.33229450
H	-6.74797715	-1.84260474	-0.77438721
C	-4.94668472	-0.70740225	-0.63348551
H	-4.40475962	-1.36580402	-1.30872124
C	-5.02508283	1.26521481	0.73961883
H	-4.53571973	2.12383816	1.19230306
C	5.02509313	-1.26526654	0.73945075
H	4.53573595	-2.12391982	1.19208435
C	6.35780927	-0.99536433	1.03836682
H	6.88610379	-1.67397126	1.70543090
C	6.27818936	0.96460939	-0.33232546
H	6.74796599	1.84265827	-0.77436329
C	4.94667579	0.70744561	-0.63351566
H	4.40474111	1.36589466	-1.30869760
C	-9.51798325	0.80678713	2.07261074
H	-9.47633250	1.81324238	1.63895179
H	-10.56896424	0.59340572	2.30367525
H	-8.97102077	0.82555757	3.02298799
C	9.51800616	-0.80692165	2.07242660
H	10.56899081	-0.59355757	2.30349033
H	8.97105512	-0.82574392	3.02280938
H	9.47634626	-1.81335209	1.63871093
Si	-8.83499327	-0.49647236	0.89367743
Si	8.83500687	0.49640747	0.89357563
C	-8.95733567	-2.20424236	1.68765087
H	-8.55097173	-2.98329056	1.03120069
H	-8.39982008	-2.24407546	2.63090437
H	-10.00131697	-2.46458277	1.90246655
C	-9.82195827	-0.48314530	-0.71504959
H	-10.87790295	-0.71825104	-0.53187423
H	-9.77540676	0.49872724	-1.20076569
H	-9.43731023	-1.22222550	-1.42832572
C	8.95736715	2.20413214	1.68764363
H	8.55099803	2.98321933	1.03124294
H	8.39986469	2.24391462	2.63090700
H	10.00135258	2.46445560	1.90245976
C	9.82195150	0.48316668	-0.71516473
H	10.87789930	0.71825863	-0.53198984
H	9.77539041	-0.49867866	-1.20093493
H	9.43729668	1.22228785	-1.42839475

Supplementary Table 17: Cartesian Coordinates (Å) of the Optimized Geometry for **4b′** Calculated at the PBE0/6-31+G(d) Level of Theory

H	-2.84274888	-3.23927652	-1.68496062
C	-3.80026853	-3.26595252	-1.17566034
C	-6.35301820	-3.40704179	0.00741279
C	-4.46568558	-2.09889953	-0.81712117
C	-4.39392488	-4.50376368	-0.90512983
C	-5.65515284	-4.57454190	-0.31844988
C	-5.75450349	-2.17686131	-0.23428933
H	-3.86935662	-5.41742965	-1.17236166
H	-6.11043700	-5.54352047	-0.13014392
H	-7.35723564	-3.46859943	0.41778364
C	-6.26558050	-0.80722699	-0.07389279
C	-5.33680410	0.05264154	-0.59153212
H	-5.40475808	1.13072845	-0.53110363

C	-4.16217871	-0.66987245	-1.03349416
C	-2.98387737	-0.11100581	-1.47562300
C	-1.69672881	-0.76603074	-1.51882810
H	-1.51427838	-1.77130061	-1.16526963
C	-0.70944959	0.12807196	-1.85280630
C	-2.74232562	1.28108270	-1.90505091
C	-1.69842242	3.67384533	-2.89137763
C	-1.35314296	1.42137776	-2.13656925
C	-3.60086925	2.33422826	-2.20306870
C	-3.06863402	3.53664614	-2.67907768
C	-0.83105859	2.60764916	-2.63610666
H	-4.67634735	2.23395229	-2.10210663
H	-3.73593328	4.36515592	-2.90204498
H	0.22845820	2.70022833	-2.85807890
H	-1.30236133	4.60884055	-3.27949397
H	4.67634985	-2.23405422	-2.10202171
C	3.60087159	-2.33433644	-2.20297583
C	0.83105968	-2.60778121	-2.63599145
C	2.74232734	-1.28117905	-1.90500191
C	3.06863658	-3.53677636	-2.67892947
C	1.69842438	-3.67398734	-2.89121802
C	1.35314406	-1.42148650	-2.13650941
H	3.73593637	-4.36529500	-2.90186228
H	1.30236342	-4.60900065	-3.27929090
H	-0.22845793	-2.70037224	-2.85795497
C	0.70944976	-0.12816893	-1.85280209
C	1.69672866	0.76594951	-1.51886543
H	1.51427771	1.77123449	-1.16535034
C	2.98387833	0.11092850	-1.47563524
C	4.16217885	0.66981654	-1.03353142
C	5.33680717	-0.05267548	-0.59154059
H	5.40476670	-1.13075981	-0.53107131
C	6.26557748	0.80721699	-0.07393012
C	4.46567971	2.09885335	-0.81721377
C	5.65513416	4.57451971	-0.31862910
C	5.75449465	2.17684277	-0.23437943
C	3.80025992	3.26589021	-1.17580055
C	4.39390995	4.50371400	-0.90531380
C	6.35300280	3.40703469	0.00728068
H	2.84274305	3.23919139	-1.68510487
H	3.86933944	5.41736768	-1.17258318
H	7.35721749	3.46861141	0.41765545
H	6.11041292	5.54350729	-0.13035660
C	7.52845571	0.42384397	0.55695554
C	8.00608358	1.08178089	1.69993474
C	8.28581858	-0.64353121	0.04779449
C	9.19101780	0.67965816	2.30993326
H	7.42415820	1.88965592	2.13609067
C	9.46966381	-1.03272552	0.66148473
H	7.94743671	-1.15127339	-0.85263754
C	9.95662113	-0.38281838	1.80823097
H	9.51847459	1.20851932	3.20299853
H	10.02918493	-1.86063140	0.22734686
C	-7.52845835	-0.42382276	0.55697467
C	-8.00610003	-1.08172093	1.69997041
C	-8.28580672	0.64354575	0.04777802
C	-9.19103341	-0.67956735	2.30995060
H	-7.42418673	-1.88959017	2.13615305

C	-9.46965058	1.03277146	0.66145060
H	-7.94741496	1.15125713	-0.85266765
C	-9.95662134	0.38290377	1.80821357
H	-9.51850216	-1.20840113	3.20302781
H	-10.02916064	1.86067016	0.22728475
C	11.91049307	0.11484934	4.14608247
H	11.11397040	0.01568850	4.89335898
H	12.84726587	-0.20040248	4.62203338
H	12.01132288	1.17853200	3.89881812
C	-11.91062092	-0.11479490	4.14595432
H	-11.11414154	-0.01571836	4.89328822
H	-12.84740986	0.20046468	4.62186842
H	-12.01147930	-1.17845658	3.89861131
Si	11.57430994	-0.93540947	2.61687728
Si	-11.57430068	0.93555154	2.61683963
C	11.42849891	-2.75188077	3.10747976
H	10.61928217	-2.90354064	3.83154357
H	11.21760458	-3.38776797	2.23907835
H	12.35935725	-3.11252651	3.56270522
C	12.98441374	-0.73335983	1.37887285
H	13.10078263	0.31431162	1.07725742
H	13.93698529	-1.06753872	1.80870617
H	12.80770908	-1.32051844	0.46949522
C	-12.98436887	0.73369094	1.37876398
H	-13.93693173	1.06791820	1.80857870
H	-12.80758340	1.32089079	0.46942870
H	-13.10080750	-0.31395329	1.07708088
C	-11.42837778	2.75197644	3.10758047
H	-10.61918565	2.90352175	3.83169584
H	-11.21738851	3.38790765	2.23923424
H	-12.35923104	3.11266228	3.56278453

Supplementary Table 18: Cartesian Coordinates (Å) of the Optimized Geometry for **4c′** Calculated at the PBE0/6-31+G(d) Level of Theory

H	-0.80647244	3.36639906	-1.21524540
C	0.15982540	3.36947729	-0.72311797
C	2.69861893	3.46610385	0.48987740
C	0.87032563	2.19391250	-0.50347879
C	0.70998023	4.58951902	-0.31690242
C	1.95893806	4.63690407	0.29742856
C	2.16504728	2.25569192	0.06706802
H	0.15170758	5.50756145	-0.48065365
H	2.36642872	5.59058766	0.62307168
H	3.66957015	3.50889292	0.97458834
C	2.70622195	0.88719237	0.12391166
C	1.76068502	0.04416932	-0.40552233
H	1.86901160	-1.02770131	-0.48065669
C	0.58523422	0.77672196	-0.80851646
C	-0.57538838	0.22636303	-1.31384693
C	-1.87042514	0.86108113	-1.34952489
H	-2.07983038	1.84146534	-0.94602265
C	-2.83701716	-0.03024317	-1.74980943
C	-0.78341643	-1.14422272	-1.82604204
C	-1.76858640	-3.49368332	-2.96897906
C	-2.16587383	-1.29498479	-2.09099269
C	0.09822830	-2.16328593	-2.17261158

C	-0.40506242	-3.34511916	-2.72644141
C	-2.65849263	-2.45806368	-2.66805939
H	1.17080419	-2.05259156	-2.05480133
H	0.28150511	-4.14633648	-2.98717675
H	-3.71200962	-2.55422930	-2.91545804
H	-2.14160518	-4.41079612	-3.41776657
H	-8.25556080	2.25708577	-1.96181637
C	-7.18030904	2.38043388	-2.03595400
C	-4.40822118	2.72356690	-2.39953740
C	-6.30934658	1.32498155	-1.78557043
C	-6.66019543	3.61771386	-2.42845239
C	-5.28893263	3.78922985	-2.60646657
C	-4.91875561	1.50123692	-1.98132080
H	-7.33737007	4.44748712	-2.61372296
H	-4.90199324	4.75198021	-2.93062513
H	-3.34689326	2.84639941	-2.59682126
C	-4.25853795	0.20347660	-1.76342140
C	-5.23727901	-0.72498103	-1.50322612
H	-5.04470288	-1.74664081	-1.20655461
C	-6.53527907	-0.09431466	-1.44632696
C	-7.71276258	-0.69839199	-1.06439207
C	-8.90778600	-0.02366172	-0.60342606
H	-8.99432018	1.04718422	-0.47567645
C	-9.83360329	-0.92889504	-0.16300908
C	-7.99709365	-2.14288701	-0.94664716
C	-9.15635939	-4.66309338	-0.63680521
C	-9.29688267	-2.27767091	-0.39963447
C	-7.30491981	-3.27394887	-1.36465294
C	-7.88390528	-4.53559386	-1.18836300
C	-9.88037457	-3.52971052	-0.25227568
H	-6.33725745	-3.19982454	-1.84951209
H	-7.33871576	-5.42199153	-1.50208866
H	-10.89241913	-3.63310689	0.12935104
H	-9.59990867	-5.64888892	-0.52226485
H	6.52978838	3.59193713	0.75920739
C	7.59282303	3.45007180	0.59571925
C	10.38184759	3.16870622	0.31750191
C	8.16904752	2.18479955	0.61659190
C	8.40649501	4.57053903	0.39484986
C	9.78531005	4.43222924	0.25498235
C	9.57374652	2.05177399	0.48899736
H	7.95602283	5.55925989	0.36505276
H	10.40606999	5.31379933	0.11658210
H	11.46258248	3.07256168	0.25913807
C	9.91517020	0.63269698	0.66853661
C	8.76096773	-0.04682387	0.94409988
H	8.69530773	-1.12275895	1.03624686
C	7.61844076	0.84083881	0.88529122
C	6.29588256	0.46865389	0.97865791
C	5.16180635	1.24012672	0.52422541
H	5.24075602	2.18977675	0.01391095
C	4.00601683	0.50458368	0.61883477
C	5.74354148	-0.81199038	1.46357924
C	4.12760593	-2.91722835	2.32578790
C	4.34554098	-0.78543944	1.24344008
C	6.31408801	-1.88125137	2.14648085
C	5.50105119	-2.94177894	2.55847770
C	3.53655243	-1.82723596	1.68003778

H	7.37053318	-1.89715053	2.39236673
H	5.94592093	-3.78398027	3.08215541
H	2.45819983	-1.78885944	1.55475624
H	3.50581439	-3.74033586	2.66854861
C	-11.11622586	-0.60512023	0.46095228
C	-11.61272223	-1.34488545	1.54435171
C	-11.87550874	0.48526174	0.00605961
C	-12.81801512	-0.99970598	2.14939678
H	-11.03062398	-2.17318706	1.94003570
C	-13.07942202	0.81734543	0.61403408
H	-11.52241491	1.05698637	-0.84923478
C	-13.58569108	0.08495834	1.70122431
H	-13.16025860	-1.59236978	2.99563759
H	-13.63950179	1.66583452	0.22244532
C	11.24728798	0.04051172	0.54781832
C	12.15303596	0.46336709	-0.43615663
C	11.64496606	-0.99849086	1.40491367
C	13.40316324	-0.13704239	-0.55810976
H	11.86035803	1.24256551	-1.13526534
C	12.89684391	-1.58662448	1.27759233
H	10.96852312	-1.32435677	2.19187295
C	13.81166384	-1.17284312	0.29452531
H	14.07050036	0.21226814	-1.34372146
H	13.16650147	-2.38343944	1.96993171
C	16.47152094	-1.21761439	-1.27244452
H	17.45798576	-1.68901006	-1.36269907
H	15.95713663	-1.34869914	-2.23211292
H	16.63488611	-0.14384331	-1.12057168
C	-15.60399644	-0.60998157	3.93185799
H	-14.83113376	-0.56763842	4.70878987
H	-16.55694074	-0.33916932	4.40305008
H	-15.68918011	-1.65062524	3.59636207
Si	15.51026232	-1.99204349	0.15257806
Si	-15.23067623	0.56205594	2.50318689
C	-16.60137115	0.45270931	1.21051690
H	-17.56912388	0.74477166	1.63710227
H	-16.40323840	1.11310431	0.35754031
H	-16.69967360	-0.56765387	0.82150697
C	-15.11333308	2.33387033	3.14213915
H	-14.32704089	2.43120599	3.90002090
H	-14.88171543	3.03844597	2.33412348
H	-16.05996662	2.65185424	3.59664918
C	15.27935684	-3.83884805	-0.15974662
H	14.70960556	-4.31529626	0.64731898
H	14.73782155	-4.01819449	-1.09606453
H	16.24747439	-4.35066313	-0.22754969
C	16.45153048	-1.74404042	1.76943224
H	17.43571306	-2.22746612	1.73048172
H	16.60808249	-0.67914058	1.97826968
H	15.90715568	-2.17038050	2.62081257

Supplementary Table 19: Cartesian Coordinates (Å) of the Optimized Geometry for **4a′** Calculated at the B3LYP/6-31+G(d) Level of Theory

C	0.86181331	-1.78610270	-0.75235516
C	4.31375481	-0.42590274	-0.09225025
C	2.89266961	-0.67482562	-0.36959099
C	2.81469078	-3.26123777	-0.72655669

H	3.88609836	-3.41488764	-0.63805078
C	0.60655872	-0.32799698	-0.64036073
C	0.60065496	-4.14827672	-1.18901784
H	-0.04142471	-4.99144787	-1.43019132
C	2.25970742	-1.99059653	-0.58937568
C	1.97222502	-4.34514181	-1.01415736
H	2.39446613	-5.34126152	-1.11947586
C	0.04116230	-2.86617929	-1.07594322
H	-1.01742708	-2.73515241	-1.26922372
C	-0.60656510	0.32808185	-0.64032942
C	-2.89267418	0.67487253	-0.36950057
C	-2.25972642	1.99066446	-0.58920045
C	-0.86183355	1.78619406	-0.75222306
C	-1.91363135	-0.28059029	-0.44704064
H	-2.07289048	-1.33028715	-0.24491161
C	-4.31375288	0.42591734	-0.09215584
C	1.91363237	0.28065052	-0.44704102
H	2.07290343	1.33032981	-0.24483291
C	-2.81472426	3.26131037	-0.72627979
H	-3.88613152	3.41494399	-0.63774118
C	7.06477912	0.11797963	0.48163381
C	-0.04120066	2.86630124	-1.07575668
H	1.01738497	2.73529911	-1.26907165
C	-0.60070743	4.14840157	-1.18872865
H	0.04135903	4.99159536	-1.42985812
C	-1.97227499	4.34524236	-1.01382245
H	-2.39452760	5.34136555	-1.11906221
C	-7.06476308	-0.11803139	0.48173262
C	-6.40511470	1.00127527	1.02168807
H	-6.94095487	1.67979513	1.68192309
C	-6.30274874	-0.95956972	-0.35411930
H	-6.76218022	-1.83769689	-0.80574646
C	-4.96375269	-0.69932345	-0.63643331
H	-4.41428303	-1.35752323	-1.30493433
C	-5.06488384	1.27435997	0.74178659
H	-4.58704078	2.13399206	1.20310443
C	5.06489048	-1.27441063	0.74162156
H	4.58704627	-2.13407020	1.20288695
C	6.40512824	-1.00135823	1.02152150
H	6.94097207	-1.67992925	1.68170094
C	6.30275975	0.95958394	-0.35414707
H	6.76219255	1.83773842	-0.80571973
C	4.96375673	0.69937034	-0.63645839
H	4.41428295	1.35762229	-1.30490467
C	-9.60097983	0.80777014	2.01178106
H	-9.55670251	1.81538489	1.57977021
H	-10.65550674	0.58938683	2.22417342
H	-9.07179142	0.83051355	2.97267434
C	9.60100766	-0.80795017	2.01158435
H	10.65554140	-0.58959525	2.22397213
H	9.07183550	-0.83074620	2.97248531
H	9.55670860	-1.81553720	1.57951111
Si	-8.88859025	-0.49837217	0.84054159
Si	8.88861639	0.49827557	0.84043896
C	-9.02326285	-2.21148849	1.64079181
H	-8.60846557	-2.99433901	0.99332612
H	-8.47976542	-2.24985765	2.59301155
H	-10.07095908	-2.47055134	1.84207931

C	-9.86019831	-0.49016180	-0.78722525
H	-10.91885338	-0.72489209	-0.61555376
H	-9.80958050	0.49076270	-1.27593439
H	-9.46830536	-1.23106398	-1.49545924
C	9.02332333	2.21133935	1.64079566
H	8.60852600	2.99423636	0.99338623
H	8.47984157	2.24965514	2.59302650
H	10.07102612	2.47037578	1.84208290
C	9.86019921	0.49015619	-0.78734356
H	10.91885931	0.72486495	-0.61567404
H	9.80956367	-0.49073749	-1.27611274
H	9.46830271	1.23110626	-1.49552552

Supplementary Table 20: Cartesian Coordinates (Å) of the Optimized Geometry for **4b′** Calculated at the B3LYP/6-31+G(d) Level of Theory

H	-2.83077218	-3.26167025	-1.52349720
C	-3.80507506	-3.28713470	-1.04897129
C	-6.39758450	-3.43283546	0.05881228
C	-4.49024435	-2.11614415	-0.72732846
C	-4.40031531	-4.53028585	-0.78358986
C	-5.68196562	-4.60343327	-0.23423833
C	-5.80025732	-2.19708440	-0.17923257
H	-3.86029618	-5.44254361	-1.02321049
H	-6.13721654	-5.57275100	-0.04793192
H	-7.41127337	-3.49829809	0.44273177
C	-6.32539557	-0.82382572	-0.04417074
C	-5.38034348	0.03781022	-0.53922456
H	-5.45827499	1.11483632	-0.49425067
C	-4.18531277	-0.68047964	-0.94689780
C	-2.99905433	-0.11393448	-1.37292363
C	-1.70475150	-0.76508515	-1.40588147
H	-1.52414348	-1.77134724	-1.05828813
C	-0.71149008	0.12994535	-1.73735193
C	-2.75438336	1.28458147	-1.80472668
C	-1.70245566	3.68915090	-2.78265128
C	-1.35835683	1.42754725	-2.02698298
C	-3.61211431	2.34307971	-2.10460364
C	-3.07723662	3.55050498	-2.57778972
C	-0.83426129	2.61942142	-2.52163130
H	-4.68729618	2.24562254	-2.00643083
H	-3.74450871	4.37876923	-2.80151600
H	0.22615022	2.71592197	-2.73384846
H	-1.30337485	4.62481894	-3.16584615
H	4.68731106	-2.24592361	-2.00625726
C	3.61212822	-2.34339353	-2.10439802
C	0.83427020	-2.61979047	-2.52135012
C	2.75439879	-1.28486680	-1.80461430
C	3.07724731	-3.55087106	-2.57744710
C	1.70246368	-3.68954477	-2.78226989
C	1.35836909	-1.42786180	-2.02683626
H	3.74451898	-4.37915596	-2.80109824
H	1.30338031	-4.62525441	-3.16536052
H	-0.22614370	-2.71631630	-2.73354376
C	0.71150169	-0.13022944	-1.73734267
C	1.70476450	0.76484272	-1.40599069
H	1.52415892	1.77114762	-1.05851769
C	2.99906655	0.11369609	-1.37295496

C	4.18531690	0.68030477	-0.94699079
C	5.38036179	-0.03790952	-0.53922327
H	5.45831923	-1.11492744	-0.49411697
C	6.32539457	0.82380811	-0.04427432
C	4.49020939	2.11599966	-0.72757624
C	5.68185725	4.60337646	-0.23475982
C	5.80021975	2.19703743	-0.17949011
C	3.80500274	3.28693359	-1.04934323
C	4.40020767	4.53013133	-0.78410062
C	6.39751089	3.43283182	0.05841964
H	2.83069736	3.26138607	-1.52386114
H	3.86016079	5.44234686	-1.02381941
H	7.41119733	3.49836579	0.44233376
H	6.13707959	5.57272804	-0.04855974
C	7.61676766	0.43671356	0.53779813
C	8.14243207	1.08515524	1.67060649
C	8.35979740	-0.62758099	-0.01052646
C	9.35475032	0.67861145	2.23082721
H	7.58070365	1.88882320	2.13814129
C	9.57093902	-1.02111387	0.55357993
H	7.98991689	-1.12938742	-0.90123326
C	10.10621101	-0.38074531	1.68984968
H	9.71410078	1.20215402	3.11407093
H	10.11194523	-1.84357575	0.08779383
C	-7.61675941	-0.43663081	0.53785557
C	-8.14244387	-1.08493146	1.67073542
C	-8.35975967	0.62762227	-0.01058993
C	-9.35475344	-0.67829393	2.23090657
H	-7.58073794	-1.88856139	2.13836258
C	-9.57089282	1.02124984	0.55346901
H	-7.98986339	1.12931957	-0.90135164
C	-10.10618551	0.38102110	1.68980773
H	-9.71412020	-1.20172878	3.11420755
H	-10.11187660	1.84367377	0.08759007
C	12.17729673	0.11567094	3.94684610
H	11.41712377	0.02392640	4.73270467
H	13.13419588	-0.20485810	4.37862340
H	12.27293282	1.17908252	3.69410015
C	-12.17727641	-0.11507546	3.94686884
H	-11.41710017	-0.02324229	4.73271386
H	-13.13416958	0.20552399	4.37860714
H	-12.27292985	-1.17851793	3.69425927
Si	11.76140072	-0.93960289	2.43075470
Si	-11.76136664	0.93999820	2.43064171
C	11.63365887	-2.76131413	2.93875984
H	10.85696118	-2.90903101	3.69933945
H	11.38398923	-3.40307243	2.08440005
H	12.58354018	-3.12115228	3.35533657
C	13.12505361	-0.74720041	1.12815249
H	13.23297618	0.29904141	0.81618050
H	14.09431492	-1.08156870	1.52063846
H	12.91148232	-1.33837360	0.22870592
C	-13.12502254	0.74744591	1.12806480
H	-14.09428307	1.08185962	1.52051403
H	-12.91145274	1.33851489	0.22854939
H	-13.23294570	-0.29883205	0.81621426
C	-11.63359923	2.76177291	2.93841256
H	-10.85690567	2.90957491	3.69897988

H	-11.38391197	3.40341749	2.08397241
H	-12.58347805	3.12168036	3.35493493

Supplementary Table 21: Cartesian Coordinates (Å) of the Optimized Geometry for **4c′** Calculated at the B3LYP/6-31+G(d) Level of Theory

H	-0.82041562	3.34471078	-1.09931908
C	0.15864938	3.34968442	-0.63508745
C	2.73124946	3.45408060	0.51829747
C	0.87975413	2.17225237	-0.43654920
C	0.71533182	4.57559949	-0.24065145
C	1.98246073	4.62696692	0.34341864
C	2.19138160	2.23767902	0.10831187
H	0.14880173	5.49110599	-0.38916746
H	2.39603474	5.58128907	0.65915997
H	3.71276067	3.50179136	0.97896916
C	2.73831233	0.86436944	0.15739373
C	1.77698427	0.01845243	-0.35064392
H	1.88550925	-1.05190857	-0.42753785
C	0.58899787	0.74774279	-0.73606015
C	-0.58006056	0.19158837	-1.22981027
C	-1.87854759	0.82816720	-1.26664620
H	-2.08280767	1.81346317	-0.87619445
C	-2.85387461	-0.06237199	-1.66331819
C	-0.79490481	-1.18715065	-1.73750379
C	-1.79263280	-3.55081350	-2.86422206
C	-2.18376137	-1.33630468	-1.99945073
C	0.08335536	-2.21727969	-2.07560951
C	-0.42495385	-3.40512603	-2.62287456
C	-2.68058176	-2.50654353	-2.56759073
H	1.15550572	-2.11266829	-1.95547179
H	0.25918403	-4.21061344	-2.87649766
H	-3.73433530	-2.60417283	-2.81009206
H	-2.17054377	-4.46976848	-3.30491597
H	-8.27465606	2.26269762	-1.90609198
C	-7.19918754	2.37852750	-1.97797992
C	-4.41778876	2.71040001	-2.32604623
C	-6.33352395	1.31474218	-1.72277438
C	-6.67112395	3.61707728	-2.37131201
C	-5.29466100	3.78300233	-2.54210830
C	-4.93551952	1.48624089	-1.90938175
H	-7.34452115	4.44902924	-2.56022288
H	-4.90058776	4.74343428	-2.86432040
H	-3.35522372	2.83145349	-2.51339873
C	-4.27819045	0.18144018	-1.68168389
C	-5.26749100	-0.74277949	-1.42261772
H	-5.08168569	-1.76508897	-1.12866956
C	-6.56902486	-0.10887825	-1.37740046
C	-7.75619296	-0.71239527	-1.00692109
C	-8.96496804	-0.03251058	-0.57619160
H	-9.05491431	1.03894161	-0.46646383
C	-9.90808753	-0.93255083	-0.14970527
C	-8.04834028	-2.16177815	-0.88098857
C	-9.21997507	-4.68658169	-0.56372268
C	-9.36587416	-2.28966741	-0.36072417
C	-7.34506515	-3.30323002	-1.26358183
C	-7.93061025	-4.56653950	-1.08593863
C	-9.95322180	-3.54375784	-0.21029086

H	-6.36388840	-3.23802052	-1.71988376
H	-7.37689934	-5.45661501	-1.37298484
H	-10.97223292	-3.64344262	0.15145305
H	-9.66752424	-5.67010787	-0.44589208
H	6.57197603	3.59367121	0.69350753
C	7.63590626	3.45929246	0.53496663
C	10.43296246	3.20059646	0.25378759
C	8.22365798	2.19532258	0.56700357
C	8.44104785	4.58996185	0.32639310
C	9.82443635	4.46287013	0.18540056
C	9.63468678	2.07350963	0.43536999
H	7.98052950	5.57374252	0.28964832
H	10.43790191	5.34826719	0.03960223
H	11.51361231	3.11492855	0.18959152
C	9.99028585	0.65244030	0.61916207
C	8.83750306	-0.03635923	0.89758031
H	8.78208978	-1.11099294	0.99846536
C	7.68164934	0.84152637	0.84190365
C	6.35888461	0.45566157	0.94920577
C	5.20807467	1.22176277	0.51529759
H	5.27257193	2.17677366	0.01585817
C	4.05212271	0.48017371	0.62588315
C	5.81803556	-0.83590349	1.43952091
C	4.22396868	-2.96491621	2.31580993
C	4.41110503	-0.81638745	1.24130304
C	6.40653390	-1.91233917	2.10364597
C	5.60493541	-2.98371231	2.52456529
C	3.61530794	-1.87039728	1.68500645
H	7.46714825	-1.92774792	2.32727980
H	6.06467751	-3.82731735	3.03280080
H	2.53565491	-1.84061244	1.57593933
H	3.61192713	-3.79376252	2.66206466
C	-11.21162715	-0.59510925	0.43556916
C	-11.74812133	-1.32056158	1.51541656
C	-11.95634575	0.49651044	-0.05374243
C	-12.97260050	-0.96190303	2.08174245
H	-11.18582415	-2.14798096	1.93867674
C	-13.17962136	0.84185562	0.51565051
H	-11.57790216	1.05869201	-0.90386052
C	-13.72593453	0.12399021	1.59916960
H	-13.34012071	-1.54519138	2.92318965
H	-13.72141446	1.68828949	0.09604619
C	11.33147881	0.06613872	0.50278812
C	12.24220349	0.49012756	-0.48255317
C	11.73723139	-0.97102422	1.36602031
C	13.49947270	-0.10521344	-0.59985701
H	11.95057084	1.26560298	-1.18513331
C	12.99624689	-1.55384752	1.24332088
H	11.06338428	-1.30042237	2.15303918
C	13.91599336	-1.13932003	0.25847369
H	14.16406033	0.24755922	-1.38550991
H	13.26619756	-2.34604708	1.94017220
C	16.59430525	-1.17832092	-1.30854613
H	17.58321979	-1.64728801	-1.39185682
H	16.08560997	-1.31506613	-2.27104536
H	16.75421235	-0.10266733	-1.16267720
C	-15.82458043	-0.53601423	3.78748516
H	-15.07654595	-0.48819408	4.58873793

H	-16.79064049	-0.25271646	4.22484516
H	-15.90598578	-1.58174215	3.46544205
Si	15.62527222	-1.95235095	0.12214428
Si	-15.39779348	0.61850425	2.34880385
C	-16.73952551	0.49703259	1.01531315
H	-17.71804648	0.79620779	1.41316664
H	-16.51865910	1.14679491	0.15908531
H	-16.83172544	-0.52798301	0.63526940
C	-15.29637687	2.40501625	2.97410028
H	-14.53302302	2.51098299	3.75488179
H	-15.04033895	3.10250404	2.16658223
H	-16.25617253	2.72859700	3.39762573
C	15.40874782	-3.80994756	-0.18695954
H	14.84208270	-4.29120036	0.62014480
H	14.87030822	-3.99745626	-1.12423671
H	16.38189054	-4.31387226	-0.25280142
C	16.57027685	-1.69370633	1.74489195
H	17.55799222	-2.17141078	1.70582140
H	16.72213262	-0.62704856	1.95170203
H	16.03054056	-2.12091081	2.59946406

Supplementary Table 22: Cartesian Coordinates (Å) of the Optimized Geometry for **4a′** Calculated at the CAM-B3LYP/6-31+G(d) Level of Theory

C	0.85905413	-1.78349459	-0.69281051
C	4.30836345	-0.41354753	-0.09181260
C	2.88116261	-0.65832340	-0.34723413
C	2.81051753	-3.24213894	-0.64282583
H	3.88247103	-3.38482507	-0.55143390
C	0.59919914	-0.32422244	-0.60404412
C	0.60803892	-4.14551737	-1.07043504
H	-0.03028355	-4.99649460	-1.28856256
C	2.24937584	-1.97816905	-0.53667417
C	1.97594683	-4.33177693	-0.89892614
H	2.40104308	-5.32762245	-0.98255838
C	0.04421160	-2.87000142	-0.98381155
H	-1.01545474	-2.75126263	-1.17173516
C	-0.59920805	0.32426066	-0.60401777
C	-2.88116770	0.65834610	-0.34715595
C	-2.24938569	1.97820225	-0.53654014
C	-0.85906596	1.78353734	-0.69270792
C	-1.91430631	-0.28818964	-0.42968343
H	-2.07889021	-1.34063234	-0.25311671
C	-4.30836421	0.41355432	-0.09172498
C	1.91430061	0.28821763	-0.42969474
H	2.07888892	1.34065052	-0.25307445
C	-2.81053085	3.24217664	-0.64262115
H	-3.88248310	3.38485689	-0.55120633
C	7.05222915	0.11797952	0.43736621
C	-0.04423003	2.87006000	-0.98366923
H	1.01543290	2.75133236	-1.17161824
C	-0.60806053	4.14557938	-1.07022045
H	0.03025715	4.99656824	-1.28831659
C	-1.97596587	4.33182856	-0.89868040
H	-2.40106491	5.32767764	-0.98225635
C	-7.05222031	-0.11800803	0.43746940
C	-6.38037222	0.94676329	1.05040928
H	-6.90779498	1.58744474	1.75253452

C	-6.30830505	-0.91031463	-0.44936077
H	-6.78226313	-1.74739036	-0.95833586
C	-4.96898652	-0.65459773	-0.71146023
H	-4.42659217	-1.27432865	-1.41991404
C	-5.03965233	1.21392663	0.79220214
H	-4.54768455	2.03451123	1.30562415
C	5.03966394	-1.21396992	0.79205884
H	4.54770284	-2.03458272	1.30544237
C	6.38038868	-0.94682392	1.05025866
H	6.90782192	-1.58754628	1.75233869
C	6.30830177	0.91033547	-0.44940970
H	6.78225413	1.74743670	-0.95834819
C	4.96897833	0.65463608	-0.71150126
H	4.42657417	1.27440621	-1.41991329
C	-9.56681059	0.75861221	1.99410521
H	-9.50489188	1.78350390	1.61151038
H	-10.62391078	0.54727644	2.19236439
H	-9.04171922	0.72683943	2.95528929
C	9.56688081	-0.75881853	1.99380329
H	10.62398783	-0.54750359	2.19204853
H	9.04182861	-0.72717581	2.95501305
H	9.50495093	-1.78365957	1.61107472
Si	-8.87293539	-0.49325868	0.77048165
Si	8.87294957	0.49321207	0.77037498
C	-9.02590873	-2.23132233	1.48325832
H	-8.61908398	-2.98435684	0.79889635
H	-8.48339537	-2.32163300	2.43077052
H	-10.07451996	-2.48952842	1.67181807
C	-9.83161295	-0.39824481	-0.84940126
H	-10.89155397	-0.63137063	-0.69455906
H	-9.77010250	0.60297662	-1.28989695
H	-9.44316790	-1.10690039	-1.58952748
C	9.02591894	2.23117800	1.48339098
H	8.61905403	2.98430040	0.79914959
H	8.48343839	2.32134519	2.43093567
H	10.07453224	2.48937734	1.67194874
C	9.83157702	0.39844106	-0.84955160
H	10.89152037	0.63155498	-0.69470748
H	9.77006334	-0.60271704	-1.29019058
H	9.44310382	1.10720051	-1.58956355

Supplementary Table 23: Cartesian Coordinates (Å) of the Optimized Geometry for **4b′** Calculated at the CAM-B3LYP/6-31+G(d) Level of Theory

H	-2.73981750	-3.27358257	-1.19364496
C	-3.72524078	-3.28167979	-0.74516241
C	-6.33079678	-3.40048978	0.30509333
C	-4.44169073	-2.11062173	-0.53509991
C	-4.29906170	-4.50784792	-0.39870942
C	-5.58688459	-4.56820550	0.12321507
C	-5.75466949	-2.18143732	-0.01879331
H	-3.73462653	-5.42253520	-0.55317274
H	-6.02412380	-5.52983812	0.37505538
H	-7.35011636	-3.45415252	0.67365775
C	-6.31622525	-0.81700350	0.00137327
C	-5.39276549	0.02727286	-0.52074908
H	-5.50512363	1.10041595	-0.55976370
C	-4.16213665	-0.68655205	-0.85021384

C	-2.99589776	-0.12616208	-1.28105640
C	-1.68439894	-0.76398469	-1.28363187
H	-1.49197956	-1.75801627	-0.91070272
C	-0.71665437	0.12100279	-1.64151339
C	-2.75822505	1.25862833	-1.75992247
C	-1.71032503	3.62894506	-2.79258947
C	-1.37066761	1.40116371	-1.97751536
C	-3.61382244	2.30108425	-2.09356269
C	-3.08067840	3.49059329	-2.59573557
C	-0.84465680	2.57500208	-2.49522047
H	-4.68825044	2.20668427	-1.99904646
H	-3.74834629	4.30884945	-2.84835890
H	0.21812859	2.66713261	-2.69402875
H	-1.31271538	4.55463842	-3.19802954
H	4.68835247	-2.20619389	-1.99936454
C	3.61392937	-2.30056887	-2.09395987
C	0.84478449	-2.57438494	-2.49582905
C	2.75831503	-1.25819763	-1.76009809
C	3.08081112	-3.48995026	-2.59646235
C	1.71046788	-3.62825228	-2.79342150
C	1.37076873	-1.40067809	-1.97779877
H	3.74849208	-4.30814217	-2.84925920
H	1.31287910	-4.55384262	-3.19911717
H	-0.21799072	-2.66646491	-2.69471527
C	0.71673766	-0.12060266	-1.64150610
C	1.68446318	0.76429380	-1.28334858
H	1.49202418	1.75823085	-0.91017761
C	2.99596198	0.12647085	-1.28086552
C	4.16217656	0.68675332	-0.84981751
C	5.39279073	-0.02715164	-0.52046960
H	5.50515671	-1.10028387	-0.55975549
C	6.31621895	0.81699430	0.00191889
C	4.44170735	2.11074256	-0.53431950
C	5.58685400	4.56815988	0.12469991
C	5.75465748	2.18143017	-0.01792308
C	3.72526340	3.28185206	-0.74411533
C	4.29906015	4.50793242	-0.39731142
C	6.33076159	3.40040014	0.30631432
H	2.73986435	3.27386732	-1.19265323
H	3.73462930	5.42265734	-0.55156729
H	7.35006021	3.45396990	0.67495059
H	6.02407448	5.52972884	0.37681551
C	7.63911222	0.43574456	0.51755613
C	8.14119893	0.97205942	1.70794330
C	8.42346880	-0.50425711	-0.16267938
C	9.37951867	0.57312471	2.20084543
H	7.54462599	1.68461057	2.26949997
C	9.65978402	-0.89226223	0.33598461
H	8.06352815	-0.91549972	-1.10154760
C	10.17338255	-0.36544650	1.53029052
H	9.72645379	1.00674623	3.13528445
H	10.23776368	-1.62075307	-0.22932479
C	-7.63914223	-0.43587771	0.51704084
C	-8.14130434	-0.97251226	1.70725201
C	-8.42345303	0.50431341	-0.16298621
C	-9.37965055	-0.57370243	2.20018864
H	-7.54477654	-1.68523000	2.26864489
C	-9.65979566	0.89219080	0.33570861

H	-8.06346068	0.91579785	-1.10172863
C	-10.17346124	0.36506939	1.52985144
H	-9.72665429	-1.00759367	3.13447696
H	-10.23774474	1.62082365	-0.22944883
C	12.24654730	-0.01356282	3.78705638
H	11.50661775	-0.22832967	4.56614412
H	13.22441235	-0.32728925	4.17018799
H	12.28322531	1.07282894	3.64915592
C	-12.24760078	0.01115583	3.78539605
H	-11.50802361	0.22484087	4.56511611
H	-13.22559427	0.32460294	4.16842842
H	-12.28440342	-1.07506016	3.64615370
Si	11.85963814	-0.91516304	2.17987395
Si	-11.85972900	0.91464034	2.17950375
C	11.82817077	-2.77469244	2.48499604
H	11.07462718	-3.04212162	3.23392293
H	11.59248787	-3.32901897	1.56952708
H	12.79997748	-3.13168313	2.84546606
C	13.17827651	-0.51736960	0.89345003
H	13.22904964	0.55892129	0.69529176
H	14.16919800	-0.84267767	1.23113467
H	12.97470932	-1.01750475	-0.06011689
C	-13.17803107	0.51903476	0.89206237
H	-14.16893505	0.84436781	1.22977386
H	-12.97388592	1.02027461	-0.06080007
H	-13.22921537	-0.55698379	0.69253794
C	-11.82764789	2.77376432	2.48703042
H	-11.07424480	3.03993514	3.23654714
H	-11.59143322	3.32916070	1.57234666
H	-12.79943253	3.13067850	2.84763629

Supplementary Table 24: Cartesian Coordinates (Å) of the Optimized Geometry for **4c′** Calculated at the CAM-B3LYP/6-31+G(d) Level of Theory

H	-0.88920892	3.29730678	-0.67553686
C	0.10094312	3.27485858	-0.23918814
C	2.69212466	3.32375817	0.84792483
C	0.84643528	2.10433021	-0.17377694
C	0.64406058	4.46322043	0.25531625
C	1.92098176	4.48670301	0.80597327
C	2.16079567	2.14785406	0.34128757
H	0.05723959	5.37569164	0.21010024
H	2.32420153	5.41514101	1.19930703
H	3.68693869	3.34295373	1.28021769
C	2.74169684	0.79375430	0.24305930
C	1.80938052	-0.02196685	-0.31445038
H	1.94839029	-1.07617828	-0.49145606
C	0.57998120	0.70667141	-0.60082544
C	-0.57045999	0.17105438	-1.10436161
C	-1.88507322	0.80077885	-1.10202186
H	-2.09576496	1.76791112	-0.67384266
C	-2.83783788	-0.06439660	-1.53994527
C	-0.78600145	-1.18145666	-1.68053160
C	-1.79515852	-3.48355039	-2.89427155
C	-2.16718115	-1.31686439	-1.94072374
C	0.08322537	-2.19510507	-2.06487574
C	-0.43095974	-3.35116088	-2.65760744
C	-2.67383926	-2.45552619	-2.54768001

H	1.15522605	-2.10399508	-1.94520261
H	0.24849662	-4.14600022	-2.95015874
H	-3.73099605	-2.53831491	-2.77789316
H	-2.17742136	-4.38180898	-3.37000598
H	-8.24629168	2.25763901	-1.87256664
C	-7.17054662	2.36300453	-1.93519117
C	-4.39437263	2.67547132	-2.25222843
C	-6.31666224	1.30428985	-1.65201598
C	-6.63298191	3.58583097	-2.34370013
C	-5.25923814	3.74310150	-2.49852029
C	-4.92525196	1.46725510	-1.82653618
H	-7.29941033	4.41630305	-2.55655663
H	-4.85787875	4.69559794	-2.83171087
H	-3.32790130	2.78558519	-2.41975788
C	-4.27183542	0.17037196	-1.56098902
C	-5.24260329	-0.74094982	-1.28646462
H	-5.05340364	-1.75669003	-0.97548809
C	-6.55739143	-0.11043203	-1.27312012
C	-7.73012494	-0.70416193	-0.90964206
C	-8.97246516	-0.02015519	-0.56174098
H	-9.09023033	1.05264470	-0.52960902
C	-9.90275093	-0.90249318	-0.12089168
C	-8.00827072	-2.14784642	-0.70000233
C	-9.15391904	-4.65003870	-0.24141414
C	-9.33261172	-2.25993108	-0.22154179
C	-7.28007149	-3.29828009	-0.97361590
C	-7.85454288	-4.54807041	-0.72681272
C	-9.90896072	-3.50095559	0.00299431
H	-6.28458278	-3.25477145	-1.39717686
H	-7.28123907	-5.44738336	-0.93088486
H	-10.93644958	-3.58457300	0.34204267
H	-9.59131621	-5.62869456	-0.06753383
H	6.54644524	3.53649959	0.56475776
C	7.60855838	3.40628258	0.39968346
C	10.39313706	3.17059861	0.08934355
C	8.20932172	2.15572575	0.46266421
C	8.39578161	4.53316442	0.14834695
C	9.77304954	4.41800023	-0.00798695
C	9.61044755	2.04898551	0.31807652
H	7.92449471	5.50961862	0.08893095
H	10.37301827	5.30482539	-0.18939399
H	11.47228171	3.08932894	0.00829587
C	9.99003174	0.63942099	0.53375829
C	8.86776924	-0.05831520	0.83681277
H	8.83652307	-1.12931699	0.97014544
C	7.68840905	0.80086015	0.77644259
C	6.39164645	0.40029918	0.90874665
C	5.21165597	1.16246388	0.51592252
H	5.24639404	2.13090455	0.04162365
C	4.08514279	0.41420378	0.65152305
C	5.87230253	-0.90436037	1.38904929
C	4.31526254	-3.05646019	2.24063978
C	4.46950616	-0.88901977	1.23009570
C	6.47950633	-1.99088268	2.00618483
C	5.69533486	-3.07171627	2.41619235
C	3.68977487	-1.95388395	1.65596771
H	7.54419068	-2.01102741	2.20188766
H	6.17051529	-3.92519810	2.89045592

H	2.60909876	-1.92537021	1.56336919
H	3.71737898	-3.89809459	2.57749641
C	-11.23964338	-0.56397807	0.38824496
C	-11.76653850	-1.18496335	1.52558240
C	-12.01321580	0.41825593	-0.24290552
C	-13.01873835	-0.82737096	2.01507988
H	-11.17908931	-1.93241154	2.05016087
C	-13.26347293	0.76456338	0.25169982
H	-11.63350604	0.89633493	-1.14146671
C	-13.80225967	0.15236126	1.39301119
H	-13.38508570	-1.32707268	2.90817872
H	-13.83218470	1.52868607	-0.27460322
C	11.34641870	0.08406436	0.41945168
C	12.20334611	0.45851318	-0.62084911
C	11.80681868	-0.86378564	1.34202417
C	13.47079230	-0.10365417	-0.73511572
H	11.86436395	1.17289729	-1.36497571
C	13.07517426	-1.41547965	1.22098201
H	11.16676102	-1.15118240	2.17141239
C	13.94314248	-1.05164506	0.18083574
H	14.09977709	0.20726762	-1.56533962
H	13.39338992	-2.14376816	1.96440232
C	16.55234928	-1.12943519	-1.46228519
H	17.55192202	-1.57114445	-1.54699595
H	16.01635818	-1.35385322	-2.39123275
H	16.67989242	-0.04281212	-1.40237192
C	-15.91716227	-0.35337691	3.58048301
H	-15.19552659	-0.17710824	4.38594086
H	-16.90611496	-0.07274704	3.96080593
H	-15.93875577	-1.43014076	3.37868996
Si	15.66391333	-1.81923660	0.04784586
Si	-15.50823319	0.64501432	2.03726328
C	-16.79497488	0.30884186	0.70190576
H	-17.79660750	0.60291613	1.03662291
H	-16.57754669	0.86619447	-0.21622154
H	-16.82849328	-0.75447325	0.44019607
C	-15.50524186	2.48374973	2.45048906
H	-14.77039125	2.71533090	3.22932975
H	-15.25805295	3.09326587	1.57397666
H	-16.48843472	2.80835700	2.81068448
C	15.49555395	-3.68956575	-0.11135881
H	14.97045448	-4.12142906	0.74809299
H	14.93354231	-3.96338863	-1.01102145
H	16.47905886	-4.17003459	-0.17178035
C	16.64522729	-1.41284669	1.60468877
H	17.64453191	-1.86222070	1.56943981
H	16.76894660	-0.33109375	1.72616687
H	16.14617261	-1.78928408	2.50475083

Supplementary Table 25: Cartesian Coordinates (Å) of the Optimized Geometry for **4a′** Calculated at the M06-2X/6-31+G(d) Level of Theory

C	0.87164519	-1.77232742	-0.78588179
C	4.30052178	-0.39519484	-0.10414834
C	2.88105599	-0.63717532	-0.39598071
C	2.83521416	-3.21992234	-0.73156337
H	3.90829137	-3.35669408	-0.63245844
C	0.60406957	-0.31682941	-0.68648564

C	0.63942191	-4.12797713	-1.21287488
H	0.01151477	-4.97902997	-1.45911547
C	2.26210116	-1.96064876	-0.61030632
C	2.00743760	-4.30999931	-1.01951689
H	2.43847147	-5.30225412	-1.11480784
C	0.06495978	-2.85549741	-1.11649409
H	-0.99062691	-2.73333471	-1.33098617
C	-0.60410747	0.31665302	-0.68643428
C	-2.88107588	0.63709261	-0.39585663
C	-2.26205224	1.96055177	-0.61007091
C	-0.87160658	1.77217003	-0.78564240
C	-1.90999050	-0.30911127	-0.48817785
H	-2.06131280	-1.36100270	-0.28659332
C	-4.30055120	0.39516457	-0.10402751
C	1.90992812	0.30898741	-0.48825557
H	2.06119737	1.36088183	-0.28662780
C	-2.83508697	3.21987187	-0.73121048
H	-3.90815702	3.35669979	-0.63210491
C	7.02740994	0.12318690	0.49810423
C	-0.06483894	2.85532310	-1.11609667
H	0.99075219	2.73310854	-1.33055470
C	-0.63922210	4.12784726	-1.21236180
H	-0.01125532	4.97888927	-1.45848757
C	-2.00723516	4.30992845	-1.01903161
H	-2.43820589	5.30222004	-1.11422490
C	-7.02745979	-0.12311845	0.49821649
C	-6.35018170	0.96709508	1.06202358
H	-6.86929850	1.62922076	1.75215677
C	-6.30106570	-0.94960172	-0.37475099
H	-6.78685094	-1.80593987	-0.84124678
C	-4.96643923	-0.70016832	-0.67340233
H	-4.43164381	-1.34122794	-1.36988401
C	-5.01380073	1.22865448	0.76726521
H	-4.50784426	2.06562460	1.24151036
C	5.01382311	-1.22870820	0.76707976
H	4.50791444	-2.06573647	1.24127312
C	6.35019356	-0.96710047	1.06184264
H	6.86935131	-1.62924699	1.75192504
C	6.30096430	0.94969365	-0.37479809
H	6.78670055	1.80608949	-0.84123899
C	4.96634767	0.70021206	-0.67345349
H	4.43151191	1.34129291	-1.36988464
C	-9.50018781	0.78542188	2.08874334
H	-9.44465680	1.80017872	1.67908460
H	-10.55169227	0.57859610	2.31788369
H	-8.94637032	0.76971390	3.03409209
C	9.50019940	-0.78533550	2.08854562
H	10.55169687	-0.57847435	2.31768605
H	8.94639318	-0.76971585	3.03390239
H	9.44471188	-1.80006821	1.67882122
Si	-8.83876430	-0.49214207	0.87502773
Si	8.83869979	0.49227617	0.87492179
C	-8.96978753	-2.21806693	1.61843344
H	-8.57654030	-2.97643427	0.93197668
H	-8.40252957	-2.28916456	2.55263911
H	-10.01348958	-2.47367309	1.83436770
C	-9.82372768	-0.43041166	-0.72951714
H	-10.88048120	-0.65947751	-0.55047945

H	-9.76464573	0.56120384	-1.19079529
H	-9.44331951	-1.15795990	-1.45542918
C	8.96964523	2.21815841	1.61844034
H	8.57635366	2.97655180	0.93203775
H	8.40239352	2.28916661	2.55265661
H	10.01333691	2.47380175	1.83438058
C	9.82365005	0.43070135	-0.72963730
H	10.88039479	0.65980393	-0.55059452
H	9.76460937	-0.56088516	-1.19098302
H	9.44320076	1.15828176	-1.45549556

Supplementary Table 26: Cartesian Coordinates (Å) of the Optimized Geometry for **4b′** Calculated at the M06-2X/6-31+G(d) Level of Theory

H	-2.83638446	-3.22475324	-1.68694569
C	-3.78771477	-3.24609500	-1.16677093
C	-6.33395057	-3.37360755	0.04031992
C	-4.45585925	-2.07691959	-0.82078934
C	-4.37725770	-4.48006221	-0.86918961
C	-5.63367322	-4.54426592	-0.27013739
C	-5.73861304	-2.14860400	-0.23051200
H	-3.85435842	-5.39651244	-1.12575903
H	-6.08316744	-5.51073958	-0.06205462
H	-7.33449298	-3.42763463	0.45980366
C	-6.25688881	-0.77239904	-0.09158259
C	-5.34601004	0.08397384	-0.62610041
H	-5.42176837	1.16233218	-0.58353128
C	-4.15841516	-0.64486118	-1.06352064
C	-2.98911553	-0.09661022	-1.50673695
C	-1.69182382	-0.76062091	-1.54590225
H	-1.51034710	-1.76336642	-1.18439183
C	-0.71424983	0.12701833	-1.88639339
C	-2.73995536	1.29762807	-1.94327878
C	-1.67966115	3.67951178	-2.93268735
C	-1.35240157	1.42719814	-2.17829500
C	-3.59364588	2.35394148	-2.24204376
C	-3.05125341	3.55174865	-2.72000814
C	-0.81830255	2.60772664	-2.67742662
H	-4.66910568	2.26031400	-2.14119814
H	-3.71138273	4.38427560	-2.94424861
H	0.24215291	2.69060430	-2.89778270
H	-1.27878538	4.61092569	-3.32154691
H	4.66904999	-2.25786623	-2.14310751
C	3.59359459	-2.35138513	-2.24412922
C	0.81827347	-2.60470536	-2.67994434
C	2.73989908	-1.29534506	-1.94442108
C	3.05121549	-3.54872148	-2.72328766
C	1.67963506	-3.67625475	-2.93618830
C	1.35235901	-1.42466772	-2.17964039
H	3.71134651	-4.38104361	-2.94828301
H	1.27877081	-4.60728835	-3.32596981
H	-0.24217174	-2.68735791	-2.90043452
C	0.71420827	-0.12476949	-1.88649053
C	1.69177344	0.76251481	-1.54504451
H	1.51028067	1.76488287	-1.18250833
C	2.98907140	0.09846970	-1.50655498
C	4.15840111	0.64622337	-1.06280144
C	5.34593368	-0.08316203	-0.62613992

H	5.42158671	-1.16157291	-0.58466947
C	6.25689495	0.77258062	-0.09075839
C	4.45601443	2.07802207	-0.81869753
C	5.63412974	4.54468955	-0.26560833
C	5.73876793	2.14897710	-0.22832469
C	3.78804086	3.24762489	-1.16357505
C	4.37772899	4.48122692	-0.86476814
C	6.33425056	3.37364082	0.04372217
H	2.83673457	3.22692279	-1.68381270
H	3.85495546	5.39799366	-1.12046257
H	7.33479218	3.42713190	0.46327689
H	6.08374011	5.51090341	-0.05657116
C	7.52372084	0.39943688	0.55214091
C	7.95169511	1.03505689	1.72508425
C	8.31833467	-0.62768538	0.02202749
C	9.13541228	0.64755561	2.34906648
H	7.33495564	1.81259692	2.16820674
C	9.49980246	-1.00338236	0.65120609
H	8.01000663	-1.11369761	-0.90038686
C	9.93939672	-0.37611410	1.82862529
H	9.43024963	1.15525876	3.26527515
H	10.09440147	-1.80081330	0.20692692
C	-7.52374603	-0.40003546	0.55170769
C	-7.95159245	-1.03683216	1.72405908
C	-8.31852358	0.62747808	0.02259900
C	-9.13534783	-0.65009976	2.34844457
H	-7.33472397	-1.81469921	2.16642636
C	-9.50003239	1.00239613	0.65216597
H	-8.01029429	1.11441394	-0.89936108
C	-9.93950155	0.37394110	1.82899873
H	-9.43008362	-1.15871709	3.26417861
H	-10.09476422	1.80015856	0.20866065
C	11.82806218	0.10653038	4.20931518
H	11.01572774	-0.02941525	4.93198758
H	12.76015599	-0.19875764	4.69814198
H	11.90772569	1.17599784	3.98453418
C	-11.82804038	-0.11128688	4.20926129
H	-11.01570663	0.02407387	4.93204410
H	-12.76016494	0.19339362	4.69840826
H	-11.90755678	-1.18054672	3.98344410
Si	11.54996779	-0.91303441	2.65195487
Si	-11.55013215	0.90983139	2.65288439
C	11.43346476	-2.74156227	3.08889285
H	10.61142087	-2.92847444	3.78807788
H	11.25638390	-3.35240559	2.19644131
H	12.36160694	-3.09305563	3.55394994
C	12.97241370	-0.65188558	1.44506198
H	13.06563864	0.40343108	1.16721493
H	13.92370426	-0.97283092	1.88471322
H	12.82055803	-1.22600345	0.52402292
C	-12.97256753	0.64965065	1.44577041
H	-13.92389651	0.97001948	1.88575876
H	-12.82082278	1.22469523	0.52529120
H	-13.06563870	-0.40540662	1.16688916
C	-11.43388413	2.73795001	3.09159755
H	-10.61186078	2.92429917	3.79095694
H	-11.25689694	3.34968476	2.19973835
H	-12.36207230	3.08886012	3.55700309

Supplementary Table 27: Cartesian Coordinates (Å) of the Optimized Geometry for **4c′** Calculated at the M06-2X/6-31+G(d) Level of Theory

H	-0.82072376	3.37508006	-1.09717195
C	0.14445593	3.35524759	-0.60421530
C	2.69230911	3.39848833	0.60283634
C	0.86043329	2.17367120	-0.44362948
C	0.69478454	4.55419013	-0.13781363
C	1.94657766	4.57464836	0.47360049
C	2.15543689	2.21196909	0.12224026
H	0.13531285	5.47757022	-0.25355116
H	2.35136121	5.51189546	0.84384258
H	3.66751206	3.41629513	1.08035126
C	2.70913328	0.84196361	0.09747738
C	1.78309841	0.01982436	-0.47097941
H	1.90309969	-1.04590657	-0.60012992
C	0.58215628	0.76675974	-0.82251374
C	-0.57467209	0.23987371	-1.32576155
C	-1.88250362	0.88225042	-1.32068233
H	-2.08706437	1.84699273	-0.87869600
C	-2.84232780	0.01064786	-1.74358189
C	-0.79599806	-1.11683981	-1.88441184
C	-1.81306842	-3.42104354	-3.08611522
C	-2.18006977	-1.24916415	-2.13923869
C	0.07573630	-2.12881834	-2.27260813
C	-0.44511194	-3.28903909	-2.85659920
C	-2.69270166	-2.38926443	-2.74288801
H	1.14972625	-2.02948408	-2.16530072
H	0.23092208	-4.08605449	-3.15114844
H	-3.75054260	-2.46832852	-2.97664625
H	-2.19685326	-4.32099202	-3.55747273
H	-8.25954900	2.32594439	-1.90066155
C	-7.18419818	2.44313157	-1.97292292
C	-4.40652169	2.77371403	-2.33474001
C	-6.31843139	1.37933369	-1.74256902
C	-6.65378546	3.68332341	-2.34408707
C	-5.28106621	3.84899304	-2.52017668
C	-4.92937805	1.54918399	-1.94067345
H	-7.32398717	4.52057023	-2.51434043
H	-4.88932641	4.81442255	-2.82660216
H	-3.34403752	2.89003250	-2.52863863
C	-4.27421712	0.24016995	-1.74185906
C	-5.24184936	-0.68689245	-1.48893616
H	-5.04872750	-1.71238622	-1.20597809
C	-6.55104993	-0.04926717	-1.42256586
C	-7.71974159	-0.65067476	-1.05230957
C	-8.93221806	0.02222809	-0.59476760
H	-9.02879460	1.09237052	-0.46970663
C	-9.84460060	-0.88763122	-0.16094109
C	-7.99844268	-2.10234698	-0.93234695
C	-9.14931789	-4.62342180	-0.61588852
C	-9.29756981	-2.24033243	-0.39129016
C	-7.29945282	-3.23042567	-1.34678557
C	-7.87603208	-4.49298865	-1.16616771
C	-9.87957578	-3.49158344	-0.23772432
H	-6.33296270	-3.15457146	-1.83259774
H	-7.32894392	-5.37820333	-1.47631760

H	-10.89194588	-3.59391903	0.14236318
H	-9.58807087	-5.60995370	-0.49919943
H	6.52436172	3.54334915	0.70410771
C	7.58769032	3.40332800	0.54447232
C	10.38243566	3.12922409	0.28180808
C	8.17088546	2.14161089	0.57762287
C	8.40025776	4.52404731	0.33756054
C	9.78067287	4.38969691	0.20443416
C	9.57443662	2.01422766	0.46080846
H	7.94785863	5.51041779	0.29852724
H	10.39724665	5.27213893	0.06122308
H	11.46312234	3.03274087	0.22935173
C	9.92420584	0.59284960	0.65697929
C	8.78340061	-0.09468651	0.92845341
H	8.72796712	-1.17010387	1.03022490
C	7.62423599	0.79062151	0.85409285
C	6.31403144	0.41601902	0.93722119
C	5.16733493	1.19546506	0.48450727
H	5.24101140	2.15169470	-0.01436953
C	4.02498497	0.45774986	0.57702179
C	5.76003271	-0.87675969	1.40573016
C	4.13932236	-2.99417544	2.22498110
C	4.36408184	-0.84512221	1.18624171
C	6.32966603	-1.95673557	2.07156806
C	5.51242310	-3.02309545	2.46191663
C	3.54980589	-1.89212780	1.59749341
H	7.38434836	-1.97818554	2.32123937
H	5.95382572	-3.87407585	2.97196987
H	2.47274821	-1.84797255	1.46553063
H	3.51776320	-3.82291280	2.55086122
C	-11.14042413	-0.58510480	0.46082209
C	-11.60425550	-1.31782405	1.56117982
C	-11.92997971	0.47065247	-0.01832750
C	-12.81826130	-0.99680165	2.16456534
H	-10.99324020	-2.12018763	1.96640486
C	-13.14141942	0.77957067	0.58940779
H	-11.59334371	1.03234243	-0.88620188
C	-13.61750818	0.05441344	1.69415257
H	-13.14133538	-1.58002683	3.02453635
H	-13.73073470	1.60198928	0.18546522
C	11.27323677	0.02181085	0.54942805
C	12.15778512	0.43397289	-0.45574062
C	11.69433115	-0.97624294	1.44057130
C	13.42157908	-0.14200164	-0.56615543
H	11.83961931	1.18489773	-1.17427816
C	12.95902747	-1.54105520	1.32322248
H	11.02673585	-1.28832647	2.23991522
C	13.85512448	-1.13856650	0.31930717
H	14.07797065	0.19445040	-1.36621055
H	13.25392123	-2.30863582	2.03785137
C	16.49817031	-1.17427995	-1.25593499
H	17.49298782	-1.62625232	-1.33942884
H	15.97682547	-1.34480012	-2.20447884
H	16.63518113	-0.09411929	-1.13276342
C	-15.59766140	-0.64669168	3.94283373
H	-14.81882991	-0.56339496	4.70908206
H	-16.55392522	-0.39379900	4.41454716
H	-15.65236414	-1.69430222	3.62630085

Si	15.56524278	-1.92642737	0.19537276
Si	-15.26893823	0.50273058	2.48939055
C	-16.63483145	0.32719969	1.20436830
H	-17.60822659	0.60187769	1.62662596
H	-16.45169029	0.97570390	0.34015100
H	-16.70210374	-0.70292945	0.83844376
C	-15.19683477	2.28967285	3.08061738
H	-14.40764659	2.42648954	3.82762372
H	-14.99076161	2.97402396	2.24983495
H	-16.14848358	2.59190045	3.53255842
C	15.36615392	-3.78269733	-0.05503472
H	14.81259313	-4.23734619	0.77433747
H	14.81886415	-3.99977539	-0.97855818
H	16.34240318	-4.27730382	-0.11437856
C	16.50196139	-1.61238418	1.79922582
H	17.49488614	-2.07556533	1.77070162
H	16.63283904	-0.53946139	1.97609504
H	15.96502968	-2.02810986	2.65935632

Supplementary Table 28: Cartesian Coordinates (Å) of the Optimized Geometry for **4a′** Calculated at the ωB97XD/6-31+G(d) Level of Theory

C	0.86932283	-1.77557583	-0.75425175
C	4.30267029	-0.39852028	-0.09394493
C	2.87989914	-0.64204288	-0.37469784
C	2.83043571	-3.22359900	-0.69384546
H	3.90320220	-3.35959399	-0.59102041
C	0.60164508	-0.31883503	-0.65841932
C	0.63623990	-4.13376394	-1.16562188
H	0.00696421	-4.98611007	-1.40510328
C	2.25822761	-1.96367114	-0.58191656
C	2.00402030	-4.31506781	-0.97325478
H	2.43517737	-5.30819445	-1.06179056
C	0.06301008	-2.86156763	-1.07496349
H	-0.99369026	-2.74333032	-1.28509489
C	-0.60167552	0.31871736	-0.65837378
C	-2.87991498	0.64198968	-0.37458231
C	-2.25819320	1.96361079	-0.58169503
C	-0.86929729	1.77547353	-0.75404117
C	-1.91153958	-0.30291057	-0.46552822
H	-2.06838519	-1.35486698	-0.27121200
C	-4.30269201	0.39850046	-0.09383059
C	1.91149141	0.30282850	-0.46558428
H	2.06829745	1.35478352	-0.27121396
C	-2.83034433	3.22357448	-0.69350879
H	-3.90310485	3.35960838	-0.59067299
C	7.03633944	0.12133560	0.47935944
C	-0.06292671	2.86145776	-1.07462371
H	0.99377446	2.74318620	-1.28474300
C	-0.63609897	4.13368810	-1.16516814
H	-0.00678127	4.98602981	-1.40455478
C	-2.00387565	4.31503270	-0.97280646
H	-2.43498663	5.30818767	-1.06124873
C	-7.03637389	-0.12129375	0.47946875
C	-6.34920749	0.93519156	1.09242619
H	-6.86162474	1.57192950	1.81036102
C	-6.31512250	-0.91221703	-0.42857584
H	-6.80551227	-1.74212287	-0.93518882

C	-4.97797552	-0.66149321	-0.71321814
H	-4.44984183	-1.27700310	-1.43713587
C	-5.01053474	1.19645983	0.81265287
H	-4.50134564	2.00891417	1.32436647
C	5.01055414	-1.19651878	0.81247216
H	4.50140171	-2.00902954	1.32413270
C	6.34922035	-0.93522024	1.09224834
H	6.86167030	-1.57199066	1.81013101
C	6.31504714	0.91229782	-0.42861873
H	6.80539940	1.74225926	-0.93517696
C	4.97790620	0.66154411	-0.71326327
H	4.44974020	1.27708705	-1.43712932
C	-9.51519215	0.73944714	2.09617863
H	-9.45054061	1.77053577	1.72894300
H	-10.57016717	0.53151302	2.31131095
H	-8.96772744	0.68315177	3.04447213
C	9.51520560	-0.73942176	2.09598675
H	10.57017659	-0.53146654	2.31111857
H	8.96775115	-0.68320511	3.04429085
H	9.45058348	-1.77048909	1.72868611
Si	-8.85240739	-0.49212344	0.83509513
Si	8.85236357	0.49220706	0.83499014
C	-8.99628073	-2.24596446	1.50969795
H	-8.60124193	-2.97981085	0.79702156
H	-8.43443742	-2.35682243	2.44429689
H	-10.04229429	-2.50758770	1.71057814
C	-9.82917693	-0.35841748	-0.77101137
H	-10.88935588	-0.58820888	-0.60924378
H	-9.76237138	0.65185481	-1.19094869
H	-9.44924566	-1.05646961	-1.52656206
C	8.99618151	2.24600969	1.50970471
H	8.60111000	2.97988821	0.79707950
H	8.43434318	2.35678736	2.44431616
H	10.04218771	2.50765695	1.71059182
C	9.82912084	0.35863894	-0.77113535
H	10.88929333	0.58845781	-0.60936450
H	9.76234672	-0.65160891	-1.19113648
H	9.44915628	1.05672570	-1.52663728

Supplementary Table 29: Cartesian Coordinates (Å) of the Optimized Geometry for **4b′** Calculated at the ωB97XD/6-31+G(d) Level of Theory

H	-2.77146308	-3.25774181	-1.30444046
C	-3.74869134	-3.26758676	-0.83569714
C	-6.34592363	-3.38482678	0.24820610
C	-4.45152938	-2.09487017	-0.58472709
C	-4.33079098	-4.49585227	-0.50688590
C	-5.61345495	-4.55531925	0.03265824
C	-5.75951575	-2.16431884	-0.05661396
H	-3.77879971	-5.41313294	-0.69036240
H	-6.05618993	-5.51875095	0.26875760
H	-7.36342982	-3.43588695	0.62492182
C	-6.30936330	-0.79604233	-0.00685995
C	-5.38715256	0.05248522	-0.52531303
H	-5.49010193	1.12859186	-0.53704320
C	-4.16486878	-0.66786059	-0.87698261
C	-2.99519277	-0.11362960	-1.30295129
C	-1.68617976	-0.75956708	-1.29880380

H	-1.49616007	-1.75061222	-0.91242635
C	-0.71619755	0.12269972	-1.65667639
C	-2.75079885	1.27005108	-1.78013822
C	-1.68955356	3.63323158	-2.81647326
C	-1.36231193	1.40529680	-1.99590369
C	-3.60416581	2.31288785	-2.12286848
C	-3.06311475	3.50013617	-2.62488395
C	-0.82765752	2.57574618	-2.51603288
H	-4.68045898	2.21926438	-2.03688028
H	-3.72617510	4.32079510	-2.88304914
H	0.23674787	2.66095600	-2.71493798
H	-1.28761764	4.55707470	-3.22263069
H	4.68046104	-2.21945034	-2.03684525
C	3.60416593	-2.31306813	-2.12280614
C	0.82764936	-2.57592570	-2.51591160
C	2.75081276	-1.27020964	-1.78010468
C	3.06309719	-3.50033617	-2.62475668
C	1.68953152	-3.63343274	-2.81631356
C	1.36232201	-1.40545620	-1.99584855
H	3.72614737	-4.32101179	-2.88289526
H	1.28758132	-4.55729299	-3.22241813
H	-0.23676100	-2.66113587	-2.71479181
C	0.71621695	-0.12284179	-1.65667669
C	1.68620692	0.75943630	-1.29885544
H	1.49619974	1.75049831	-0.91251728
C	2.99521571	0.11349195	-1.30297660
C	4.16488693	0.66775804	-0.87704016
C	5.38718696	-0.05253675	-0.52532218
H	5.49016279	-1.12864078	-0.53698181
C	6.30937611	0.79604438	-0.00691655
C	4.45150895	2.09478859	-0.58486044
C	5.61336218	4.55530911	0.03236678
C	5.75949289	2.16430583	-0.05675097
C	3.74863108	3.26746719	-0.83589324
C	4.33069481	4.49577035	-0.50716078
C	6.34586695	3.38485200	0.24798486
H	2.77139846	3.25756121	-1.30462758
H	3.77867419	5.41302287	-0.69068940
H	7.36337595	3.43596908	0.62468362
H	6.05607097	5.51876921	0.26839998
C	7.62429674	0.41856031	0.53186884
C	8.10282332	0.96252416	1.72964896
C	8.42068718	-0.52143058	-0.13558906
C	9.33626553	0.57076717	2.24321457
H	7.49187506	1.67440944	2.27841720
C	9.65171158	-0.90325817	0.38433426
H	8.07468532	-0.93656825	-1.07884845
C	10.14278372	-0.36771318	1.58532350
H	9.66831843	1.00829884	3.18218562
H	10.24314282	-1.63222101	-0.16761501
C	-7.62427822	-0.41850195	0.53189824
C	-8.10284596	-0.96244362	1.72967144
C	-8.42062833	0.52150411	-0.13558615
C	-9.33628638	-0.57064426	2.24320971
H	-7.49193724	-1.67435975	2.27844523
C	-9.65164993	0.90337632	0.38431188
H	-8.07459600	0.93662053	-1.07884387
C	-10.14276171	0.36785774	1.58529679

H	-9.66837468	-1.00816405	3.18217377
H	-10.24304789	1.63235285	-0.16765481
C	12.16961890	-0.02918914	3.88267662
H	11.41198445	-0.26306158	4.63991314
H	13.14234839	-0.34034395	4.28198559
H	12.19787432	1.05969700	3.75738239
C	-12.16965604	0.02941388	3.88261084
H	-11.41204462	0.26328949	4.63986931
H	-13.14239644	0.34058119	4.28188370
H	-12.19791633	-1.05947362	3.75732923
Si	11.82251900	-0.90945152	2.25459943
Si	-11.82249524	0.90965210	2.25453295
C	11.79950704	-2.77472260	2.52214076
H	11.03224829	-3.05975421	3.25117360
H	11.58366252	-3.30821195	1.58872064
H	12.76782933	-3.13234896	2.89274689
C	13.15173552	-0.47538985	0.99118175
H	13.18793833	0.60524746	0.81131107
H	14.14312374	-0.79450742	1.33505887
H	12.96025273	-0.96471061	0.02870168
C	-13.15169869	0.47561715	0.99109150
H	-14.14308771	0.79474733	1.33495482
H	-12.96019362	0.96494057	0.02861721
H	-13.18791431	-0.60501861	0.81121429
C	-11.79943348	2.77492513	2.52205604
H	-11.03220272	3.05993783	3.25112568
H	-11.58352181	3.30839702	1.58864152
H	-12.76776260	3.13258918	2.89260801

Supplementary Table 30: Cartesian Coordinates (Å) of the Optimized Geometry for **4c′** Calculated at the ωB97XD/6-31+G(d) Level of Theory

H	-0.85286523	3.39587870	-0.90576992
C	0.11901004	3.36137823	-0.42767453
C	2.67824162	3.37520141	0.74714140
C	0.84771576	2.18101527	-0.33320940
C	0.66208338	4.54225449	0.08736029
C	1.92142517	4.54779396	0.68238523
C	2.14660467	2.20697340	0.21985010
H	0.09099714	5.46380129	0.02223941
H	2.32267614	5.47111246	1.09030715
H	3.66047162	3.38063668	1.21017657
C	2.71592526	0.84814903	0.12479897
C	1.79748105	0.04529037	-0.47263577
H	1.93130611	-1.01116672	-0.65094071
C	0.57971598	0.78973841	-0.77616457
C	-0.57187947	0.26945210	-1.28905734
C	-1.88786622	0.89872213	-1.25590555
H	-2.09876722	1.84812798	-0.78581780
C	-2.84048712	0.03982581	-1.70634308
C	-0.78899263	-1.06834390	-1.89561775
C	-1.80184323	-3.33800597	-3.16494175
C	-2.17172399	-1.19939790	-2.14831002
C	0.08345049	-2.06488982	-2.31913865
C	-0.43385852	-3.20699351	-2.93795103
C	-2.68226023	-2.32236845	-2.78381662
H	1.15737018	-1.96832075	-2.21113187
H	0.24484939	-3.99133826	-3.26014297

H	-3.74163280	-2.40038731	-3.01013637
H	-2.18484391	-4.22455127	-3.66222285
H	-8.26892962	2.33285316	-1.86949331
C	-7.19403156	2.45094372	-1.94356701
C	-4.42105607	2.79185821	-2.30228393
C	-6.32577340	1.39068845	-1.70889496
C	-6.66965204	3.69106282	-2.31930850
C	-5.29794444	3.86227558	-2.49380985
C	-4.93911866	1.56652511	-1.90632062
H	-7.34399076	4.52468688	-2.49273333
H	-4.90904326	4.82847970	-2.80231616
H	-3.35804840	2.91260972	-2.48989281
C	-4.27582521	0.26459362	-1.69884171
C	-5.23476036	-0.66354828	-1.43938369
H	-5.03284808	-1.68623785	-1.15465944
C	-6.55182665	-0.03773138	-1.37949475
C	-7.71321815	-0.64891164	-1.01273583
C	-8.94077819	0.01343515	-0.57598003
H	-9.05530495	1.08316089	-0.46825607
C	-9.84663259	-0.89867328	-0.14304216
C	-7.97979606	-2.10258915	-0.87627675
C	-9.10642617	-4.63046797	-0.52615982
C	-9.28143578	-2.24635161	-0.34813506
C	-7.26557071	-3.23085908	-1.26216313
C	-7.82987433	-4.49535559	-1.06661866
C	-9.85048590	-3.50051187	-0.17586751
H	-6.29353196	-3.15581036	-1.73603193
H	-7.26919828	-5.37959707	-1.35557275
H	-10.86367623	-3.60610645	0.20103921
H	-9.53565935	-5.61963214	-0.39488335
H	6.54345983	3.54047721	0.64928228
C	7.60574352	3.39569345	0.48983438
C	10.39524736	3.11681371	0.21615252
C	8.18655826	2.13364161	0.53435602
C	8.41707311	4.51301515	0.26862560
C	9.79616273	4.37633327	0.12923184
C	9.58769870	2.00521177	0.41280714
H	7.96443256	5.49922680	0.22111369
H	10.41323944	5.25643919	-0.02754613
H	11.47511027	3.01751039	0.15437886
C	9.93984758	0.58701892	0.61673431
C	8.80297519	-0.09821137	0.89457502
H	8.75238241	-1.17235248	1.00628482
C	7.63941814	0.78371012	0.82112499
C	6.33355855	0.40639115	0.91467558
C	5.17976081	1.18655267	0.47622161
H	5.24540011	2.14719582	-0.01412164
C	4.04015687	0.45359703	0.57966628
C	5.77962004	-0.88730812	1.38401356
C	4.16026326	-3.00688284	2.20124487
C	4.38338315	-0.85090735	1.17885096
C	6.35052318	-1.97486793	2.03551393
C	5.53478571	-3.04084376	2.42613141
C	3.57044212	-1.89935215	1.58717584
H	7.40748139	-2.00497052	2.27336929
H	5.97917278	-3.89700473	2.92541692
H	2.49295056	-1.85273006	1.45956982
H	3.53881673	-3.83693079	2.52481496

C	-11.15685651	-0.60082814	0.45365472
C	-11.61015757	-1.28677509	1.58663885
C	-11.97306712	0.40181094	-0.08659563
C	-12.83865076	-0.97245462	2.16146396
H	-10.98327166	-2.05073919	2.03885236
C	-13.19890770	0.70578957	0.49361226
H	-11.64694534	0.92875876	-0.97978146
C	-13.66483885	0.02741901	1.63076508
H	-13.15100165	-1.52125786	3.04723867
H	-13.80649520	1.48756425	0.04033178
C	11.29051975	0.01528241	0.51348474
C	12.15642910	0.38038992	-0.52401366
C	11.73247229	-0.93414934	1.44437510
C	13.42094799	-0.19316331	-0.62688232
H	11.82642459	1.09589709	-1.27254562
C	12.99789168	-1.49829940	1.33407690
H	11.08158692	-1.21213577	2.26953159
C	13.87560321	-1.14295405	0.29791082
H	14.06045806	0.10900845	-1.45337234
H	13.30625644	-2.22810386	2.08114123
C	16.49908939	-1.25135525	-1.31594949
H	17.49567959	-1.70269156	-1.39055728
H	15.96567861	-1.47372810	-2.24769164
H	16.63307749	-0.16491105	-1.25380253
C	-15.66496396	-0.62675106	3.88261098
H	-14.90258517	-0.49261618	4.65908228
H	-16.63610676	-0.37971728	4.32795345
H	-15.68749849	-1.68859702	3.61033860
Si	15.58852510	-1.92827821	0.18691177
Si	-15.33700398	0.46927392	2.38706826
C	-16.68038181	0.21092467	1.09091764
H	-17.66758938	0.47869216	1.48669184
H	-16.50017415	0.82798816	0.20253952
H	-16.71918442	-0.83484426	0.76488836
C	-15.31091792	2.28023665	2.90769669
H	-14.53633880	2.46347444	3.66125874
H	-15.10388134	2.93644348	2.05387715
H	-16.27537076	2.58289895	3.33330562
C	15.39432287	-3.79664426	0.03591784
H	14.85259544	-4.21114125	0.89452299
H	14.83545809	-4.06392212	-0.86831905
H	16.37141125	-4.29262360	-0.01123999
C	16.54785386	-1.52421792	1.75787726
H	17.54394616	-1.98297160	1.74165642
H	16.67594444	-0.44196543	1.87516026
H	16.02600122	-1.89402310	2.64859338

Supplementary Table 31: Cartesian Coordinates (Å) of the Optimized Geometry for [**4a**']$^{\bullet-}$ Calculated at the UPBE0/6-31+G(d) Level of Theory

C	0.88040965	-1.75029165	-0.87053598
C	4.30001101	-0.45048567	-0.07484765
C	2.90571811	-0.70784089	-0.38818110
C	2.80677244	-3.25889955	-0.93002858
H	3.87575636	-3.44342989	-0.85606654
C	0.62793924	-0.32991893	-0.66893443
C	0.58300413	-4.07209915	-1.46537003

H	-0.06816500	-4.88934759	-1.76976419
C	2.28207869	-1.98281281	-0.69936447
C	1.95523460	-4.29979372	-1.29726407
H	2.36458798	-5.29314856	-1.47229816
C	0.04809034	-2.79995671	-1.26948382
H	-1.00777031	-2.62577825	-1.45700491
C	-0.62794988	0.33018501	-0.66896326
C	-2.90573620	0.70801124	-0.38818680
C	-2.28215097	1.98301923	-0.69935129
C	-0.88047837	1.75055023	-0.87059021
C	-1.89543129	-0.25483852	-0.41052653
H	-2.03868532	-1.29275478	-0.13163036
C	-4.30000800	0.45056844	-0.07482194
C	1.89542669	0.25502928	-0.41033284
H	2.03871424	1.29290388	-0.13130912
C	-2.80690014	3.25908961	-0.92999143
H	-3.87588795	3.44357968	-0.85600906
C	7.04239831	0.11633541	0.57067810
C	-0.04822333	2.80025194	-1.26958573
H	1.00763059	2.62612330	-1.45717282
C	-0.58319045	4.07237861	-1.46542411
H	0.06793364	4.88965438	-1.76984141
C	-1.95541876	4.30002144	-1.29724771
H	-2.36481785	5.29336177	-1.47225730
C	-7.04234052	-0.11646945	0.57075916
C	-6.45664883	1.12884904	0.85204911
H	-7.04590347	1.90570969	1.33852420
C	-6.20885809	-1.07436010	-0.03823181
H	-6.60538548	-2.06029575	-0.28455110
C	-4.88613731	-0.80348334	-0.35470078
H	-4.28556846	-1.56273830	-0.85037143
C	-5.12958516	1.40769889	0.54374417
H	-4.71025505	2.36997349	0.82298199
C	5.12957628	-1.40774662	0.54352935
H	4.71021893	-2.37005337	0.82262342
C	6.45666720	-1.12900044	0.85181055
H	7.04591405	-1.90596178	1.33813417
C	6.20892948	1.07435147	-0.03813514
H	6.60548920	2.06030471	-0.28433181
C	4.88618187	0.80357895	-0.35458150
H	4.28562323	1.56293089	-0.85011570
C	-9.63992717	0.95306898	1.85677759
H	-9.63583176	1.85752782	1.23629926
H	-10.68514680	0.72212257	2.09897202
H	-9.12554027	1.18949335	2.79621022
C	9.63999348	-0.95349662	1.85643483
H	10.68523591	-0.72263276	2.09860966
H	9.12563949	-1.19001835	2.79586099
H	9.63582472	-1.85787441	1.23583885
Si	-8.83024925	-0.50633801	0.96857157
Si	8.83034451	0.50606577	0.96845772
C	-8.93806721	-2.03478348	2.07948962
H	-8.46743337	-2.90455683	1.60460927
H	-8.42209209	-1.86403689	3.03199491
H	-9.98078675	-2.29843183	2.29953715
C	-9.79709588	-0.86503563	-0.61906486
H	-10.84200901	-1.12453910	-0.40427388
H	-9.79303312	0.00613026	-1.28525534

H	-9.35171882	-1.70119958	-1.17225926
C	8.93828740	2.03436060	2.07957067
H	8.46767251	2.90421862	1.60482673
H	8.42234943	1.86351375	3.03207810
H	9.98102996	2.29793059	2.29960315
C	9.79713456	0.86492404	-0.61917686
H	10.84207029	1.12434888	-0.40440087
H	9.79299852	-0.00615470	-1.28548085
H	9.35177201	1.70118165	-1.17224129

Supplementary Table 32: Cartesian Coordinates (Å) of the Optimized Geometry for $[\mathbf{4b'}]^{\bullet-}$ Calculated at the UPBE0/6-31+G(d) Level of Theory

H	3.00280854	3.10291418	-1.93639525
C	3.99138572	3.16660161	-1.49180702
C	6.61029227	3.35777431	-0.47001239
C	4.62487521	2.02822859	-0.99067079
C	4.64997472	4.39572754	-1.45238044
C	5.95003819	4.49103265	-0.94541611
C	5.95183877	2.12687968	-0.47714167
H	4.15170434	5.28392713	-1.83468317
H	6.45813619	5.45310276	-0.93670032
H	7.63816447	3.43791797	-0.12490686
C	6.39294690	0.78329367	-0.12254666
C	5.36969125	-0.08796348	-0.45821583
H	5.37440542	-1.14712061	-0.22978336
C	4.23796906	0.61970687	-0.96845867
C	2.98855999	0.06889994	-1.30549990
C	1.74796963	0.74244652	-1.35190349
H	1.61219054	1.77329823	-1.05501689
C	0.69119458	-0.14852028	-1.60082563
C	2.70232693	-1.34070580	-1.62288937
C	1.60436252	-3.78396087	-2.40337037
C	1.30051079	-1.47282077	-1.79967702
C	3.53968541	-2.42702618	-1.86283748
C	2.98303024	-3.65392781	-2.23414694
C	0.75872751	-2.68924792	-2.20565986
H	4.61851754	-2.32569138	-1.79616884
H	3.63383885	-4.50751293	-2.41109444
H	-0.30496068	-2.78825290	-2.39935593
H	1.18339588	-4.73869787	-2.71097129
H	-4.61851779	2.32575269	-1.79608852
C	-3.53968568	2.42708986	-1.86275388
C	-0.75872781	2.68932339	-2.20556770
C	-2.70232711	1.34076131	-1.62284308
C	-2.98303058	3.65400420	-2.23402144
C	-1.60436289	3.78404308	-2.40324061
C	-1.30051099	1.47288232	-1.79962642
H	-3.63383923	4.50759538	-2.41093962
H	-1.18339632	4.73879062	-2.71080891
H	0.30496035	2.78833506	-2.39926052
C	-0.69119481	0.14857501	-1.60082050
C	-1.74796986	-0.74240030	-1.35192869
H	-1.61219082	-1.77326220	-1.05507745
C	-2.98856014	-0.06885526	-1.30550183
C	-4.23796920	-0.61967359	-0.96847900
C	-5.36969088	0.08797935	-0.45821118

H	-5.37440463	1.14712861	-0.22974221
C	-6.39294653	-0.78328924	-0.12257133
C	-4.62487553	-2.02819447	-0.99073944
C	-5.95003840	-4.49100001	-0.94556848
C	-5.95183872	-2.12686311	-0.47721276
C	-3.99138637	-3.16655020	-1.49191532
C	-4.64997533	-4.39567750	-1.45253053
C	-6.61029217	-3.35775801	-0.47012535
H	-3.00280953	-3.10284735	-1.93650210
H	-4.15170524	-5.28386396	-1.83486413
H	-7.63816415	-3.43791368	-0.12502192
H	-6.45813646	-5.45307037	-0.93688536
C	-7.65321818	-0.40302329	0.50339988
C	-8.31169106	-1.23393872	1.42725507
C	-8.25069936	0.84154937	0.22344978
C	-9.50140904	-0.84038835	2.03229115
H	-7.86130396	-2.18378537	1.70187067
C	-9.43525385	1.22757631	0.83513999
H	-7.77927408	1.49848599	-0.50401259
C	-10.10120182	0.39786514	1.75549871
H	-9.96487746	-1.51832426	2.74778228
H	-9.85579206	2.20004796	0.57742362
C	7.65321903	0.40300645	0.50341078
C	8.31169153	1.23388988	1.42729501
C	8.25070095	-0.84155594	0.22341674
C	9.50140989	0.84031896	2.03231705
H	7.86130396	2.18372673	1.70194379
C	9.43525575	-1.22760369	0.83509319
H	7.77927588	-1.49846715	-0.50406875
C	10.10120325	-0.39792453	1.75548104
H	9.96487820	1.51823016	2.74783163
H	9.85579471	-2.20006591	0.57734240
C	-12.32936089	-0.38801849	3.74537465
H	-11.60560390	-0.58893497	4.54458171
H	-13.26619222	-0.06994682	4.22003954
H	-12.52649201	-1.33361439	3.22582821
C	12.32937910	0.38790516	3.74535930
H	11.60563295	0.58880731	4.54457972
H	13.26621571	0.06982178	4.22000599
H	12.52650566	1.33351098	3.22582919
Si	-11.70856316	0.94478926	2.55952621
Si	11.70856301	-0.94487743	2.55949224
C	-11.43663702	2.55102632	3.51923578
H	-10.69795987	2.41197919	4.31765148
H	-11.06408110	3.34799503	2.86398147
H	-12.36903955	2.90512931	3.97748875
C	-13.02117997	1.25381006	1.23353200
H	-13.23008328	0.33911601	0.66588485
H	-13.96331170	1.60037350	1.67744269
H	-12.69056744	2.01573653	0.51694032
C	13.02117034	-1.25388986	1.23348675
H	13.96329995	-1.60047146	1.67738766
H	12.69054675	-2.01580035	0.51688318
H	13.23008158	-0.33918829	0.66585463
C	11.43662596	-2.55112709	3.51917773
H	10.69795257	-2.41208581	4.31759793
H	11.06406068	-3.34808199	2.86391196
H	12.36902680	-2.90524564	3.97742224

Supplementary Table 33: Cartesian Coordinates (Å) of the Optimized Geometry for [**4a**']$^{2-}$ Calculated at the PBE0/6-31+G(d) Level of Theory

C	0.91687013	-1.70790278	-1.02734812
C	4.29767609	-0.47972278	-0.04133945
C	2.93620588	-0.74015250	-0.39140103
C	2.82474031	-3.23391746	-1.19689160
H	3.88725679	-3.45544612	-1.13140780
C	0.65025262	-0.33087616	-0.68528837
C	0.59338725	-3.96517568	-1.83847276
H	-0.06347732	-4.74417094	-2.22538394
C	2.32582345	-1.96680901	-0.84355711
C	1.96723013	-4.22128990	-1.67696714
H	2.37118124	-5.19891499	-1.94172307
C	0.07584223	-2.71171517	-1.52480973
H	-0.97902037	-2.49697476	-1.68567131
C	-0.65026337	0.33093151	-0.68525142
C	-2.93621193	0.74018418	-0.39129383
C	-2.32583826	1.96687484	-0.84336900
C	-0.91688784	1.70798353	-1.02720342
C	-1.87798847	-0.22118387	-0.33178319
H	-1.99391394	-1.23692014	0.03537354
C	-4.29767610	0.47972700	-0.04122931
C	1.87798460	0.22121247	-0.33180222
H	1.99391757	1.23692153	0.03542750
C	-2.82476211	3.23400935	-1.19660001
H	-3.88727761	3.45553243	-1.13108113
C	7.05550751	0.11766812	0.68156770
C	-0.07586920	2.71183377	-1.52460421
H	0.97899083	2.49710616	-1.68549978
C	-0.59342059	3.96531732	-1.83816472
H	0.06343675	4.74434206	-2.22502882
C	-1.96726092	4.22141835	-1.67661642
H	-2.37121735	5.19906293	-1.94129226
C	-7.05549565	-0.11772040	0.68167655
C	-6.56405647	1.20272356	0.59687671
H	-7.22945119	2.03887533	0.81929339
C	-6.10683928	-1.13154139	0.40410990
H	-6.40950004	-2.18054372	0.44956130
C	-4.79785908	-0.85395848	0.06075312
H	-4.12598651	-1.67898246	-0.16258917
C	-5.25264608	1.49418594	0.25707350
H	-4.93387817	2.53106795	0.26298106
C	5.25264853	-1.49420470	0.25687730
H	4.93387844	-2.53108641	0.26271692
C	6.56406446	-1.20276889	0.59668183
H	7.22946061	-2.03893764	0.81903028
C	6.10684907	1.13151059	0.40408655
H	6.40951258	2.18050911	0.44960762
C	4.79786317	0.85395448	0.06072972
H	4.12598870	1.67899546	-0.16254414
C	-9.80394460	1.05817455	1.46206132
H	-9.82751675	1.71283580	0.58202960
H	-10.84114344	0.81129182	1.72550334
H	-9.37400264	1.63292779	2.29178693
C	9.80395825	-1.05828818	1.46185288

H	10.84116058	-0.81142666	1.72530070
H	9.37401999	-1.63308822	2.29154798
H	9.82751955	-1.71289744	0.58178218
Si	-8.80531405	-0.51954421	1.12393107
Si	8.80533342	0.51945616	1.12382504
C	-8.91813485	-1.61363104	2.67501888
H	-8.34522184	-2.54003605	2.54056430
H	-8.49645456	-1.09600081	3.54549544
H	-9.95604321	-1.89016506	2.90781859
C	-9.69407465	-1.47295560	-0.26116757
H	-10.71895239	-1.75006908	0.02318672
H	-9.74204959	-0.87029367	-1.17661248
H	-9.15235495	-2.39490169	-0.50834143
C	8.91817740	1.61345230	2.67497510
H	8.34526884	2.53986878	2.54058071
H	8.49650325	1.09577402	3.54542606
H	9.95608999	1.88996633	2.90777972
C	9.69408539	1.47294209	-0.26122790
H	10.71896795	1.75003257	0.02313141
H	9.74204668	0.87033273	-1.17670813
H	9.15236881	2.39490586	-0.50834269

Supplementary Table 34: Cartesian Coordinates (Å) of the Optimized Geometry for [**4b'**]$^{2-}$ Calculated at the PBE0/6-31+G(d) Level of Theory

H	3.10785364	2.95382629	-2.12748306
C	4.12165924	3.04677293	-1.74578129
C	6.78906068	3.27757075	-0.86739072
C	4.74213373	1.95609097	-1.12320807
C	4.81532973	4.24209996	-1.90661434
C	6.14602163	4.35482314	-1.47065891
C	6.10057991	2.07085195	-0.66567590
H	4.32813721	5.08915188	-2.38713582
H	6.68632885	5.28926103	-1.61791647
H	7.83459242	3.37651816	-0.58505558
C	6.49911488	0.78129567	-0.14632522
C	5.39916965	-0.07870660	-0.32970737
H	5.35959402	-1.09992316	0.03537553
C	4.30044956	0.59735419	-0.89632423
C	2.99496532	0.05767262	-1.14170129
C	1.78225893	0.73459686	-1.16549718
H	1.67501748	1.77860467	-0.90392271
C	0.67954895	-0.15666635	-1.36639679
C	2.68524371	-1.35662168	-1.40431965
C	1.56918419	-3.82887872	-2.05449947
C	1.27613741	-1.49240569	-1.53058542
C	3.51721261	-2.45276102	-1.61917999
C	2.95288091	-3.69374251	-1.92737626
C	0.72826719	-2.72619869	-1.87586948
H	4.59692020	-2.33983514	-1.57817326
H	3.59948669	-4.55517436	-2.08676532
H	-0.33952904	-2.83832871	-2.03359016
H	1.13795125	-4.79493497	-2.31065947
H	-4.59691647	2.33989382	-1.57807824
C	-3.51720875	2.45282009	-1.61908135
C	-0.72826298	2.72626465	-1.87576072
C	-2.68524108	1.35667128	-1.40426486

C	-2.95287564	3.69381307	-1.92722885
C	-1.56917875	3.82895272	-2.05434699
C	-1.27613473	1.49245865	-1.53052585
H	-3.59948044	4.55525201	-2.08658351
H	-1.13794475	4.79501866	-2.31046868
H	0.33953354	2.83839949	-2.03347655
C	-0.67954754	0.15671242	-1.36639027
C	-1.78225823	-0.73455728	-1.16552300
H	-1.67501778	-1.77857485	-0.90398744
C	-2.99496411	-0.05763291	-1.14170198
C	-4.30044908	-0.59732238	-0.89634616
C	-5.39916854	0.07871841	-0.32970440
H	-5.35959179	1.09992140	0.03541643
C	-6.49911451	-0.78128969	-0.14635409
C	-4.74213506	-1.95604989	-1.12328221
C	-6.14602586	-4.35476733	-1.47082341
C	-6.10058107	-2.07082682	-0.66575346
C	-4.12166235	-3.04670876	-1.74589767
C	-4.81533423	-4.24202892	-1.90677571
C	-6.78906322	-3.27753721	-0.86751370
H	-3.10785700	-2.95374873	-2.12759680
H	-4.32814301	-5.08906302	-2.38732992
H	-7.83459496	-3.37649414	-0.58518180
H	-6.68633420	-5.28919899	-1.61811622
C	-7.74896725	-0.40506558	0.47076687
C	-8.60944751	-1.33220923	1.10548609
C	-8.17771692	0.94660280	0.49760019
C	-9.80133962	-0.93962954	1.70086863
H	-8.30832533	-2.37336418	1.16717974
C	-9.36417423	1.32908125	1.10048823
H	-7.56262505	1.69859185	0.00886521
C	-10.22766762	0.40095704	1.72005919
H	-10.41130943	-1.70644962	2.17940374
H	-9.63310560	2.38686352	1.07700591
C	7.74896820	0.40504959	0.47078132
C	8.60944777	1.33217016	1.10553506
C	8.17771915	-0.94661940	0.49756348
C	9.80134047	0.93956915	1.70090250
H	8.30832501	2.37332264	1.16726740
C	9.36417709	-1.32911940	1.10043665
H	7.56262817	-1.69859047	0.00879977
C	10.22766963	-0.40101775	1.72004256
H	10.41131027	1.70637194	2.17946531
H	9.63310988	-2.38690041	1.07691366
C	-12.72468874	-0.53338085	3.28214363
H	-12.11374566	-1.01387049	4.05619417
H	-13.66357824	-0.20988979	3.75018620
H	-12.97068228	-1.29467611	2.53168424
C	12.72473726	0.53327819	3.28207777
H	12.11382472	1.01378794	4.05613993
H	13.66363190	0.20977182	3.75009950
H	12.97072673	1.29456116	2.53160460
Si	-11.82608047	0.94230771	2.50695069
Si	11.82607824	-0.94239881	2.50692187
C	-11.52009267	2.23056332	3.86428496
H	-10.89794538	1.81281510	4.66514481
H	-10.99152529	3.10530440	3.46513368
H	-12.46096595	2.58151785	4.30923116

C	-12.98340333	1.73609030	1.23050332
H	-13.24332525	1.01972285	0.44160215
H	-13.91496686	2.09039980	1.69215193
H	-12.50206868	2.59428262	0.74491289
C	12.98335814	-1.73623508	1.23046934
H	13.91491706	-2.09056672	1.69211014
H	12.50199095	-2.59441988	0.74489797
H	13.24329117	-1.01988700	0.44155428
C	11.52007732	-2.23062280	3.86428356
H	10.89795040	-1.81284497	4.66514382
H	10.99148296	-3.10535761	3.46515424
H	12.46094764	-2.58159251	4.30922425

Supplementary Table 35: Cartesian Coordinates (Å) of the Optimized Geometry for fullerene C_{60} Calculated at the PBE0/6-31+G(d) Level of Theory

C	-1.17140622	0.38061295	3.31654470
C	-2.59124824	-1.57363193	1.82363446
C	-0.72396886	-0.99645765	3.31654470
C	-2.29735265	0.74645513	2.58486037
C	-3.02132151	-0.25000253	1.82363446
C	-1.41984202	-1.95424489	2.58486037
C	0.72396886	-0.99645765	3.31654470
C	1.17140622	0.38061295	3.31654470
C	-0.00000000	1.23168939	3.31654470
C	-0.00000000	2.41557952	2.58486037
C	-1.17140622	2.79619248	1.82363446
C	-0.72396886	3.41203717	0.59194506
C	1.17140622	2.79619248	1.82363446
C	2.59124824	2.33485784	-0.59194506
C	2.29735265	1.97814452	1.82363446
C	0.72396886	3.41203717	0.59194506
C	1.41984202	3.18593428	-0.59194506
C	3.02132151	1.74291277	0.59194506
C	-0.72396886	-3.41203717	-0.59194506
C	-3.02132151	-1.74291277	-0.59194506
C	-1.17140622	-2.79619248	-1.82363446
C	-1.41984202	-3.18593428	0.59194506
C	-2.59124824	-2.33485784	0.59194506
C	-2.29735265	-1.97814452	-1.82363446
C	0.00000000	-2.41557952	-2.58486037
C	1.17140622	-2.79619248	-1.82363446
C	0.72396886	-3.41203717	-0.59194506
C	1.41984202	-3.18593428	0.59194506
C	0.69587316	-2.95070253	1.82363446
C	1.41984202	-1.95424489	2.58486037
C	2.59124824	-2.33485784	0.59194506
C	3.46875887	0.36584217	0.59194506
C	3.02132151	-1.74291277	-0.59194506
C	2.59124824	-1.57363193	1.82363446
C	3.02132151	-0.25000253	1.82363446
C	3.46875887	-0.36584217	-0.59194506
C	-2.59124824	1.57363193	-1.82363446
C	-3.46875887	0.36584217	0.59194506
C	-2.59124824	2.33485784	-0.59194506
C	-3.02132151	0.25000253	-1.82363446
C	-3.46875887	-0.36584217	-0.59194506

C	-3.02132151	1.74291277	0.59194506
C	-1.41984202	3.18593428	-0.59194506
C	-0.69587316	2.95070253	-1.82363446
C	-1.41984202	1.95424489	-2.58486037
C	-0.72396886	0.99645765	-3.31654470
C	-1.17140622	-0.38061295	-3.31654470
C	0.00000000	-1.23168939	-3.31654470
C	0.72396886	0.99645765	-3.31654470
C	3.02132151	0.25000253	-1.82363446
C	1.41984202	1.95424489	-2.58486037
C	1.17140622	-0.38061295	-3.31654470
C	2.29735265	-0.74645513	-2.58486037
C	2.59124824	1.57363193	-1.82363446
C	0.69587316	2.95070253	-1.82363446
C	2.29735265	-1.97814452	-1.82363446
C	2.29735265	0.74645513	2.58486037
C	-0.69587316	-2.95070253	1.82363446
C	-2.29735265	1.97814452	1.82363446
C	-2.29735265	-0.74645513	-2.58486037

9.2 NMR Spectra of Newly Synthesized Compounds

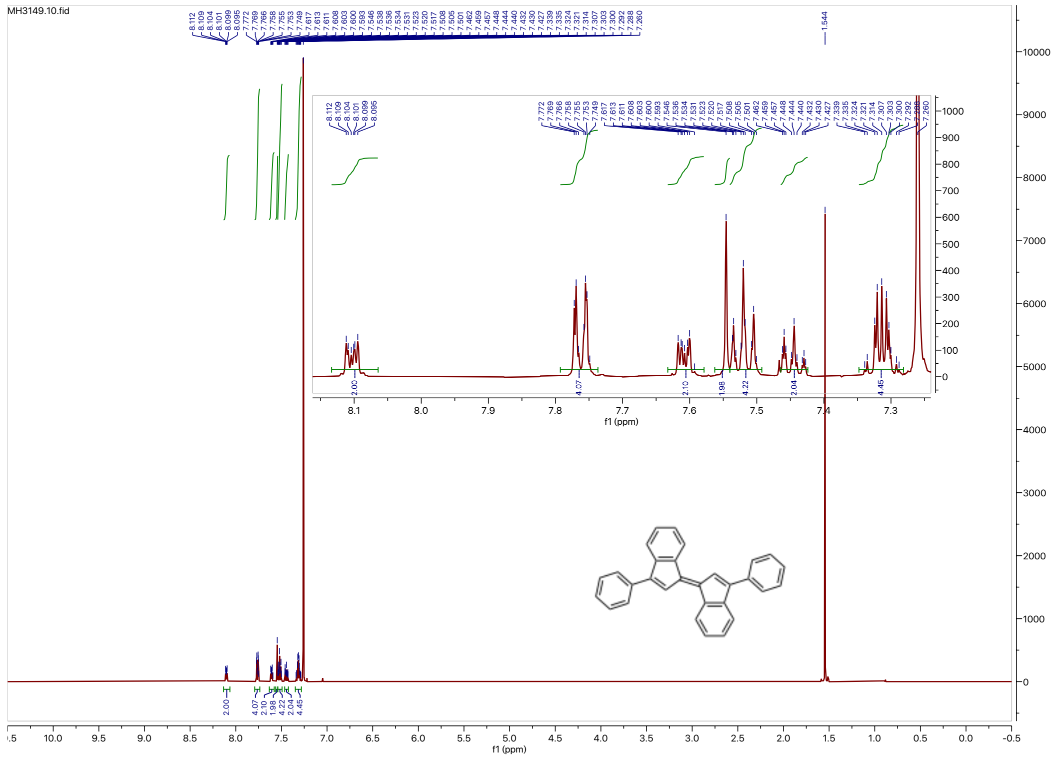

Supplementary Fig. 17: ^{1}H NMR spectrum of **3a** (500 MHz, $CDCl_3$).

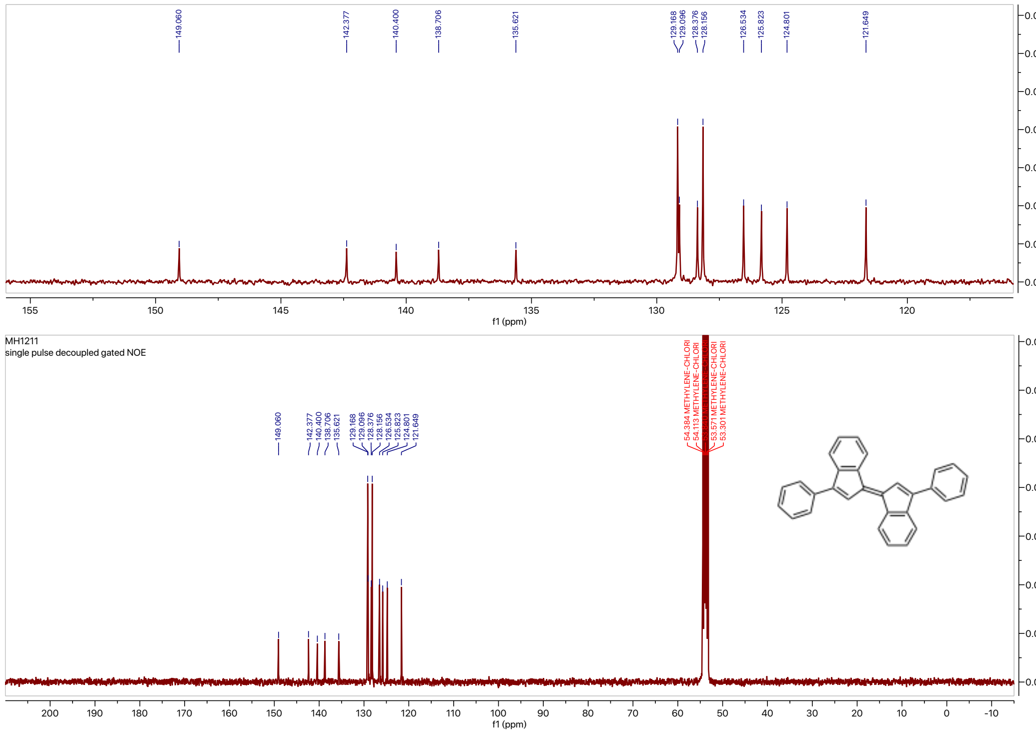

Supplementary Fig. 18: $^{13}C\{^{1}H\}$ NMR spectrum of **3a** (100 MHz, CD_2Cl_2).

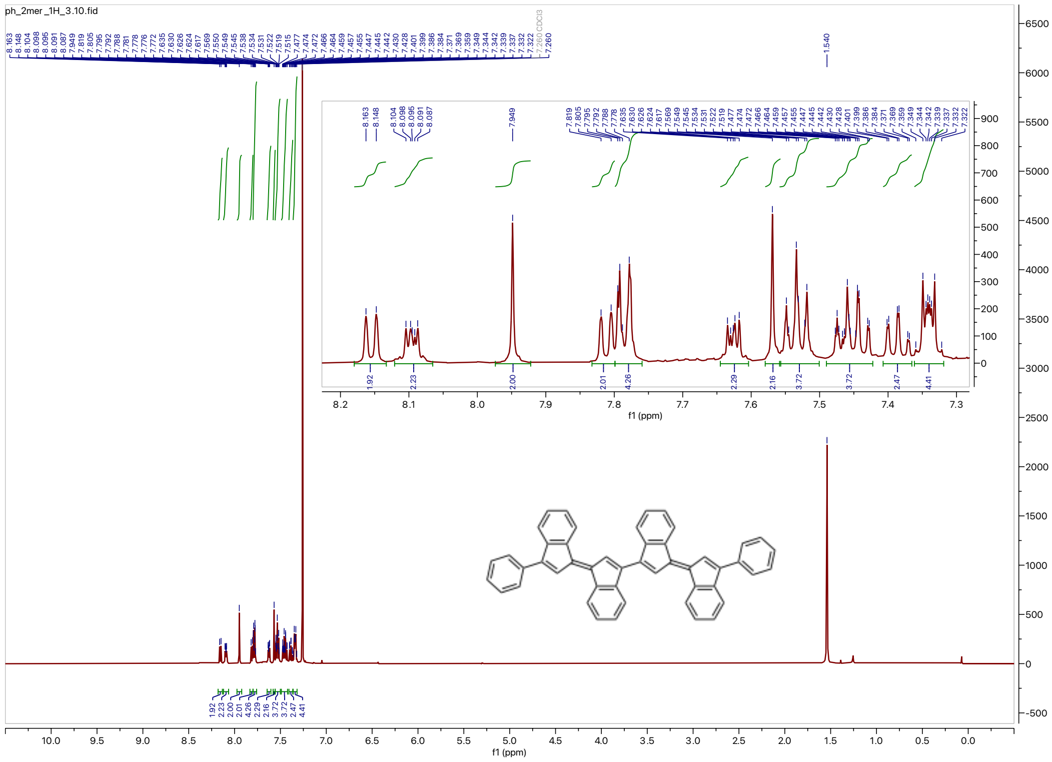

Supplementary Fig. 19: ^{1}H NMR spectrum of **3b** (500 MHz, $CDCl_3$).

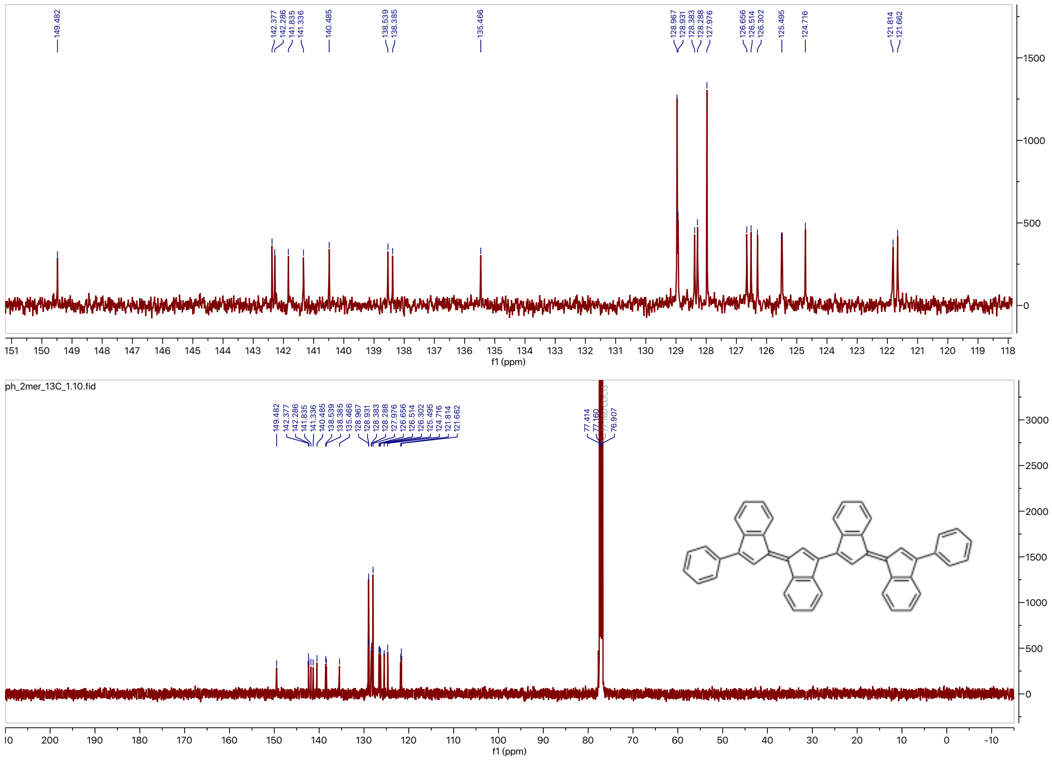

Supplementary Fig. 20: ^{13}C{^{1}H} NMR spectrum of **3b** (125 MHz, $CDCl_3$).

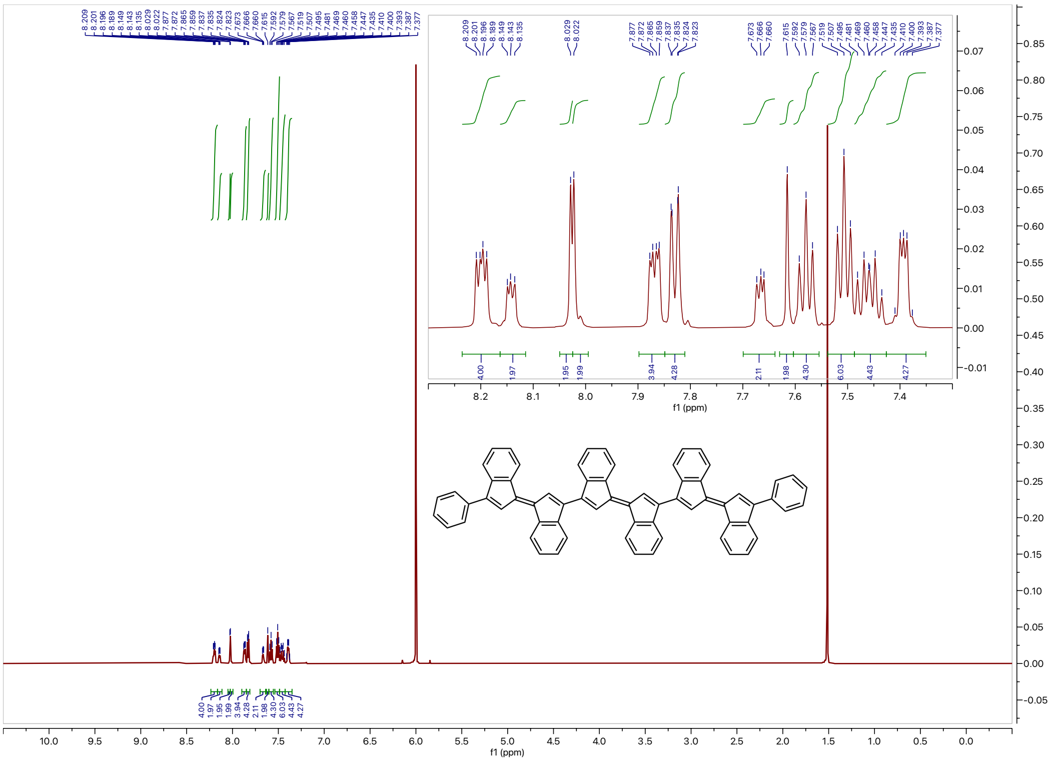

Supplementary Fig. 21: ^{1}H NMR spectrum of **3c** (600 MHz, TCE-d_2, 80 °C).

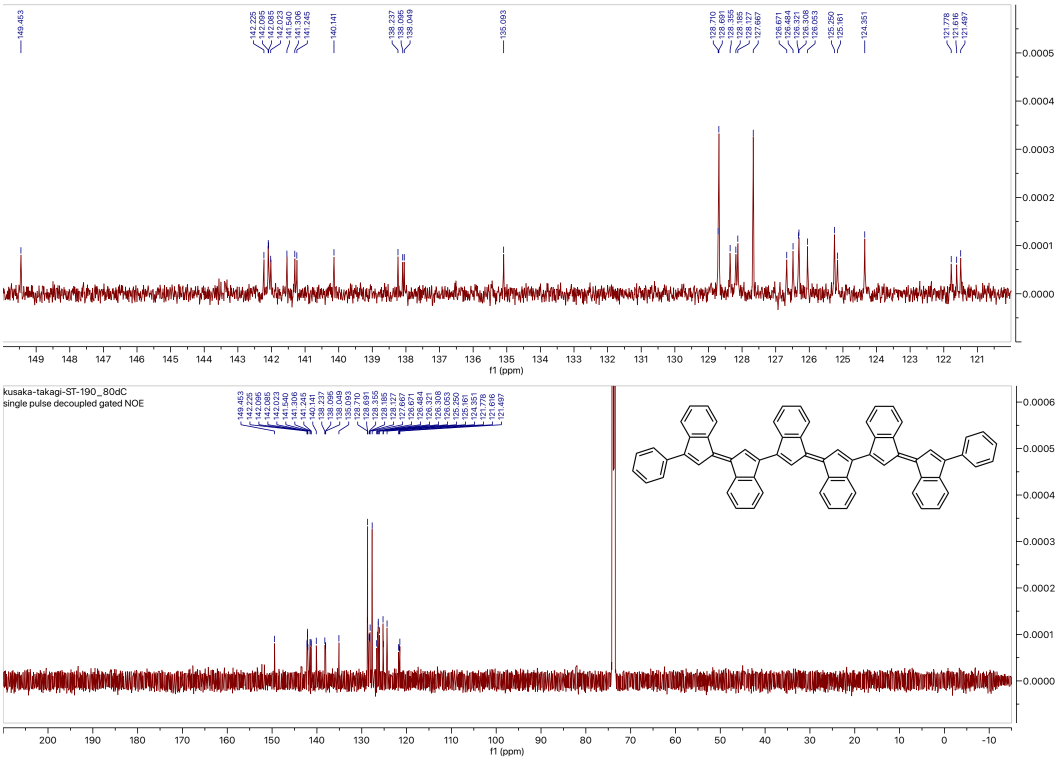

Supplementary Fig. 22: ^{13}C{^{1}H} NMR spectrum of **3c** (150 MHz, TCE-d_2, 80 °C).

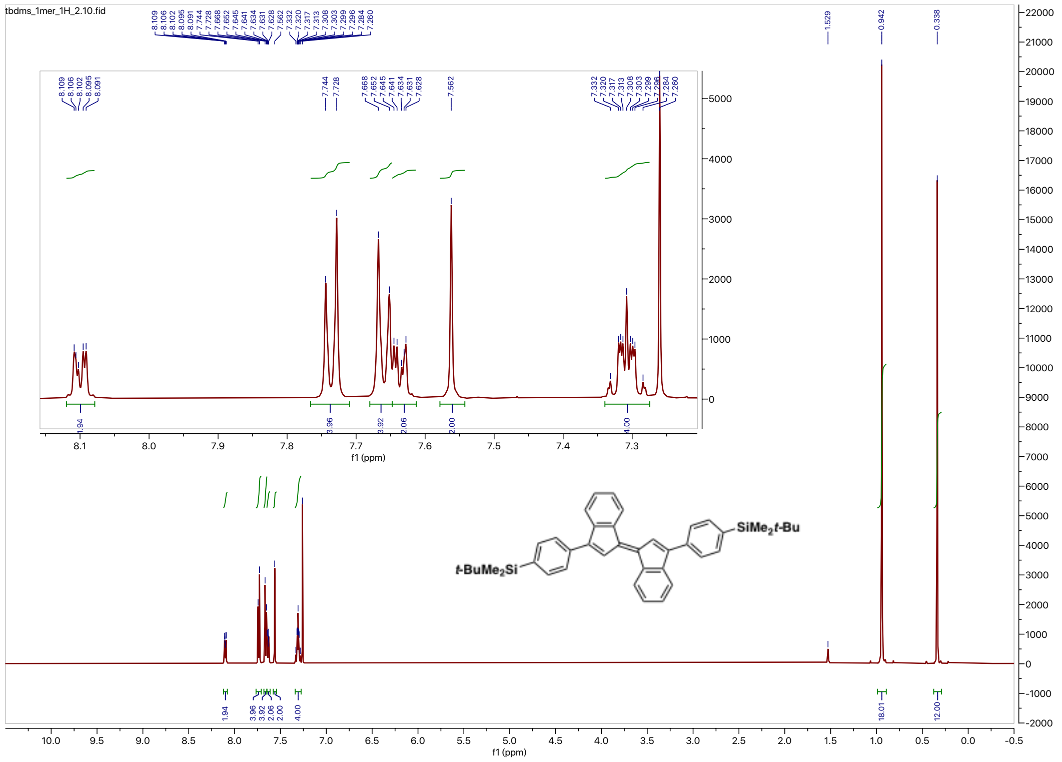

Supplementary Fig. 23: ^{1}H NMR spectrum of **4a** (500 MHz, $CDCl_3$).

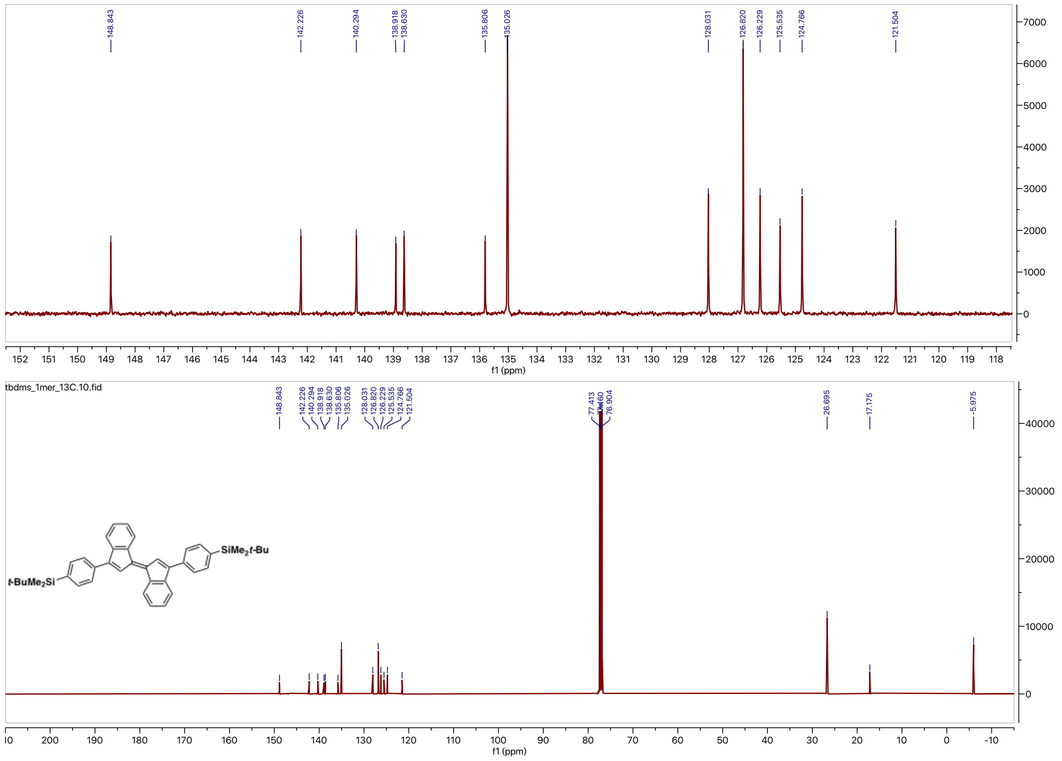

Supplementary Fig. 24: $^{13}C\{^{1}H\}$ NMR spectrum of **4a** (125 MHz, $CDCl_3$).

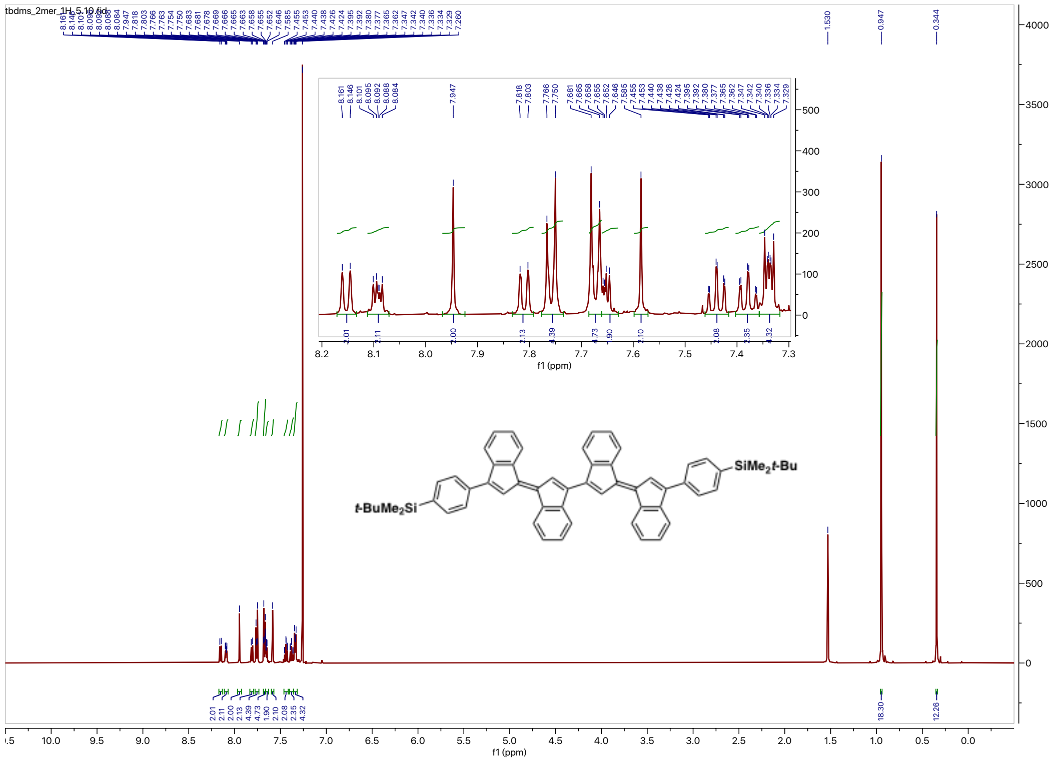

Supplementary Fig. 25: ^{1}H NMR spectrum of **4b** (500 MHz, $CDCl_3$).

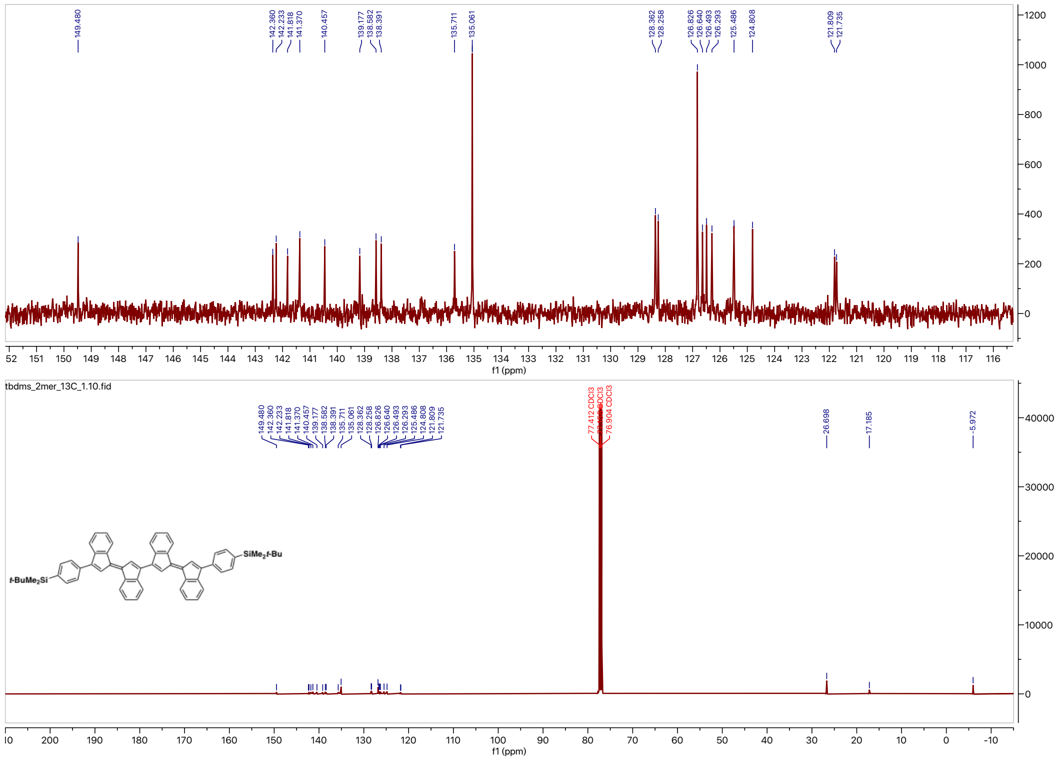

Supplementary Fig. 26: $^{13}C\{^{1}H\}$ NMR spectrum of **4b** (125 MHz, $CDCl_3$).

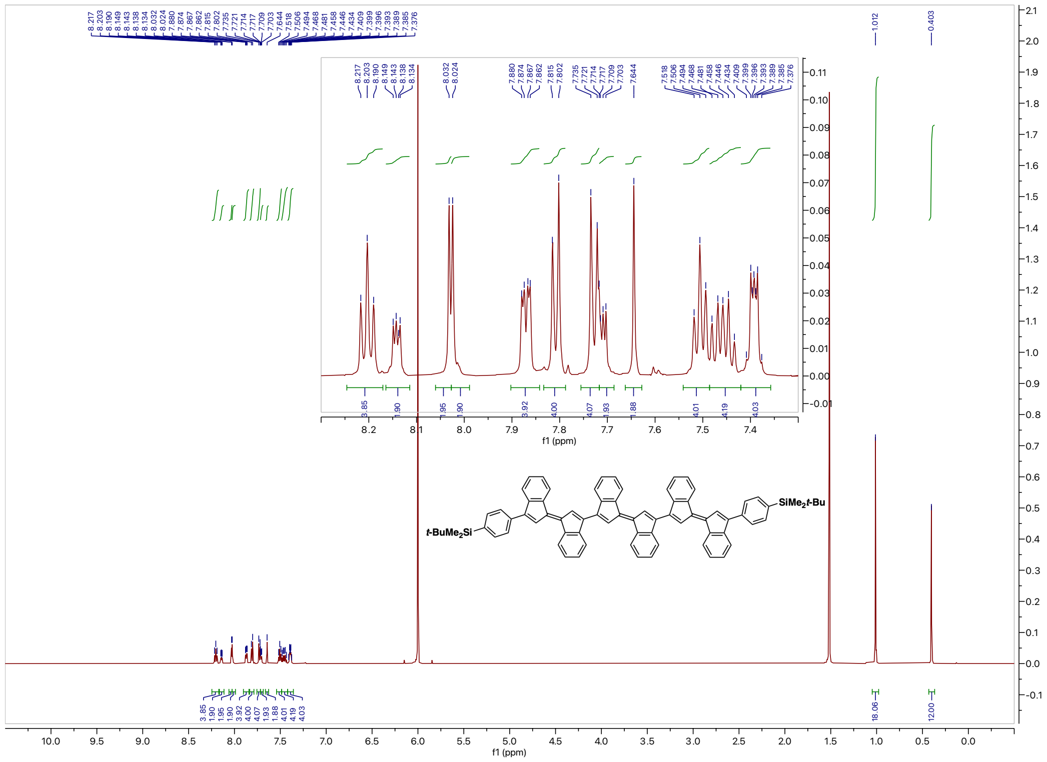

Supplementary Fig. 27: ^{1}H NMR spectrum of **4c** (600 MHz, TCE-d_2, 80 °C).

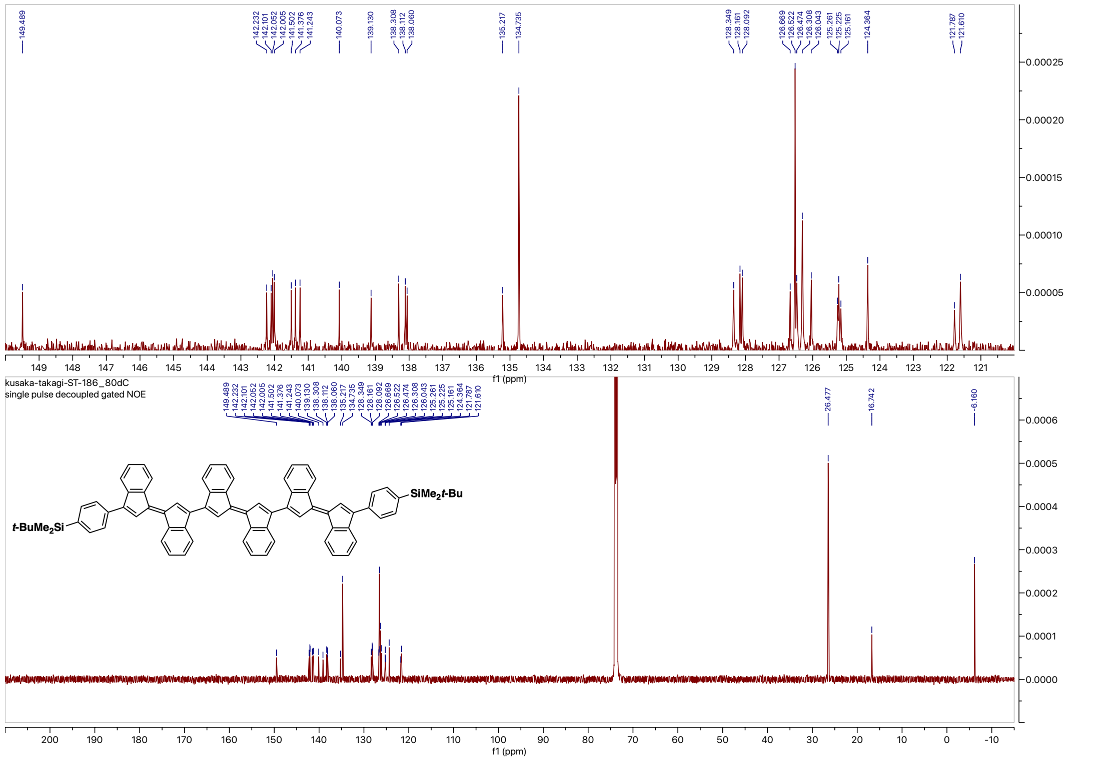

Supplementary Fig. 28: ^{13}C{^{1}H} NMR spectrum of **4c** (150 MHz, TCE-d_2, 80 °C).

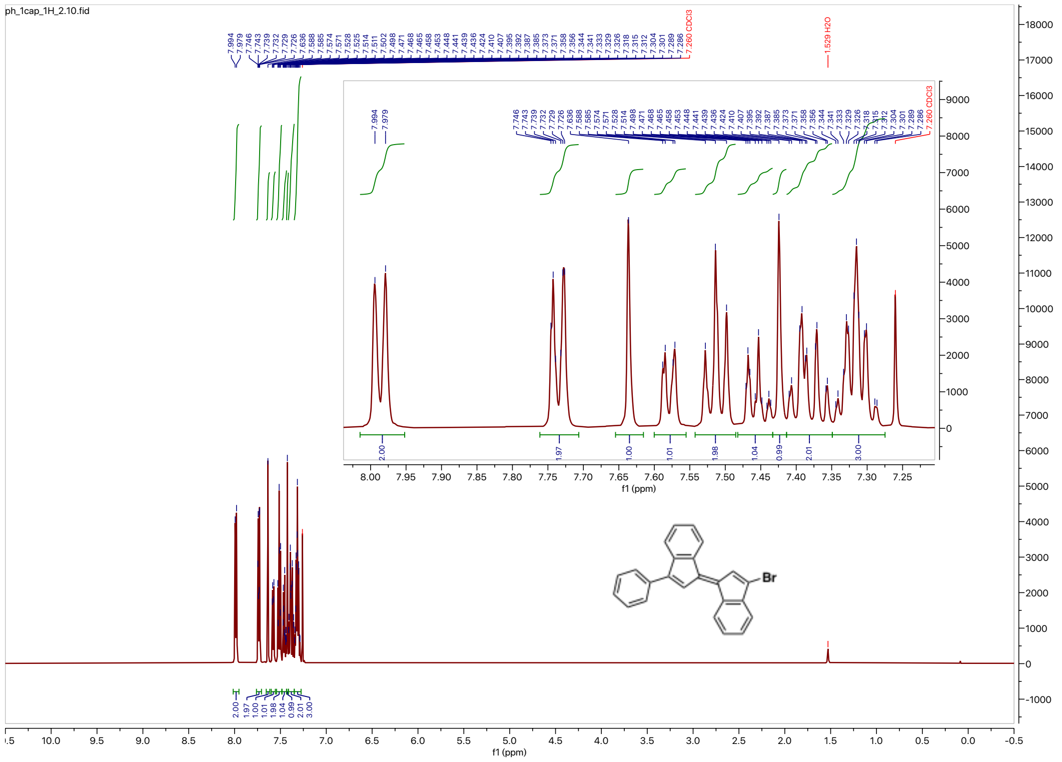

Supplementary Fig. 29: ^{1}H NMR spectrum of **6a** (500 MHz, $CDCl_3$).

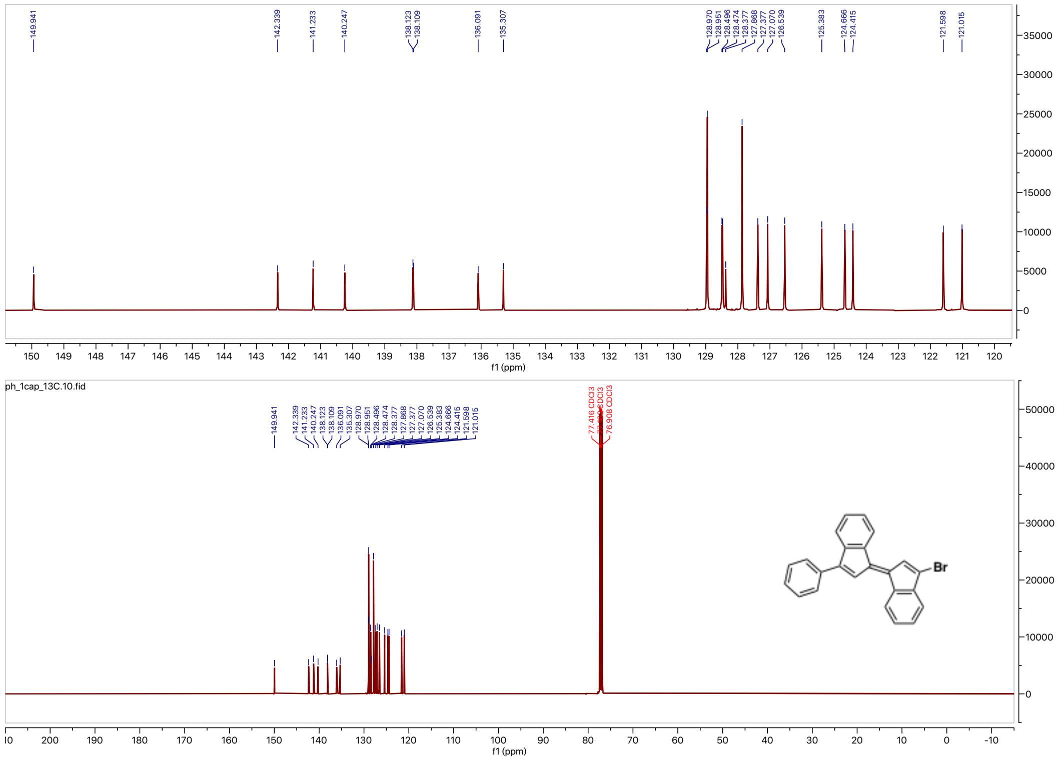

Supplementary Fig. 30: $^{13}C\{^{1}H\}$ NMR spectrum of **6a** (125 MHz, $CDCl_3$).

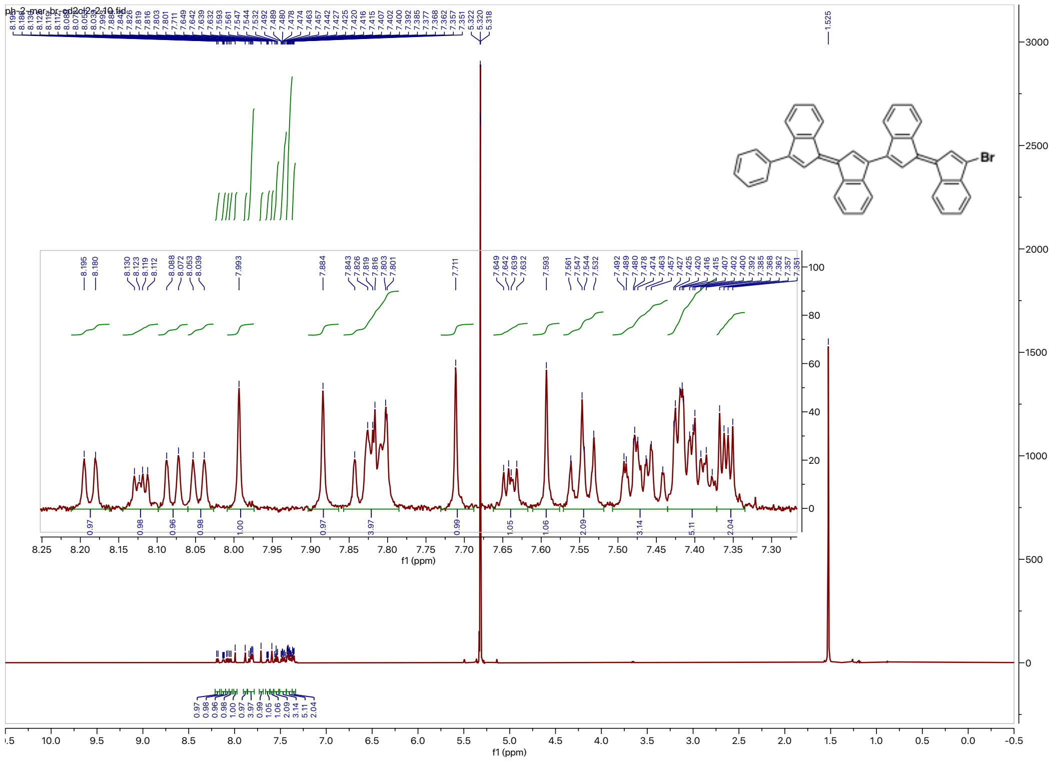

Supplementary Fig. 31: ^{1}H NMR spectrum of **6b** (500 MHz, CD_2Cl_2).

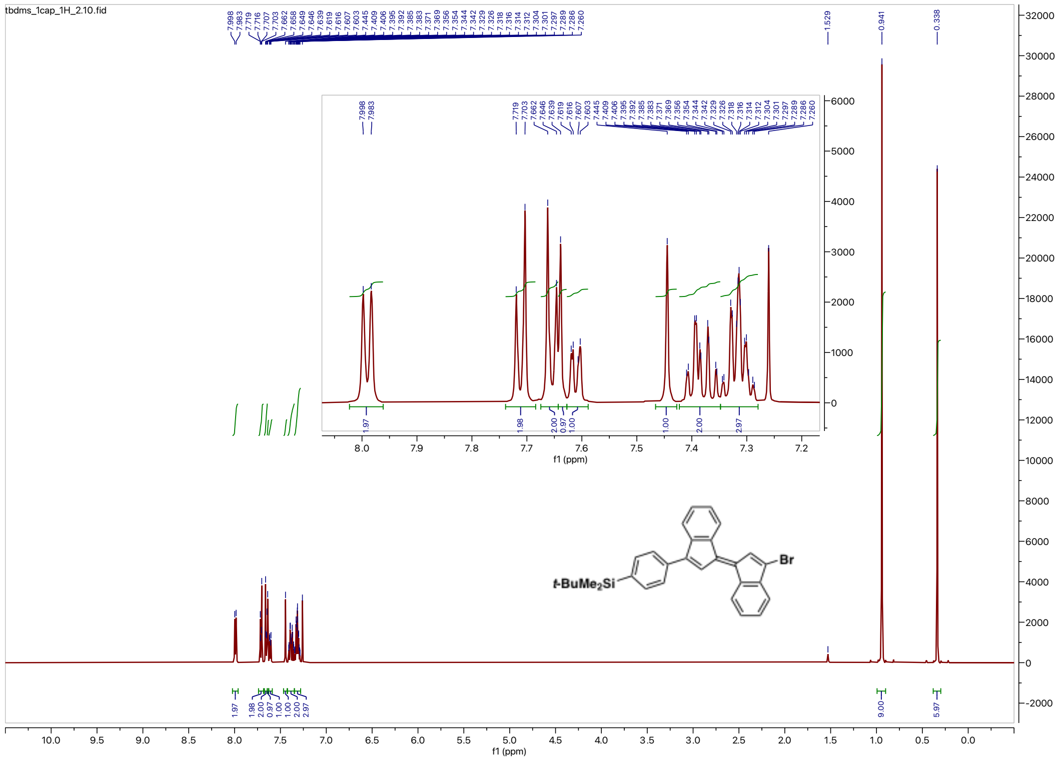

Supplementary Fig. 32: ^{1}H NMR spectrum of **7a** (500 MHz, $CDCl_3$).

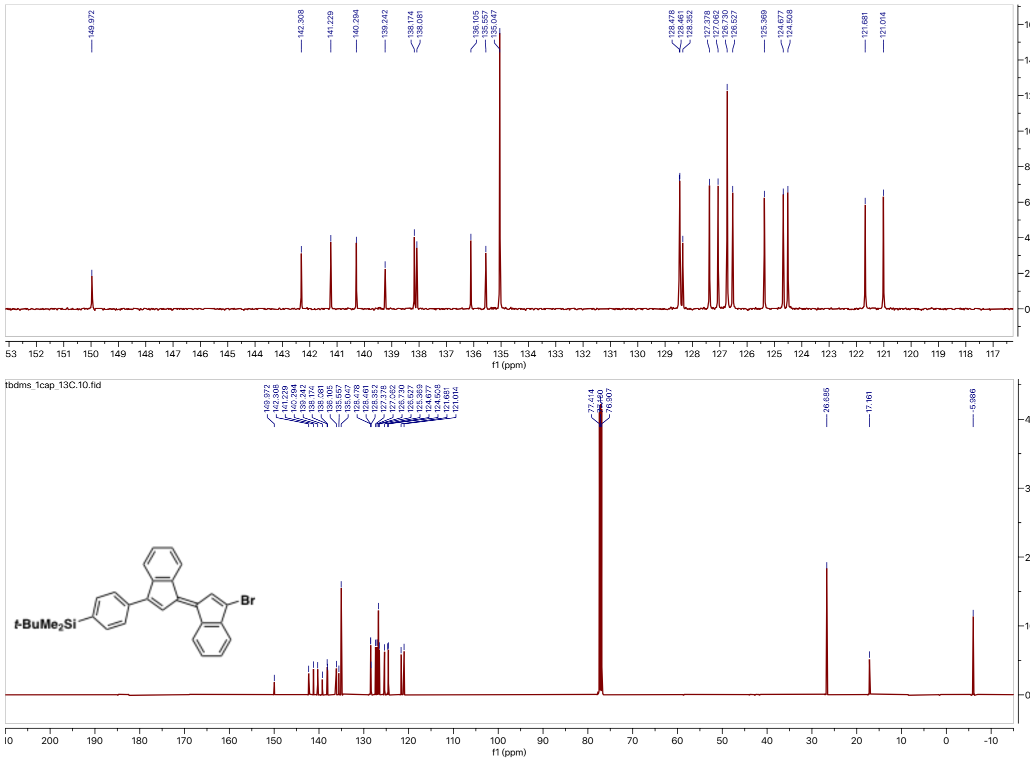

Supplementary Fig. 33: $^{13}C\{^1H\}$ NMR spectrum of **7a** (125 MHz, $CDCl_3$).

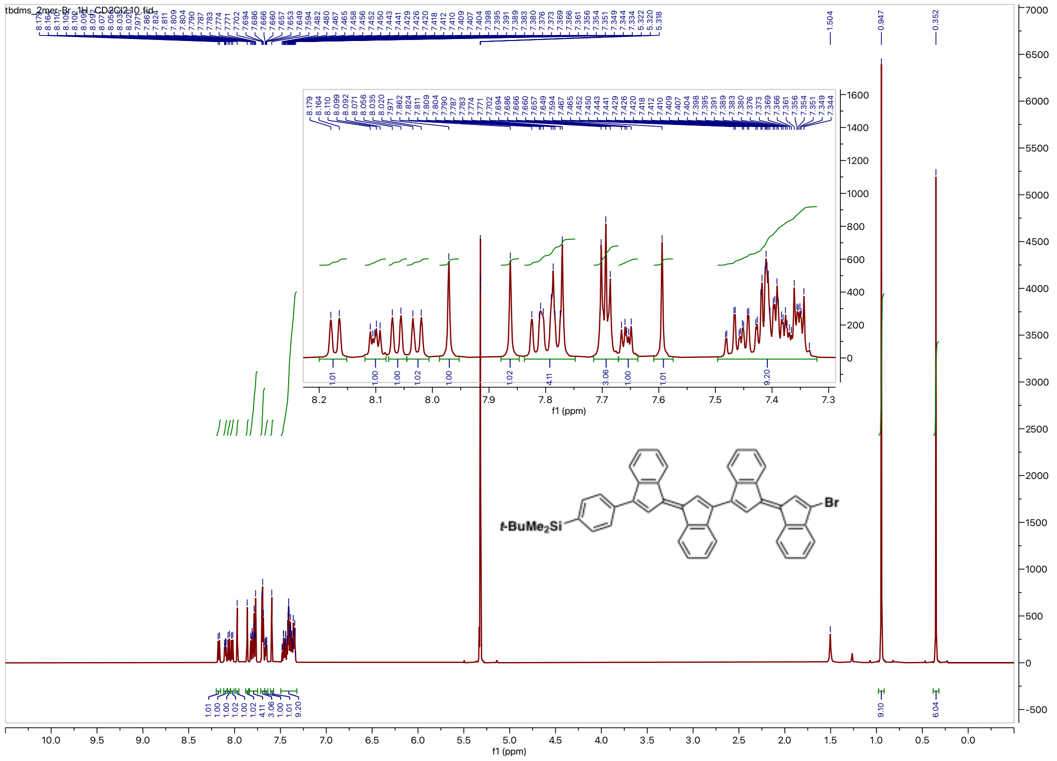

Supplementary Fig. 34: ^1H NMR spectrum of **7b** (500 MHz, CD_2Cl_2).

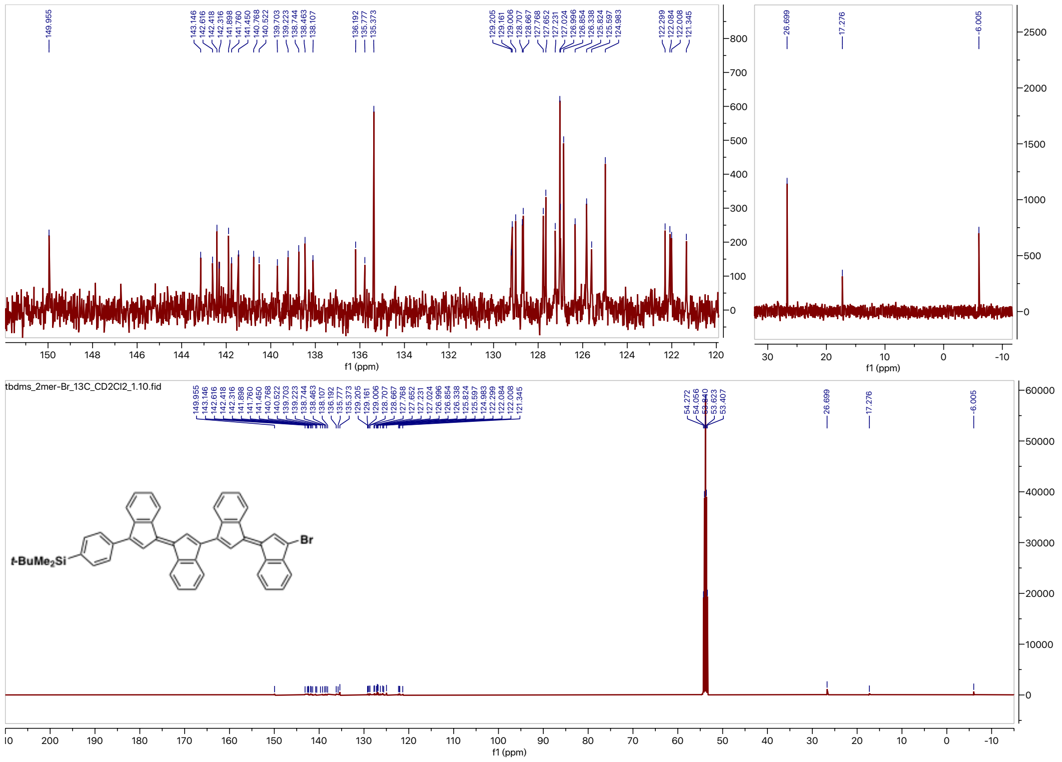

Supplementary Fig. 35: $^{13}C\{^{1}H\}$ NMR spectrum of **7b** (125 MHz, CD_2Cl_2).

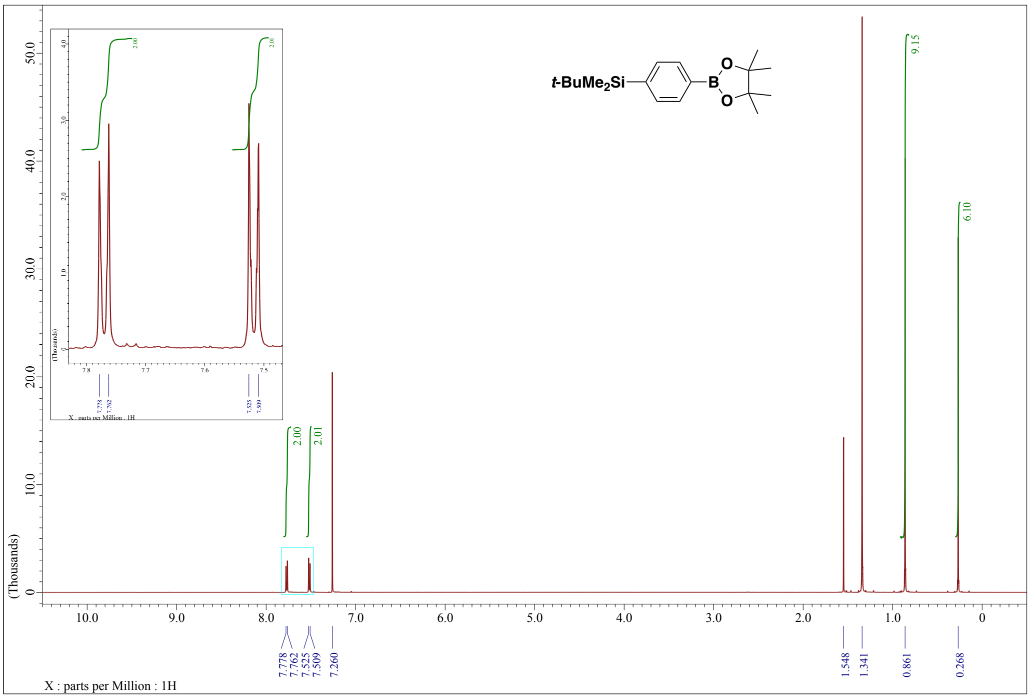

Supplementary Fig. 36: ^{1}H NMR spectrum of **1-(*tert*-butyldimethylsilyl)-4-(4,4,5,5-tetramethyl-1,3,2-dioxaborolan-2-yl)benzene** (500 MHz, $CDCl_3$).

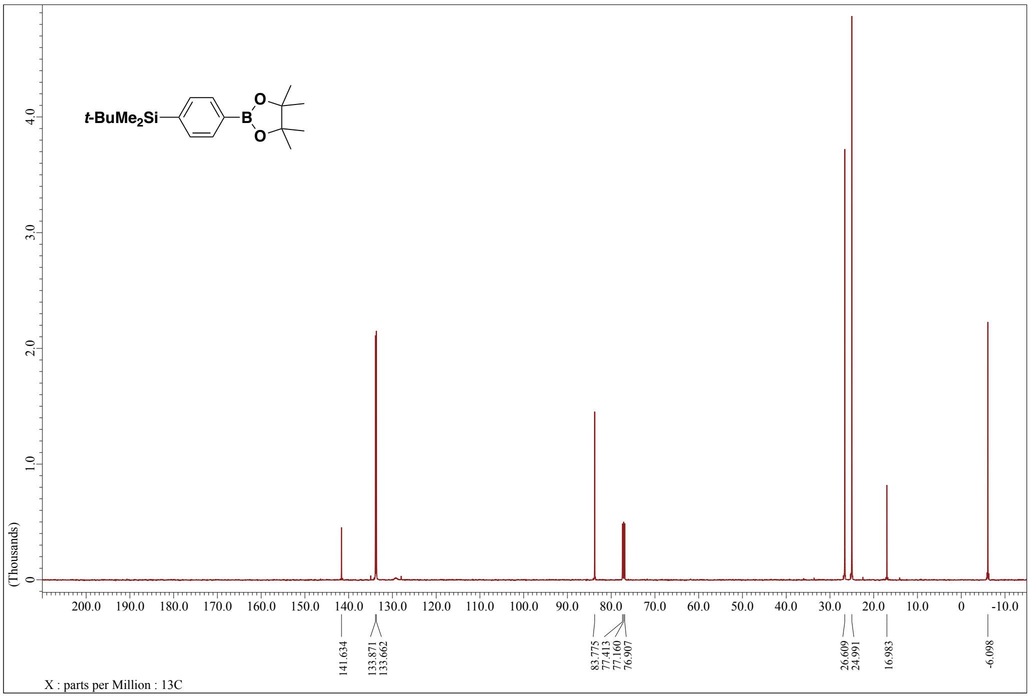

Supplementary Fig. 37: $^{13}C\{^{1}H\}$ NMR spectrum of **1-(*tert*-butyldimethylsilyl)-4-(4,4,5,5-tetramethyl-1,3,2-dioxaborolan-2-yl)benzene** (500 MHz, $CDCl_3$).

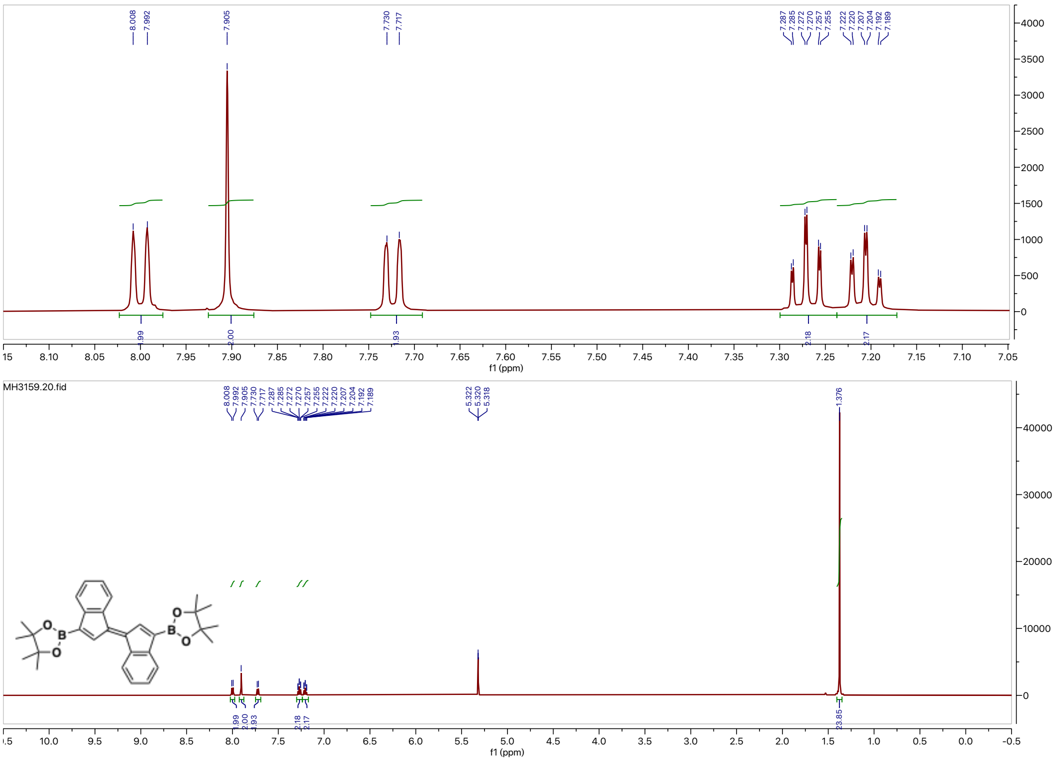

Supplementary Fig. 38: ^{1}H NMR spectrum of **8** (500 MHz, CD_2Cl_2).

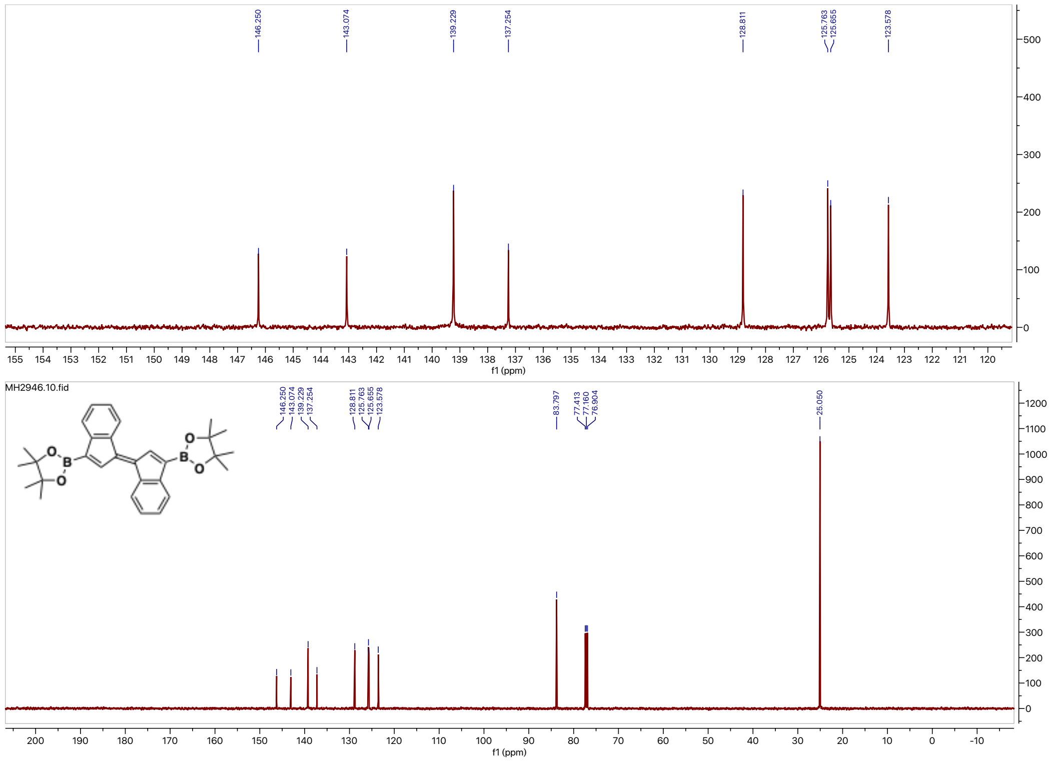

Supplementary Fig. 39: $^{13}C\{^{1}H\}$ NMR spectrum of **8** (125 MHz, $CDCl_3$).

9.3 HRMS Spectra of Newly Synthesized Compounds

Mass Spectrum SmartFormula Report

Analysis Info

Analysis Name	D:\Data\FUJIHASHI\2022\2022_01\220113\1-13-2022_Ph-monomer_68_513.d	Acquisition Date	1/13/2022 3:56:44 PM
Method	211011_APCI_pos_wide_Flow_3min.m	Operator	Demo User
Sample Name	Ph-monomer	Instrument	timsTOF 1844426.10448
Comment	MeOH > MeOH		

Acquisition Parameter

Source Type	APCI	Ion Polarity	Positive	Set Nebulizer	2.5 Bar
Focus	Not active	Set Capillary	2500 V	Set Dry Heater	200 °C
Scan Begin	50 m/z	Set End Plate Offset	-500 V	Set Dry Gas	4.0 l/min
Scan End	3000 m/z	Set Collision Cell RF	600.0 Vpp	Set Divert Valve	Waste

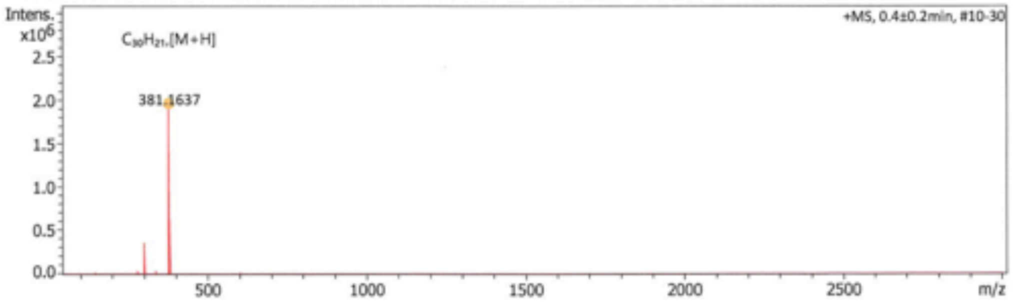

	Meas. m/z	#	Ion Formula	m/z	err [ppm]	mSigma	# mSigma	Score	rdb	e⁻ Conf	N-Rule
[M+H]+ →	381.1637	1	C30H21	381.1638	0.2	4.6	1	100.00	21.0	even	ok

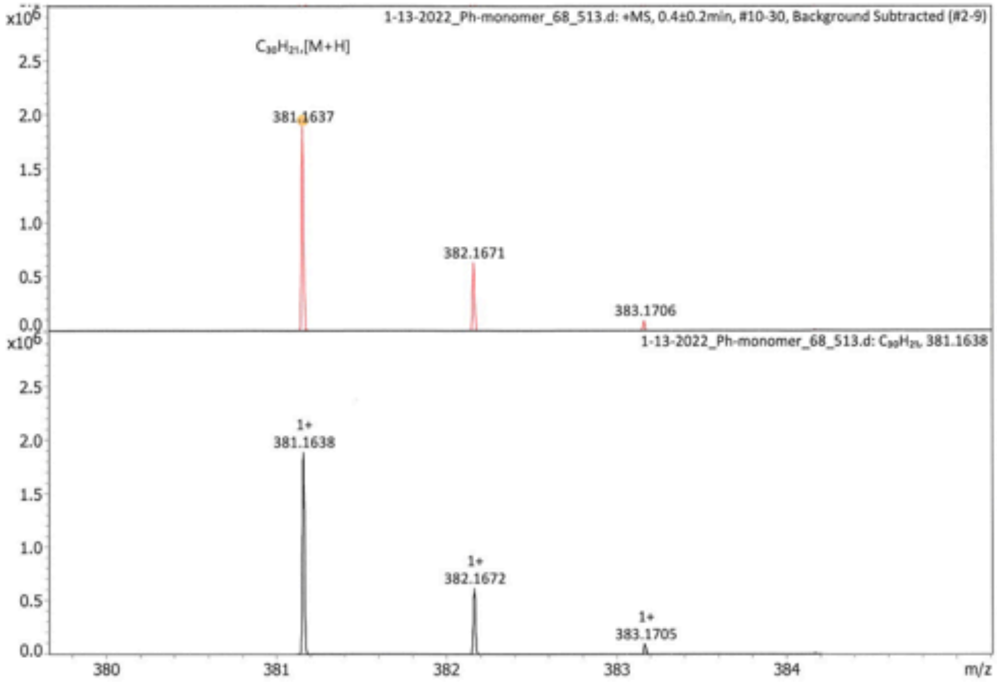

Bruker Compass DataAnalysis 5.3 printed: 1/13/2022 4:41:58 PM by: demo Page 1 of 1

Supplementary Fig. 40: High-resolution mass spectrum of **3a** (APCI(+)). Overall view of the experimental spectrum (top), enlarged view of the signals corresponding to $[M\text{+H}]^+$ (middle), and the simulated spectrum (bottom).

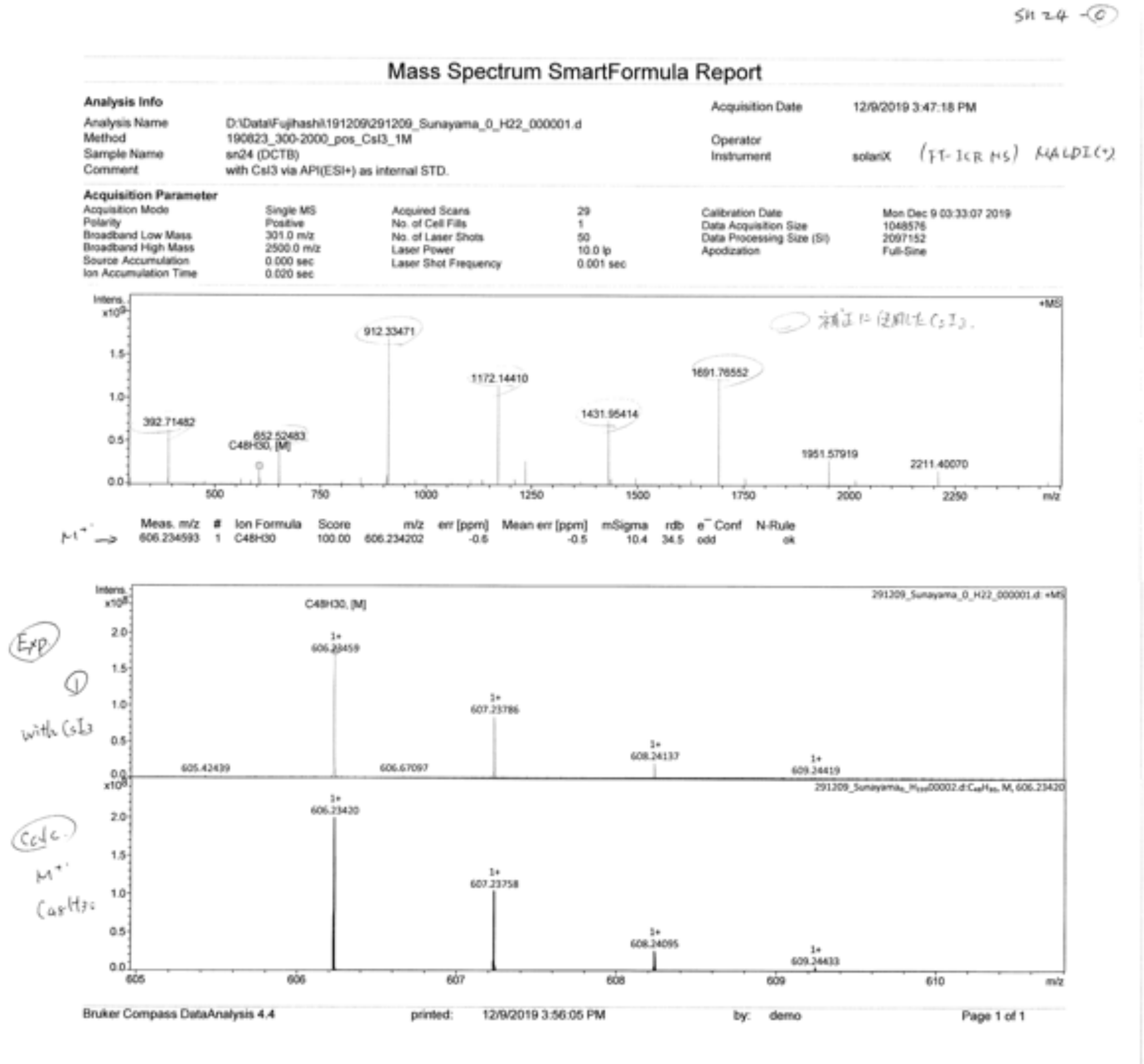

Supplementary Fig. 41: High-resolution mass spectrum of **3b** (MALDI(+)). Overall view of the experimental spectrum (top), enlarged view of the signals corresponding to $[M]^+$ (middle), and the simulated spectrum (bottom).

Mass Spectrum SmartFormula Report

Analysis Info

Analysis Name	D:\Data\Fujihashi\191220\191220_sunayama_0_E1_000001.d	Acquisition Date	12/20/2019 2:51:47 PM
Method	191108_130-2000_pos_CsI3_2M	Operator	
Sample Name	sn26-1 (DCTB)	Instrument	solariX
Comment			

Acquisition Parameter

Acquisition Mode	Single MS	Acquired Scans	50	Calibration Date	Fri Dec 20 02:48:39 2019
Polarity	Positive	No. of Cell Fills	1	Data Acquisition Size	2097152
Broadband Low Mass	100.3 m/z	No. of Laser Shots	100	Data Processing Size	4194304
Broadband High Mass	2500.0 m/z	Laser Power	10.0 lp	Apodization	Full-Sine
Source Accumulation	0.000 sec	Laser Shot Frequency	0.001 sec		
Ion Accumulation Time	0.050 sec				

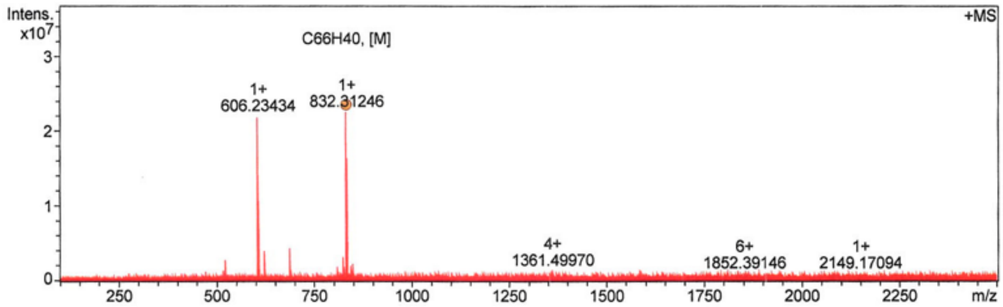

Meas. m/z	#	Ion Formula	Score	m/z	err [ppm]	Mean err [ppm]	mSigma	rdb	e⁻ Conf	N-Rule
832.312459	1	C66H40	100.00	832.312453	-0.0	-0.0	9.1	47.5	odd	ok

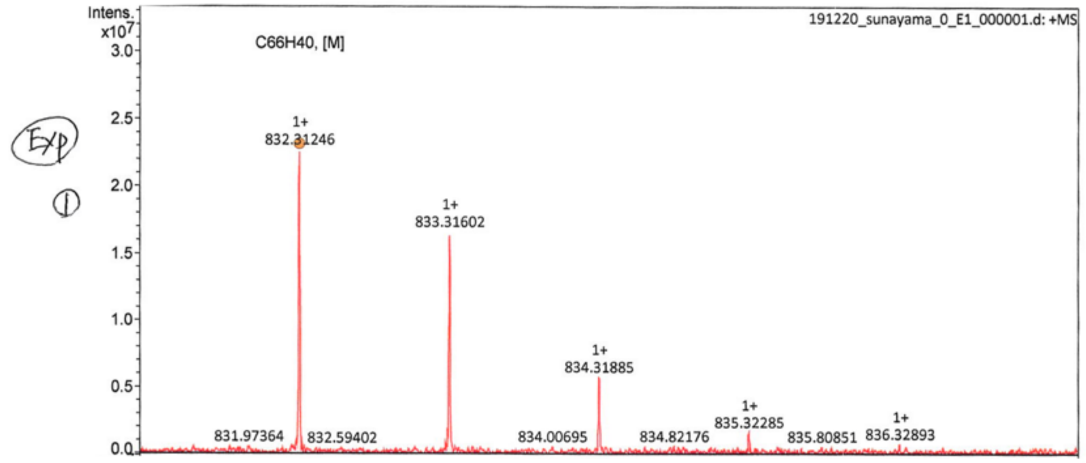

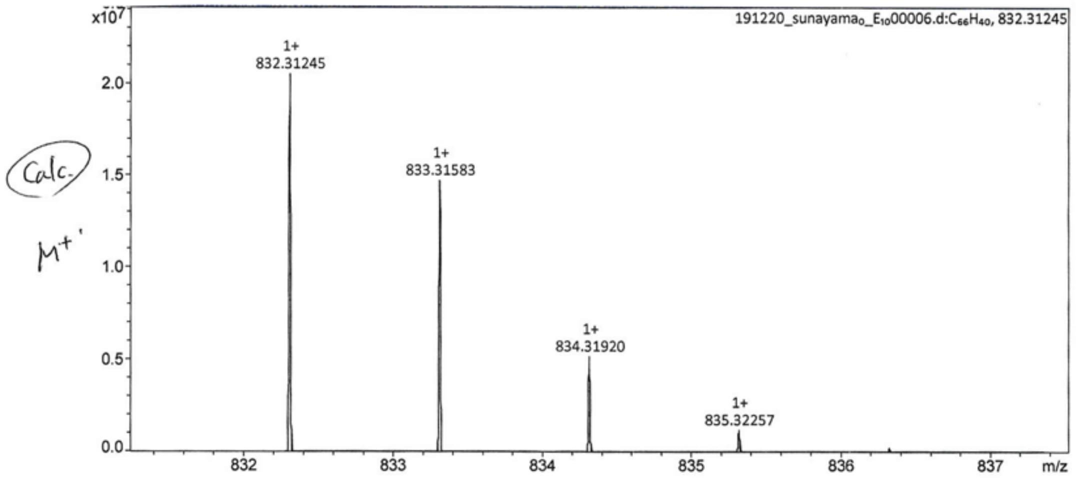

Supplementary Fig. 42: High-resolution mass spectrum of **3c** (MALDI(+)). Overall view of the experimental spectrum (top), enlarged view of the signals corresponding to $[M]^+$ (middle), and the simulated spectrum (bottom).

Mass Spectrum SmartFormula Report

Analysis Info

Analysis Name	D:\Data\FUJIHASHI\2022\2022_01\220113\1-13-2022_TBDMSPh-Fv_67_512.d	Acquisition Date	1/13/2022 3:53:20 PM
Method	211011_APCI_pos_wide_Flow_3min.m	Operator	Demo User
Sample Name	TBDMSPh-Fv	Instrument	timsTOF 1844426.10448
Comment	MeOH > MeOH		

Acquisition Parameter

Source Type	APCI	Ion Polarity	Positive	Set Nebulizer	2.5 Bar
Focus	Not active	Set Capillary	2500 V	Set Dry Heater	200 °C
Scan Begin	50 m/z	Set End Plate Offset	-500 V	Set Dry Gas	4.0 l/min
Scan End	3000 m/z	Set Collision Cell RF	600.0 Vpp	Set Divert Valve	Waste

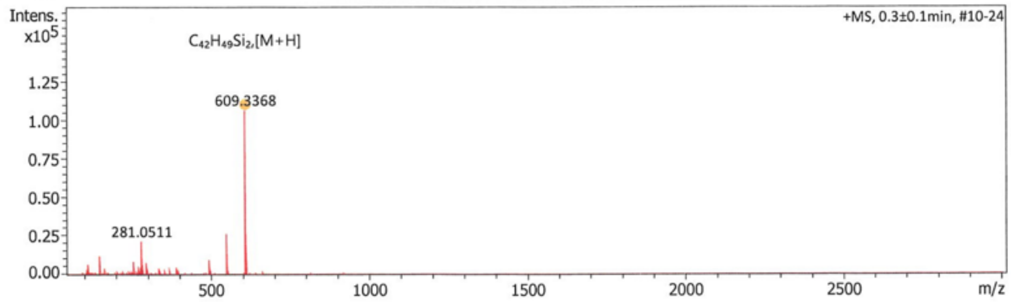

	Meas. m/z	#	Ion Formula	m/z	err [ppm]	mSigma	# mSigma	Score	rdb	e⁻ Conf	N-Rule
[M+H]⁺ →	609.3368	1	C42H49Si2	609.3367	-0.1	20.3	1	100.00	21.0	even	ok

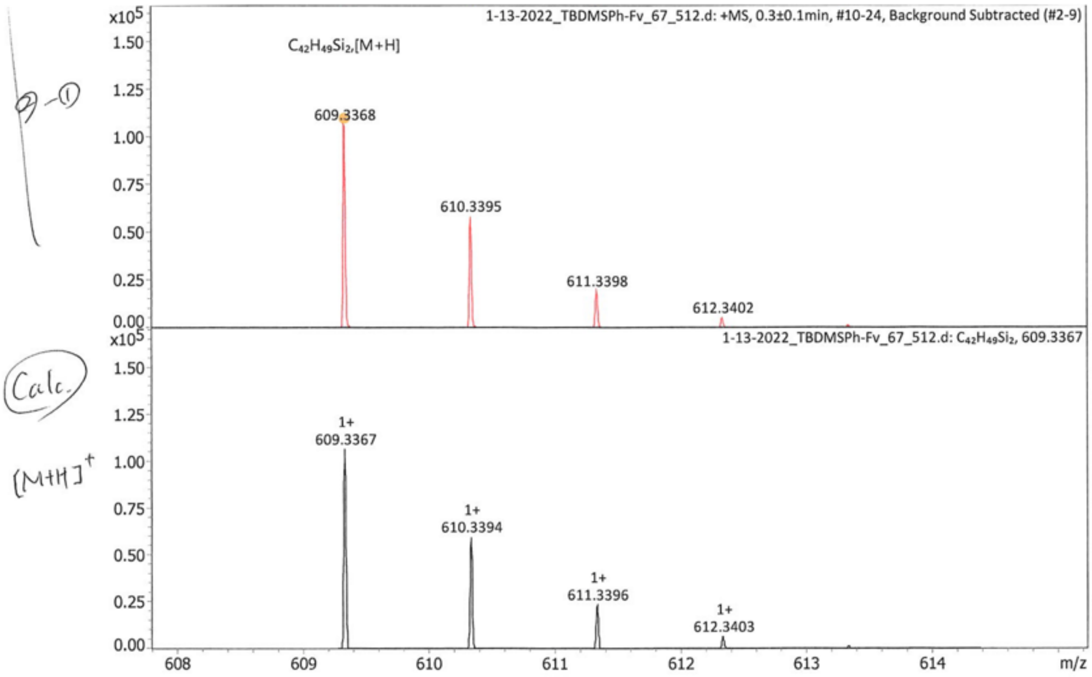

Bruker Compass DataAnalysis 5.3 printed: 1/13/2022 4:37:21 PM by: demo Page 1 of 1

Supplementary Fig. 43: High-resolution mass spectrum of **4a** (APCI(+)). Overall view of the experimental spectrum (top), enlarged view of the signals corresponding to [M+H]$^+$ (middle), and the simulated spectrum (bottom).

Mass Spectrum SmartFormula Report

Analysis Info

Analysis	D:\Data\FUJIHASHI\2022\2022_07\20220729\7-29-2022_TBDMS-Fv-Dimer_27_1642.d	Acquisition Date	7/29/2022 3:24:59 PM
Method	211011_APCI_pos_wide_Flow_3min.m	Operator	Demo User
Sample	TBDMS-Fv-Dimer	Instrument	timsTOF 1844426.10448
Comment	MeOH (flow: MeOH)		

Acquisition Parameter

Source Type	APCI	Ion Polarity	Positive	Set Nebulizer	2.5 Bar
Scan Begin	50 m/z	Set Capillary	2500 V	Set Dry Heater	200 °C
Scan End	3000 m/z	Set Multipole RF	300.0 Vpp	Set Dry Gas	4.0 l/min
		Set Collision Cell RF	600.0 Vpp	ICC active	Off
		IMS Active	Off	ICC Target	2000000 cts.
		IMS Collision Cell In	300.0 V		

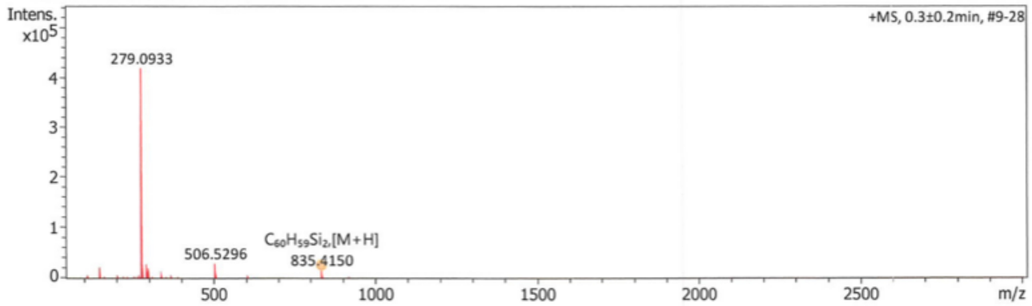

Meas. m/z	#	Ion Formula	m/z	err [ppm]	mSigma	# mSigma	Score	rdb	e⁻ Conf	N-Rule
835.4150	1	C60H59Si2	835.4150	-0.0	17.9	1	100.00	34.0	even	ok

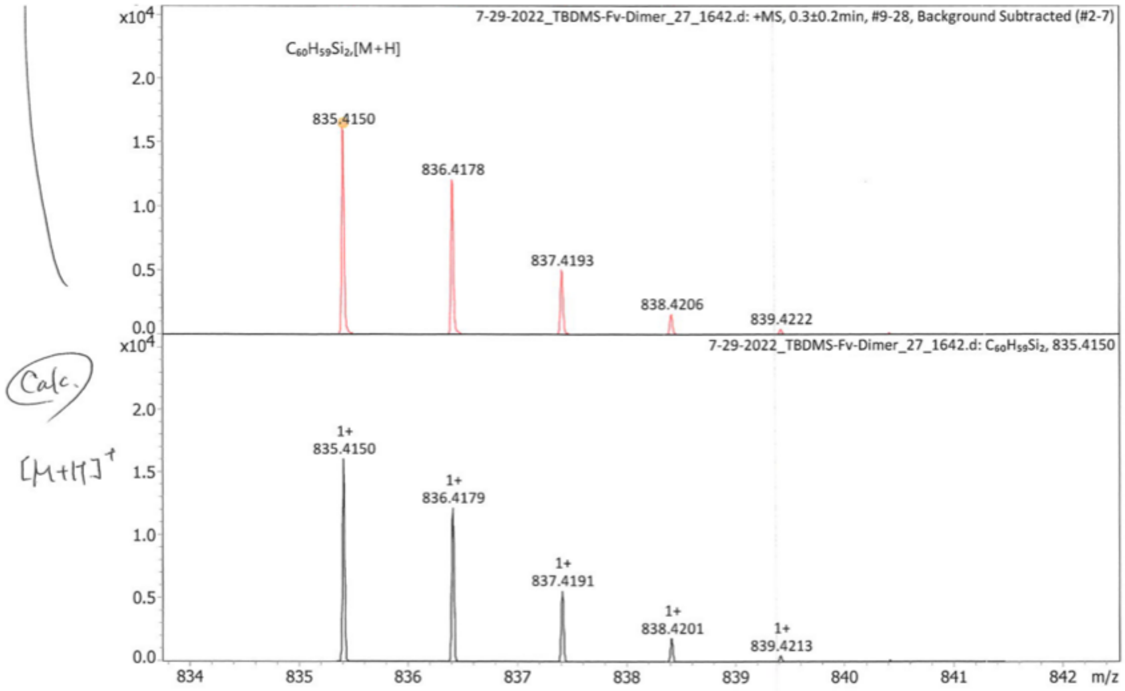

Bruker Compass DataAnalysis 5.3 printed: 7/29/2022 3:33:59 PM by: demo Page 1 of 1

Supplementary Fig. 44: High-resolution mass spectrum of **4b** (APCI(+)). Overall view of the experimental spectrum (top), enlarged view of the signals corresponding to $[M+H]^+$ (middle), and the simulated spectrum (bottom).

Mass Spectrum SmartFormula Report

Analysis Info

Analysis Name	D:\Data\Fujihashi\2021\2021_03\210305\210305_sn126-2_0_C2_000002.d	Acquisition Date	3/5/2021 12:00:57 PM
Method	201125_300-3000_pos_CsI3_2M	Operator	
Sample Name	sn126-2	Instrument	solariX
Comment	Ion Source : MALDI<br>Matrix : DCTB<br>with CsI3 via API (ESI+) as an internal calibrant.		

Acquisition Parameter

Acquisition Mode	Single MS	Acquired Scans	9	Calibration Date	Fri Mar 5 11:54:40 2021
Polarity	Positive	No. of Cell Fills	1	Data Acquisition Size	2097152
Broadband Low Mass	301.0 m/z	No. of Laser Shots	50	Data Processing Size (SI)	4194304
Broadband High Mass	3000.0 m/z	Laser Power	8.0 lp	Apodization	Full-Sine
Source Accumulation	0.000 sec	Laser Shot Frequency	0.001 sec		
Ion Accumulation Time	0.080 sec				

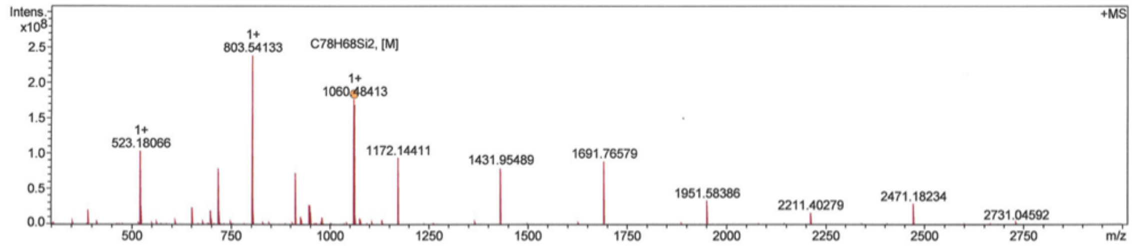

Meas. m/z	#	Ion Formula	Score	m/z	err [ppm]	Mean err [ppm]	mSigma	rdb	e⁻ Conf	N-Rule
1060.484131	1	C78H68Si2	100.00	1060.485407	1.2	0.8	48.9	47.5	odd	ok

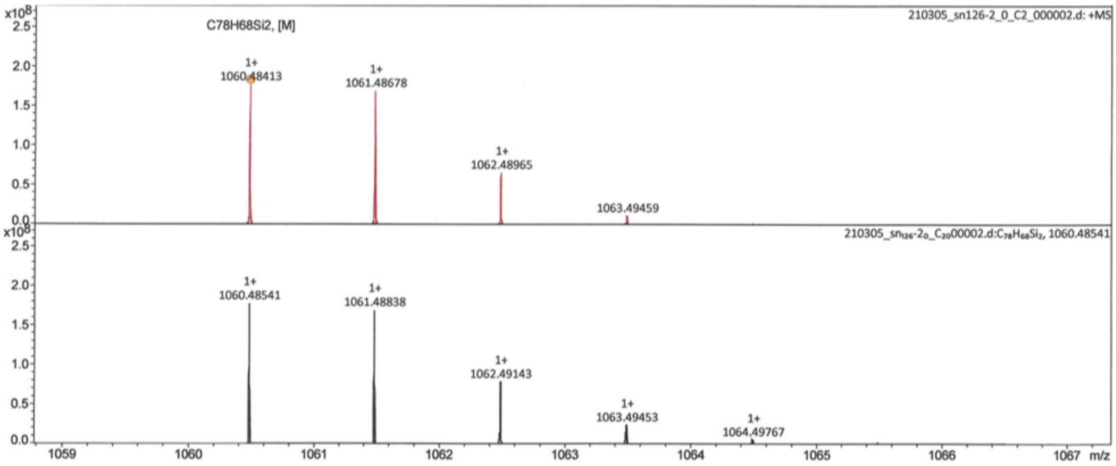

Bruker Compass DataAnalysis 4.4 printed: 3/5/2021 12:05:40 PM by: demo Page 1 of 1

Supplementary Fig. 45: High-resolution mass spectrum of **4c** (MALDI(+)). Overall view of the experimental spectrum (top), enlarged view of the signals corresponding to $[M]^+$ (middle), and the simulated spectrum (bottom).

Mass Spectrum SmartFormula Report

Analysis Info

Analysis Name	D:\Data\FUJIHASHI\2022\2022_01\220118\1-18-2022_Sn2_76_537.d	Acquisition Date	1/18/2022 12:10:02 PM
Method	211011_APCI_pos_wide_Flow_3min.m	Operator	Demo User
Sample Name	Sn2	Instrument	timsTOF 1844426.10448
Comment	MeOH > MeOH		

Acquisition Parameter

Source Type	APCI	Ion Polarity	Positive	Set Nebulizer	2.5 Bar
Focus	Not active	Set Capillary	2500 V	Set Dry Heater	200 °C
Scan Begin	50 m/z	Set End Plate Offset	-500 V	Set Dry Gas	4.0 l/min
Scan End	3000 m/z	Set Collision Cell RF	600.0 Vpp	Set Divert Valve	Waste

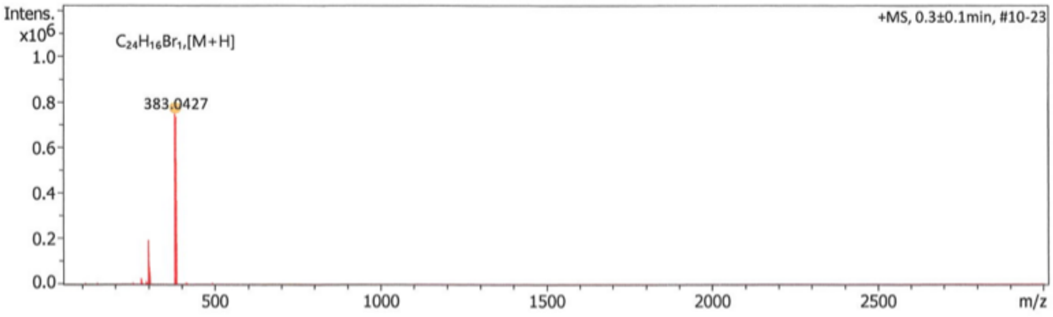

Meas. m/z	#	Ion Formula	m/z	err [ppm]	mSigma	# mSigma	Score	rdb	e⁻ Conf	N-Rule
383.0427	1	C24H16Br	383.0430	0.8	8.4	1	100.00	19.0	even	ok

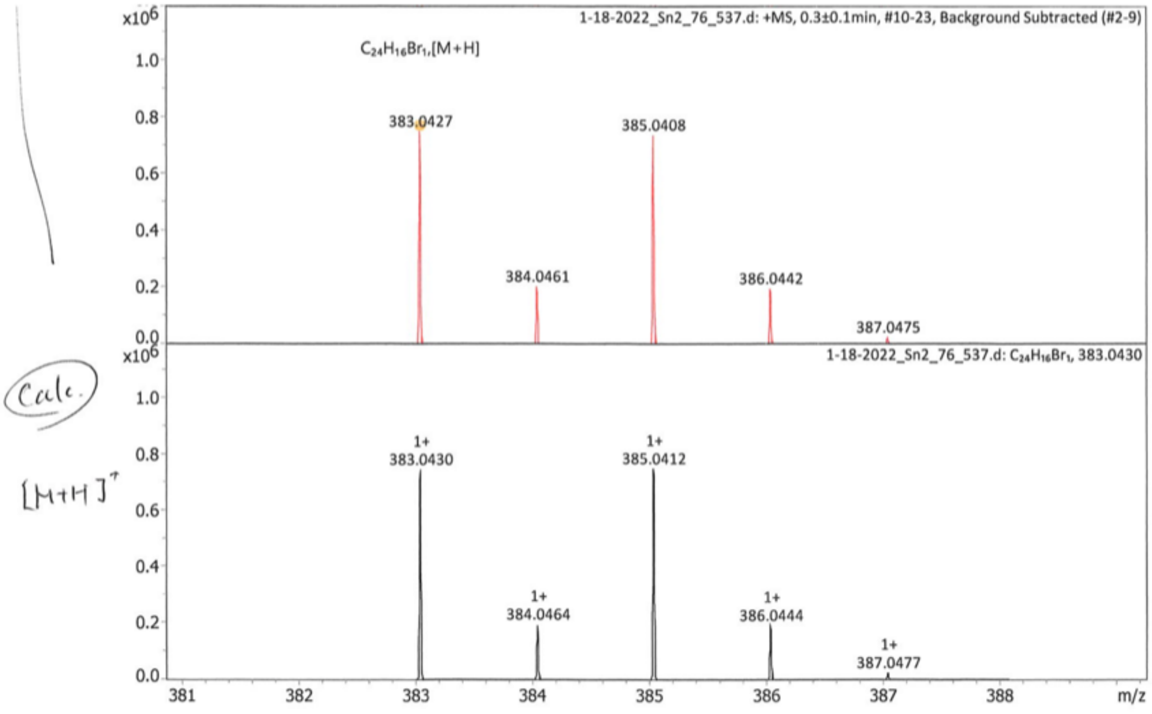

Bruker Compass DataAnalysis 5.3 printed: 1/19/2022 11:20:08 AM by: demo Page 1 of 1

Supplementary Fig. 46: High-resolution mass spectrum of **6a** (APCI(+)). Overall view of the experimental spectrum (top), enlarged view of the signals corresponding to [M+H]$^+$ (middle), and the simulated spectrum (bottom).

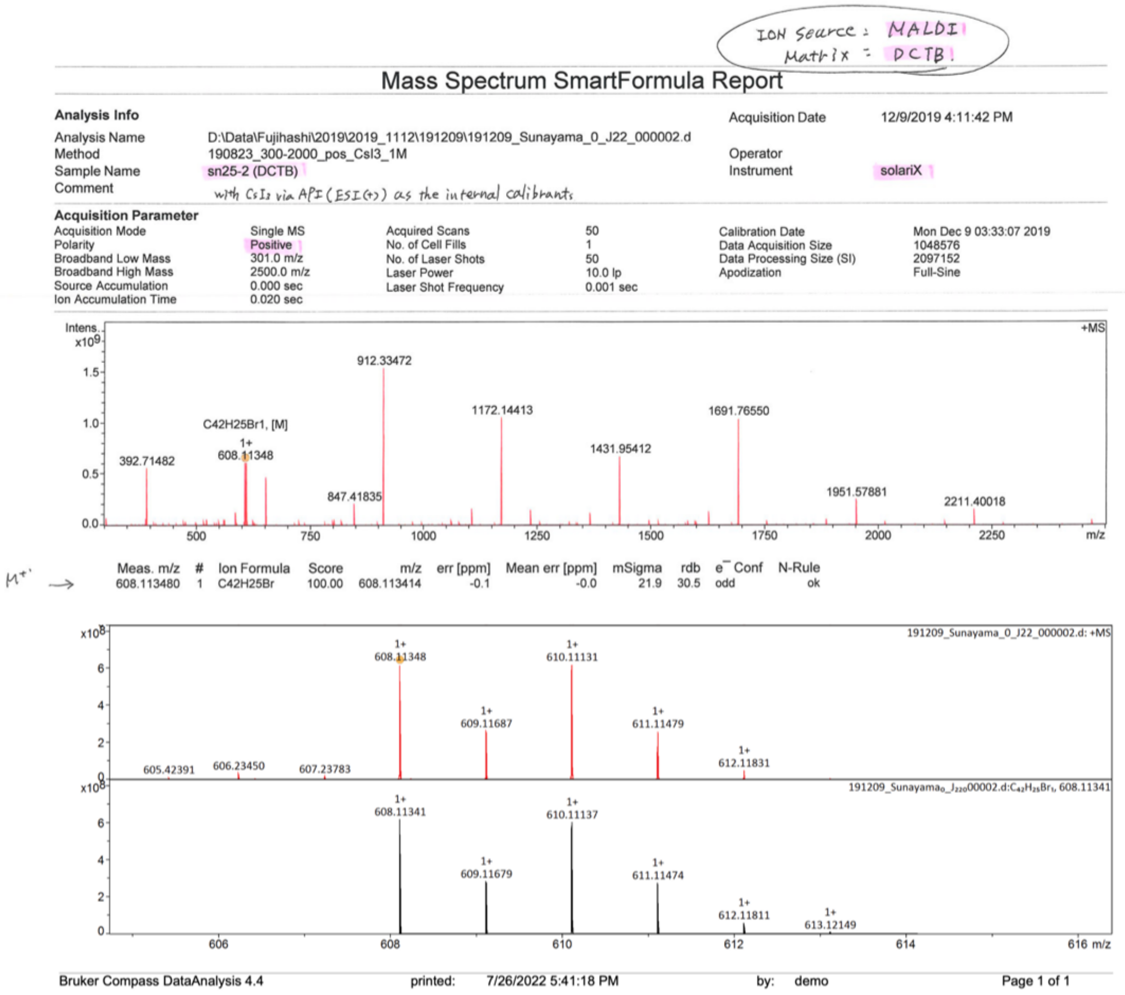

Supplementary Fig. 47: High-resolution mass spectrum of **6b** (MALDI(+)). Overall view of the experimental spectrum (top), enlarged view of the signals corresponding to $[M]^+$ (middle), and the simulated spectrum (bottom).

Mass Spectrum SmartFormula Report

Analysis Info

Analysis Name	D:\Data\FUJIHASHI\2022\2022_01\220118\1-18-2022_Sn3_77_538.d	Acquisition Date	1/18/2022 12:14:54 PM
Method	211011_APCI_pos_wide_Flow_3min.m	Operator	Demo User
Sample Name	Sn3	Instrument	timsTOF 1844426.10448
Comment	MeOH > MeOH		

Acquisition Parameter

Source Type	APCI	Ion Polarity	Positive	Set Nebulizer	2.5 Bar
Focus	Not active	Set Capillary	2500 V	Set Dry Heater	200 °C
Scan Begin	50 m/z	Set End Plate Offset	-500 V	Set Dry Gas	4.0 l/min
Scan End	3000 m/z	Set Collision Cell RF	600.0 Vpp	Set Divert Valve	Waste

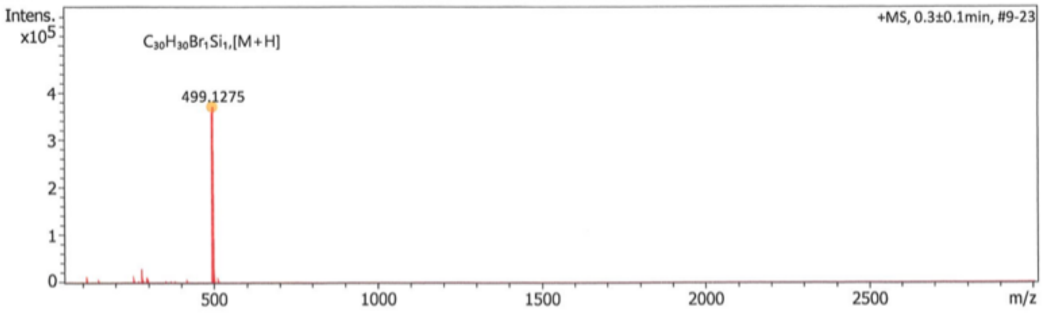

Meas. m/z	#	Ion Formula	m/z	err [ppm]	mSigma	# mSigma	Score	rdb	e⁻ Conf	N-Rule
497.1292	1	C30H30BrSi	497.1295	0.5	15.8	1	100.00	19.0	even	ok

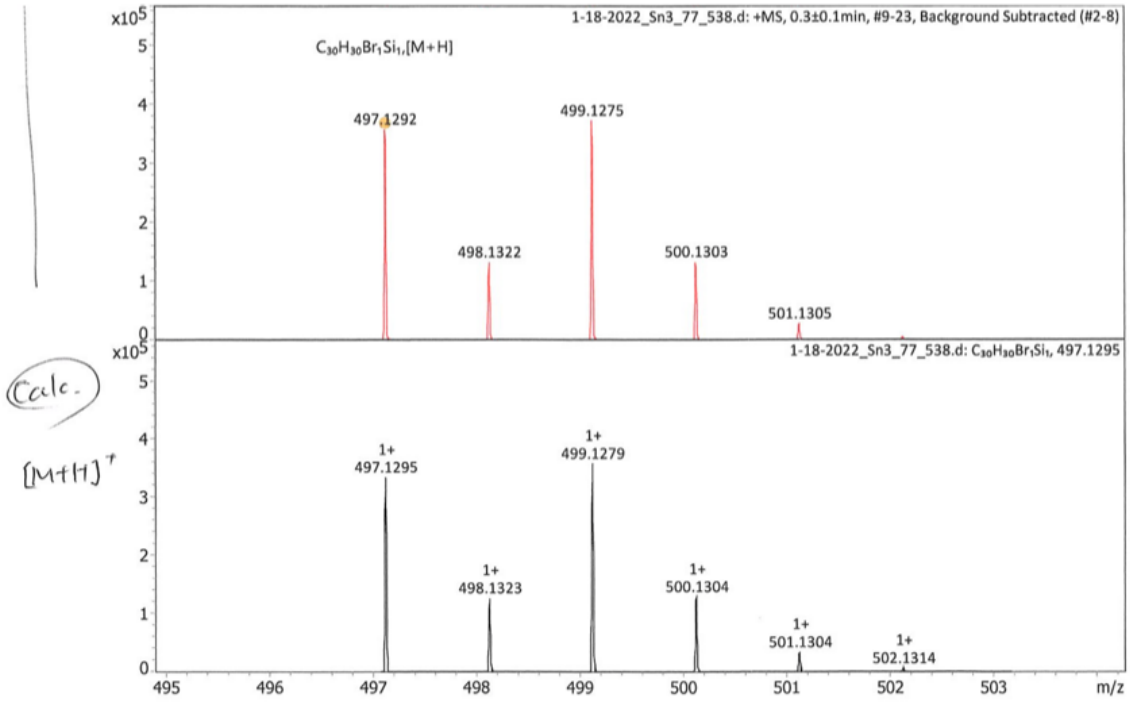

Supplementary Fig. 48: High-resolution mass spectrum of **7a** (APCI(+)). Overall view of the experimental spectrum (top), enlarged view of the signals corresponding to $[M+H]^+$ (middle), and the simulated spectrum (bottom).

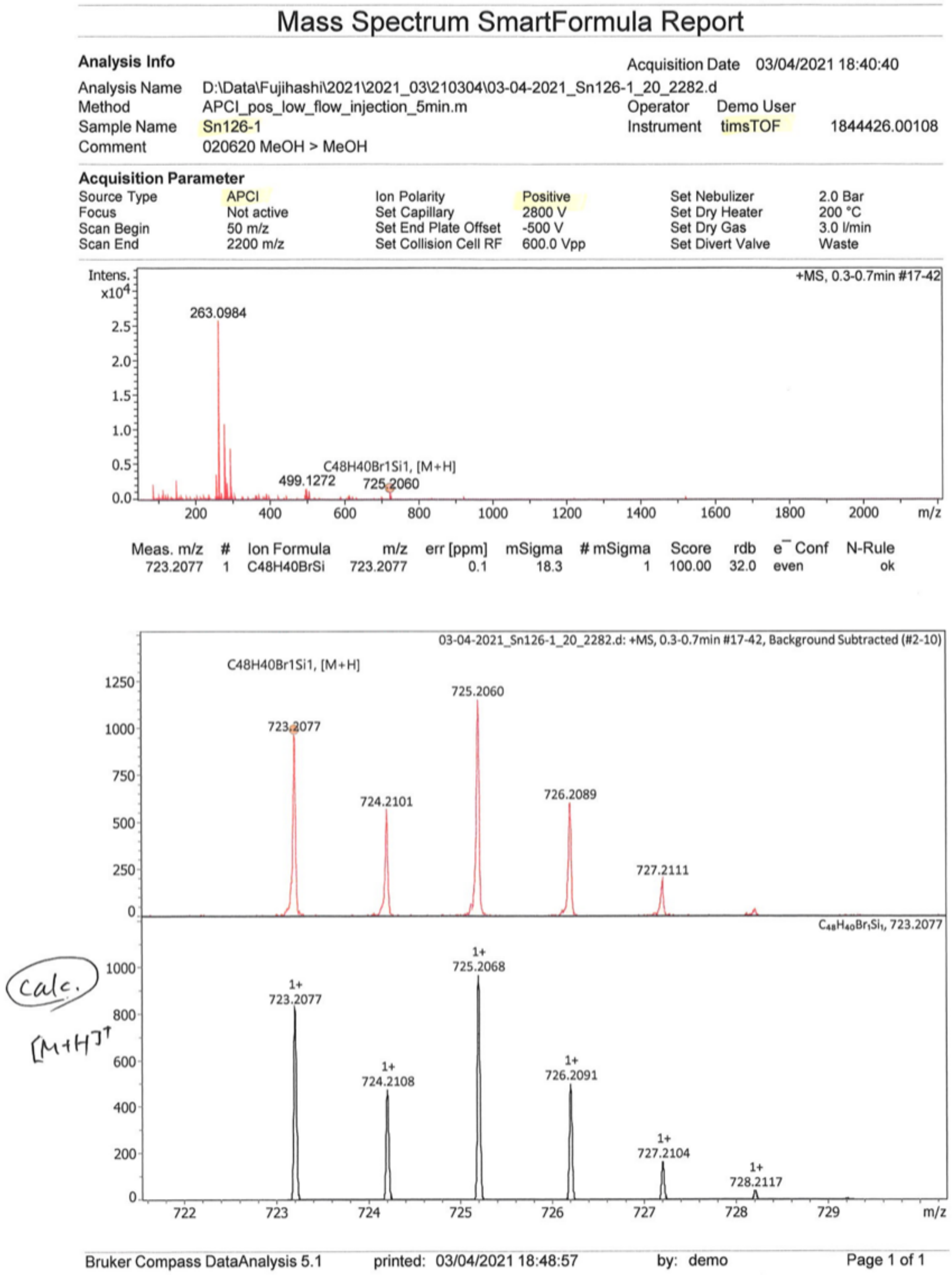

Supplementary Fig. 49: High-resolution mass spectrum of **7b** (APCI(+)). Overall view of the experimental spectrum (top), enlarged view of the signals corresponding to [*M*+H]$^{+}$ (middle), and the simulated spectrum (bottom).

Mass Spectrum SmartFormula Report

Analysis Info

Analysis Name	D:\Data\Fujihashi\2021\2021_05\210512\05-12-2021_Fv-Bpin_5_2491.d	Acquisition Date	05/12/2021 15:08:36
Method	APCI_pos_low_flow_injection_5min.m	Operator	Demo User
Sample Name	Fv-Bpin	Instrument	timsTOF 1844426.00108
Comment	MeOH > MeOH		

Acquisition Parameter

Source Type	APCI	Ion Polarity	Positive	Set Nebulizer	2.0 Bar
Focus	Not active	Set Capillary	2800 V	Set Dry Heater	200 °C
Scan Begin	50 m/z	Set End Plate Offset	-500 V	Set Dry Gas	3.0 l/min
Scan End	2200 m/z	Set Collision Cell RF	600.0 Vpp	Set Divert Valve	Waste

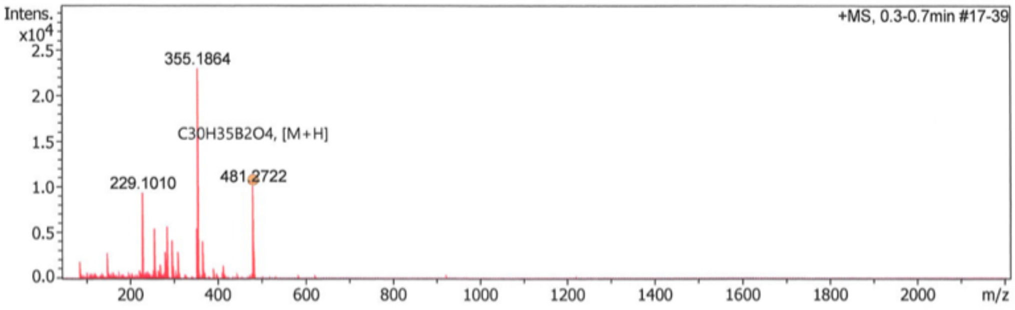

[M+H]+ →

Meas. m/z	#	Ion Formula	m/z	err [ppm]	mSigma	# mSigma	Score	rdb	e⁻ Conf	N-Rule
481.2722	1	C30H35B2O4	481.2716	0.9	9.2	1	100.00	15.0	even	ok

Calc.

[M+H]+

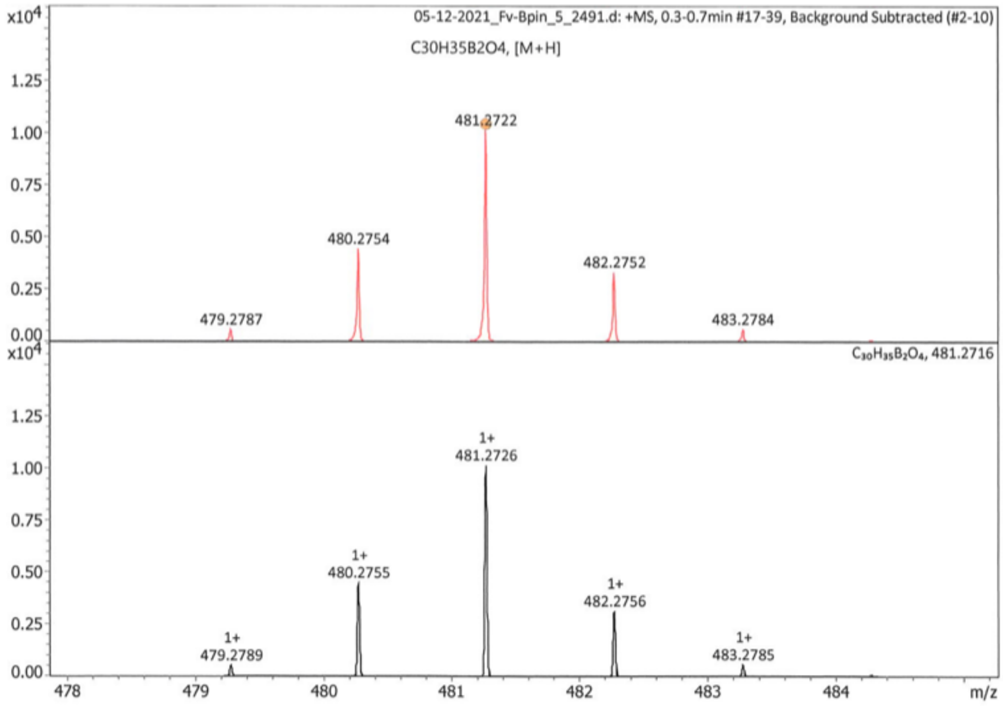

Bruker Compass DataAnalysis 5.1 printed: 05/12/2021 15:32:59 by: demo Page 1 of 1

Supplementary Fig. 50: High-resolution mass spectrum of **8** (APCI(+)). Overall view of the experimental spectrum (top), enlarged view of the signals corresponding to [M+H]$^+$ (middle), and the simulated spectrum (bottom).

10. Supplementary References

S1 Barth, W. E. & Lawton, R. G. Dibenzo[*ghi,mno*]fluoranthene. *J. Am. Chem. Soc.* **88**, 380–381 (1966).

S2 Sygula, A. & Rabideau, P. W. A practical, large scale synthesis of the corannulene system. *J. Am. Chem. Soc.* **122**, 6323–6324 (2000).

S3 Sakurai, H., Daiko, T. & Hirao, T. A Synthesis of sumanene, a fullerene fragment. *Science* **301**, 1878 (2003).

S4 Abdourazak, A. H., Sygula, A. & Rabideau, P. W. "Locking" the bowl-shaped geometry of corannulene: cyclopentacorannulene. *J. Am. Chem. Soc.* **115**, 3010–3011 (1993).

S5 Bronstein, H. E., Choi, N. & Scott, L. T. Practical Synthesis of an Open Geodesic Polyarene with a Fullerene-type 6:6-Double Bond at the Center: Diinedno[1,2,3,4-*defg*;1′,2′,3′,4′-*mnop*]chrysene. *J. Am. Chem. Soc.* **124**, 8870–8875 (2002).

S6 Rabideau, P. W. *et al.* Buckybowls: Synthesis and ab initio calculated structure of the first semibuckminsterfullerene. *J. Am. Chem. Soc.* **116**, 7891–7892 (1994).

S7 Wu, T.-C., Hsin, H.-J., Kuo, M.-Y., Li, C.-H. & Wu, Y.-T. Synthesis and structural analysis of a highly curved buckybowl containing corannulene and sumanene fragments. *J. Am. Chem. Soc.* **133**, 16319–16321 (2011).

S8 Scott, L. T., Bratcher, M. S. & Hagen, S. Synthesis and characterization of a $C_{36}H_{12}$ fullerene subunit. *J. Am. Chem. Soc.* **118**, 8743–8744 (1996).

S9 Tanaka, Y., Fukui, N. & Shinokubo, H. *as*-Indaceno[3,2,1,8,7,6-*ghijklm*]terrylene as a near-infrared absorbing C_{70}-fragment. *Nat. Commun.* **11**, 3873 (2020).

S10 Brunetti, F. G., Varotto, A., Batara, N. A. & Wudl, F. "Deconvoluted fullerene" derivatives: synthesis and characterization. *Chem. Eur. J.* **17**, 8604–8608 (2011).

S11 Ishiyama, T., Murata, M. & Miyaura, N. Palladium(0)-Catalyzed Cross-Coupling Reaction of Alkoxydiboron with Haloarenes: A Direct Procedure for Arylboronic Esters. *J. Org. Chem.* **60**, 7508–7510 (1995).

S12 Haddon, R. C. & Scott, L. T. π-Orbital conjugation and rehybridization in bridged annulenes and deformed molecules in general: π-orbital axis vector analysis. *Pure Appl. Chem.* **58**, 137–142 (1986).

S13 Haddon, R. C. GVB and POAV analysis of rehybridization and π-orbital misalignment in non-planar conjugated systems. *Chem. Phys. Lett.* **125**, 231–234 (1986).

S14 Cardona, C. M., Li, W., Kaifer, A. E., Stockdale, D. & Bazan, G. C. Electrochemical considerations for determining absolute frontier orbital energy levels of conjugated polymers for solar cell applications. *Adv. Mater.* **23**, 2367–71 (2011).

S15 Roth, H. G., Romero, N. A. & Nicewicz, D. A. Experimental and Calculated Electrochemical Potentials of Common Organic Molecules for Applications to Single-Electron Redox Chemistry. *Synlett* **27**, 714–723 (2015).
